# Potential health impact of therapeutic HPV vaccines in China: a modeling study

**DOI:** 10.1186/s12916-026-05050-0

**Published:** 2026-07-07

**Authors:** Chenghao Pan, Shangying Hu, Tingting You, Meng Gao, Yanling Zhong, Xinyuan Wang, Youlin Qiao, Xuelian Zhao, Fanghui Zhao, Mark Jit

**Affiliations:** 1 National Clinical Research Center for Cancer/Cancer Hospital, National Cancer Center, Chinese Academy of Medical Sciences and Peking Union Medical College, Beijing, China; 2 Scientific Research Center, Friendship Hospital, Beijing, China; 3 Department of Epidemiology, Beijing Tiantan Hospital, Beijing Neurosurgical Institute, Capital Medical University, Beijing, China; 4 West China School of Public Health, Sichuan University, Chengdu, China; 5 School of Population Medicine and Public Health, Chinese Academy of Medical Sciences and Peking Union Medical College, Beijing, China; 6 Department of Infectious Disease Epidemiology, School of Global Public Health, New York University, New York, USA

**Keywords:** Therapeutic HPV vaccines, Health impact, Cervical intraepithelial neoplasia, Cervical cancer, Modeling study

## Abstract

**Background:**

Therapeutic HPV vaccines (TxVs) may complement existing cervical cancer prevention strategies, particularly for women already infected with HPV who derive limited benefit from prophylactic vaccination. As China advances toward the WHO 90–70–90 cervical cancer elimination targets, understanding the potential health impact of therapeutic vaccines under varying implementation scenarios is critical.

**Methods:**

We developed a two-stage hybrid model combining a dynamic HPV transmission model with a natural history model of HPV-related disease progression to project the long-term health impact of therapeutic HPV vaccines in China from 2025 to 2125. Two vaccine types aligned with WHO Preferred Product Characteristics were evaluated: one targeting HPV infection clearance (TxV1) and one targeting treatment of cervical intraepithelial neoplasia (CIN) grade 2 or 3 (TxV2). Vaccination was modelled under two use cases: population-based delivery and integration into existing national screening programmes. Outcomes included cervical cancer cases and deaths averted, relative reductions compared with no therapeutic vaccination, age-standardised incidence, and the projected year of elimination. One-way and probabilistic sensitivity analyses evaluate model uncertainty.

**Results:**

In the base case, screening-integrated TxV1 (Use Case 2) could avert up to 834.0 thousand cervical cancer cases (12.7% reduction) and accelerate elimination by 11 years, while population-based TxV1 could avert 169.6–474.0 thousand cases (2.6–7.2% reduction). TxV2 showed more modest effects (2.4–7.4% reduction) and was highly sensitive to conventional CIN treatment coverage. Therapeutic vaccine impact was greater in scenarios with limited scale-up of prophylactic vaccination. Vaccine efficacy, coverage, genotype spectrum, and rollout timing were key drivers of impact.

**Conclusions:**

Therapeutic HPV vaccines with substantial efficacy could reduce the cervical cancer burden in China, particularly during the transition toward full-scale prophylactic vaccination and screening. These findings support continued investment in therapeutic HPV vaccine development and provide evidence to inform national strategy planning aligned with elimination goals.

**Supplementary Information:**

The online version contains supplementary material available at https://doi.org/10.1186/s12916-026-05050-0.

## Background

Cervical cancer remains one of the most common cancers among women, with approximately 600,000 new cases and over 300,000 deaths in 2022 worldwide [1]. Persistent infection with high-risk human papillomavirus (hrHPV) is the necessary cause of cervical cancer, and the disease is largely preventable through HPV vaccination and effective screening and treatment of precancerous lesions [2, 3]. In 2020, the World Health Organization (WHO) launched the global strategy to accelerate the elimination of cervical cancer as a public health problem, establishing the “90-70-90” targets to be achieved by 2030: 90% of girls fully vaccinated with HPV vaccine by age 15, 70% of women screened at least twice by age 45, and 90% of screen-positive women receiving appropriate treatment [4]. In 2025, China announced that HPV vaccination would be included in the National Immunization Program (NIP) [5]. However, nationwide implementation will take time, and many cohorts of adult women will remain unvaccinated for years to come [6]. Current national vaccine coverage remains suboptimal, with the most recent statistics available suggesting 10.15% of women aged 9–45 years in 2022 [7]. While prophylactic HPV vaccines are highly effective in preventing new HPV infections and precancerous lesions, they do not clear existing infections [8]. Therefore, unvaccinated women continue to face substantial risk of HPV infection and progression to cervical cancer.

Screening serves as an essential secondary prevention strategy. In China, the implementation of the national cervical cancer and breast cancer screening program was initiated in 2009 and has since led to a substantial increase in screening coverage. At the national level, the lifetime cervical cancer screening rate among women aged 20 years and above increased from 20.7% in 2010 to 36.8% in 2024. Among women aged 35–64 years, the target age group for the national screening programme, lifetime screening coverage reached 51.5% in 2024 [9–11]. However, screening rates remain relatively low in rural and central-western regions [10]. Limited access to diagnostic services and the absence of simple, user-friendly screening tools in low-resource areas hinder the timely follow-up and appropriate treatment of screen-positive women, resulting in inadequate treatment coverage and compromising overall program effectiveness [12]. Although ablative treatment may be used for appropriately selected lesions, excisional treatment remains the Chinese guideline-recommended preferred approach for CIN2/3 [13] and has been associated with increased obstetric risks [14, 15]. Therefore, there is an urgent need for less invasive, fertility-preserving treatment options.

Therapeutic HPV vaccines, currently in development, are designed to clear established HPV infections or treat precancerous lesions [16], with most candidates designed to induce cell-mediated immune responses against HPV E6 and/or E7 antigens [17]. Current clinical studies commonly evaluate endpoints such as HPV clearance, histological regression of CIN2/3 or high-grade squamous intraepithelial lesions [17]. These endpoints correspond closely to the two product concepts outlined in the WHO Preferred Product Characteristics (PPCs) for therapeutic HPV vaccine: vaccines that clear hrHPV infections in HPV-positive women, and vaccines that treat CIN2/3 lesions detected through screening [18]. In addition, a technical modelling report from the Daffodil Centre explored the potential public health value and desirable attributes of future therapeutic HPV vaccines across 78 low- and middle-income countries [19], and a modelling study from Uganda suggested that therapeutic vaccination could contribute to cervical cancer control under certain implementation scenarios [20]. Therapeutic HPV vaccines may serve as a critical complement to existing preventive strategies, especially for women beyond the age of prophylactic vaccination or in regions with limited screening infrastructure. However, no published studies to date have comprehensively evaluated the population-level health impact of therapeutic HPV vaccines in China, a country with the largest global burden of cervical cancer.

As China accelerates progress toward the 90-70-90 goals, evaluating how therapeutic HPV vaccines could be strategically integrated into the national cervical cancer control framework is of critical importance. Given the relatively late date of nationwide vaccine introduction in China compared to many other countries, therapeutic vaccine options may be of particular benefit in this setting. In this study, we simulated multiple implementation scenarios across varying levels of prophylactic vaccination and screening coverage, and examined the potential of two types of therapeutic vaccines: one targeting HPV infection clearance (TxV1), and one targeting CIN2/3 treatment (TxV2). Sensitivity analyses were also conducted to identify key parameters influencing health outcomes and to inform future vaccine development and policy implementation. Our findings aim to support decision-makers and product developers in understanding the potential role of therapeutic HPV vaccines within the context of China’s cervical cancer elimination agenda.

## Methods

### Overview

We previously developed a two-stage hybrid model that simulates the sexual transmission of HPV and the natural history of cervical cancer [21, 22]. In this study, we extended the model to incorporate therapeutic HPV vaccines to evaluate their projected health outcomes across various scenarios and therapeutic vaccine characteristics. The analysis included all Chinese women living or expected to be born between 2025 and 2125. The population was categorized based on area of residence (urban or rural), level of sexual activity (high, low, or none), and age group (yearly age classes from 0 to 84, and 85+). We utilized parameters derived from published literature and publicly available government datasets (see Additional File 1 p 4–11 for more details).

### Therapeutic vaccine assumptions and vaccination strategies

Based on the WHO PPCs for Therapeutic HPV Vaccines, we modeled two types of therapeutic vaccines. Type 1 (TxV 1) was designed to clear oncogenic HPV infections, while Type 2 (TxV 2) targeted cervical precancerous lesions (CIN2/3) [18]. In the base case, therapeutic vaccines were introduced in 2030, which focused on HPV-16 and 18, prioritized as the first generation of therapeutic vaccines in the WHO PPCs. Because no therapeutic HPV vaccine has yet been licensed and product-specific clinical efficacy data remain limited, the base-case efficacy assumptions were based on the WHO PPCs and should be interpreted as target-product-profile assumptions rather than empirically established efficacy estimates. In the base case, we assumed a two-dose schedule with 90% efficacy for two doses and 70% efficacy for one dose. Coverage was assumed to reach 90% for at least one dose and 70% for full two-dose coverage. We evaluated two distinct Use Cases for therapeutic vaccination. In Use Case 1, only TxV1 was considered, delivered annually through a population-based approach targeting women aged 30 years, with or without an initial catch-up campaign for women aged 31–45 years in the first year of roll-out. In Use Case 2, therapeutic vaccination was integrated into HPV-based cervical cancer screening programs, targeting women aged 35–64 years. In this case, TxV 1 was administered to women with a positive HPV test but negative triage result, while TxV 2 was administered to those diagnosed with CIN2/3 following a positive HPV test and histopathological confirmation (Table 1). The two Use Cases were designed as distinct implementation pathways, not directly comparable or dose-equivalent strategies. Results are therefore presented separately to illustrate the projected impact of each strategy under its own implementation assumptions.

**Table 1:** Key assumptions of therapeutic vaccines

Therapeutic vaccine	Base case	Sensitivity analysis
Indication	**Type 1(TxV1)**: Clear oncogenic HPV infections **Type 2(TxV2)**: Treat cervical precancerous lesions (CIN2/3)	
Use Case and target population	**Use Case 1**: Delivered through a population-based vaccination program targeting women aged 30 years, with or without a one-time catch-up campaign for women aged 31–45 in the first year of implementation. **Use Case 2**: Integrated into HPV-based cervical cancer screening programs. TxV1 administered to women with a positive HPV test and a negative triage test in cervical cancer screening. TxV2 administered to women diagnosed with CIN2/3 following a positive HPV test and histopathological confirmation	
Schedule and coverage	2-dose schedule. 90% of target population receive at least one dose and 70% of target population receive two doses (90%/70%)	-100% /100% -50% /50%
Vaccine efficacy against infection (TxV1) or lesions (TxV2)	90% for two doses and 70% for one dose (90%/70%)	-70%/50% -50%/0%
Year of therapeutic vaccine introduction	2030	-2027 -2035
Targeted HPV types	HPV 16,18	- HPV 16,18,52,58 -HPV 16,18,52,58,31,33,35,45

Given the absence of licensed therapeutic HPV vaccines and limited evidence on the duration and strength of vaccine-induced immune memory, we adopted a conservative assumption regarding long-term vaccine effects. Therapeutic vaccination was assumed to act only on the target HPV infection or CIN2/3 lesion present at the time of vaccination, according to the assumed vaccine efficacy. We did not assume sustained immunity after therapeutic vaccination. Therefore, women whose infection or lesion was cleared or successfully treated by therapeutic vaccination remained susceptible to subsequent reinfection and disease progression according to the same modelled natural history assumptions as other women(see Additional File 1 p 4–5 for more details).

### Vaccination and screening scenarios, inputs and assumption

Given that the Chinese government has announced the inclusion of HPV vaccination in the NIP, we modeled domestically produced bivalent HPV vaccine in our analysis and its efficacy was assumed based on published literature [23]. We modeled a 2-dose vaccination schedule targeting 13-year-old girls and evaluated various coverage levels. Based on experiences from other countries, vaccine coverage is expected to increase substantially within the first five years after introduction [24, 25]. Therefore, in the base case, we assumed that HPV vaccine coverage among 13-year-old girls would increase linearly from 2025 to 2030, reaching 90% and remaining constant thereafter (PxV 2030). Additionally, we included two more pessimistic scenarios to reflect potential limitations in vaccine supply or demand: taking until 2050 to reach 90% coverage (PxV 2050) and remaining at 5% without further increase (PxV status quo) [7].

Since therapeutic HPV vaccines rely on HPV testing to identify target populations, this study primarily considers HPV-based cervical cancer screening. In this study, HPV-based screening is assumed to be conducted every five years for women aged 35–64. The screening coverage in the base case remains unchanged (Screening status quo), based on data published by the Chinese Center for Disease Control and Prevention [10]. Aligned with the “Action Plan for Accelerating the Elimination of Cervical Cancer (2022–2030)”, we established two scenarios for increasing screening coverage: HPV2030 and HPV2050 [26]. Starting in 2025, screening coverage is projected to expand gradually, increasing linearly from the status quo in 2025 to 70% by 2030 (HPV2030) and 2050 (HPV2050), respectively. For women aged 21–34 and those over 65, these groups represent the population undergoing opportunistic screening, with their screening coverage rates remaining the same as the status quo across all scenarios (Additional File 1: Fig. S7-10).

The model was stratified by area of residence to capture urban-rural differences in demographics, baseline disease burden, and management of screening and treatment. Rural populations were assigned lower screening-related follow-up and treatment parameters than urban populations to reflect more limited access to diagnostic and treatment services (Additional File 1: Table S1). We assumed the same future coverage trajectories for prophylactic vaccination, therapeutic vaccination, and screening scale-up between urban and rural populations, following the same national coverage assumptions under each scenario. Thus, urban–rural differences in projected outcomes reflect underlying residence-specific characteristics.

### Outcomes and analysis

For this study, we evaluated the health impact of different types of therapeutic vaccines under various scenarios over a 100-year time horizon (2025–2125). The main outcomes were cervical cancer cases and deaths averted by therapeutic vaccination, relative reductions in cases and deaths, and age-standardised cervical cancer incidence. Outcomes were compared with TxV0, defined as existing screening and conventional CIN2/3 treatment without therapeutic HPV vaccination. Cancers attributable to HPV types not targeted by therapeutic vaccination remained included in the overall incidence estimates.The projected year of cervical cancer elimination was defined as the first year in which the total age-standardised cervical cancer incidence, including cancers attributable to all modelled high-risk HPV types, fell below the WHO elimination threshold of 4 cases per 100,000 women. We also calculated the number of therapeutic vaccine doses needed to avert one cervical cancer case (NNV) during 2025–2100, defined as the total number of therapeutic vaccine doses administered divided by the corresponding number of cervical cancer cases averted.

We did one-way sensitivity analysis by varying the characteristics of therapeutic vaccine and parameters of screening and treatment to quantify their effects on the health impact of therapeutic vaccines. Characteristics of therapeutic vaccine comprised the vaccine efficacy (70%/50%, 50%/0%), coverage (100%/100%, 50%/50%), targeted HPV types (HPV-31, 33, 35, 45, 52, and 58) and rollout years (2027 and 2035). Screening and treatment parameters comprised screening sensitivity, conventional treatment rate and conventional treatment efficacy against CIN2 and CIN3 (Additional File 1: Table S1). Probabilistic sensitivity analysis was conducted by performing 500 Monte Carlo simulations to sample parameter values from their distributions and estimate outcomes. Results are presented as medians with 80% uncertainty intervals [UIs] (i.e., 10th–90th percentiles). All analyses were performed in R. The results were reported following the HPV-FRAME checklist and CHEERS checklist (Additional File 1: Table S3-5).

## Results

**Fig. 1:**
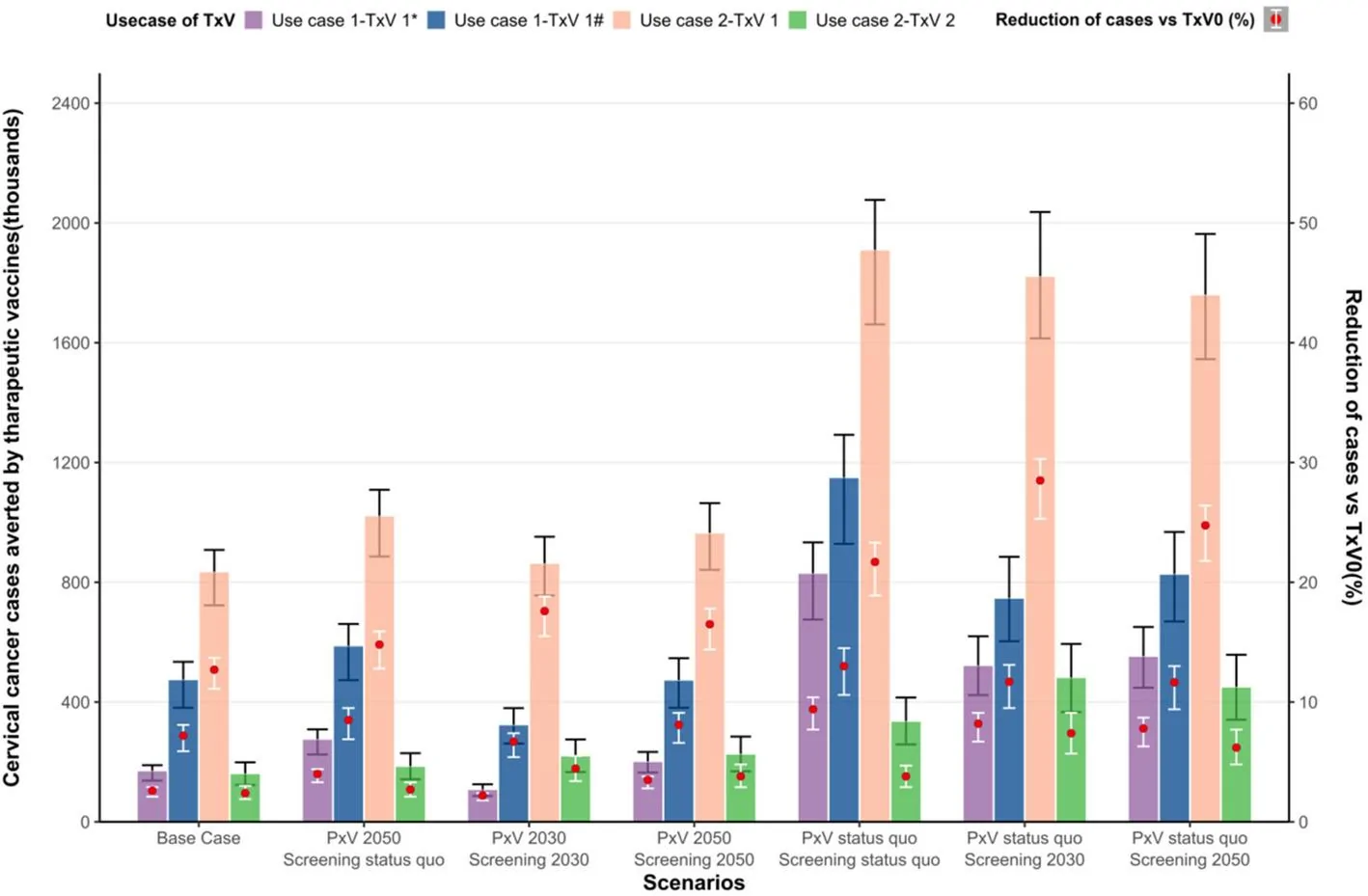
Cervical cancer cases averted by therapeutic HPV vaccination and percentage reduction across different scenarios (thousands). Bars show the projected number of cervical cancer cases averted by therapeutic HPV vaccination (TxV) under different implementation and background scenarios, including combinations of prophylactic HPV vaccination (PvV) status and screening coverage. Purple, blue, yellow and green bars represent different Use Cases. Dots in red denotes the percentage reduction in cervical cancer cases relative to the scenario without therapeutic vaccination (TxV0). All values are depicted as the median of Monte Carlo simulations, and error bars indicate the 80% uncertainty intervals (10th - 90th percentiles) from 500 Monte Carlo simulations. Base case = two-dose prophylactic HPV vaccine coverage among 13-year-old girls increases linearly to 90% by 2030, while screening coverage remains unchanged. Status quo = current prophylactic HPV vaccination or/and screening&treatment practices; PxV 2030/2050 = two-dose prophylactic HPV vaccine coverage among 13-year-old girls increases linearly to 90% by 2030/2050; Screening 2030/2050 = HPV DNA-based screening coverage increases linearly to 70% by 2030/2050; TxV0 = no therapeutic vaccination, with screen-detected CIN2/3 managed according to existing conventional treatment pathways; Use Case 1 (TxV1*) = population-based vaccination with a type 1 therapeutic vaccine targeting clearance of oncogenic HPV infections, administered annually to women aged 30 years; Use Case 1 (TxV1#) = same as TxV1*, with an initial catch-up campaign for women aged 31-45 years in the first year of roll-out; Use Case 2 (TxV1) = screening-based vaccination with a type 1 therapeutic vaccine targeting HPV-positive, triage-negative women; Use Case 2 (TxV2) = screening-based vaccination with a type 2 therapeutic vaccine targeting women diagnosed with CIN2/3 through HPV-based screening

Table 2; Fig. 1 presents the estimated number of cervical cancer cases and the corresponding cases averted across seven vaccination and screening scenarios in China, under five therapeutic vaccination strategies: no therapeutic vaccination (TxV0), two population-based vaccination strategies with TxV1 (Use Case 1), and two screening-integrated vaccination strategies with TxV1 and TxV2 (Use Case 2). The health impact of therapeutic vaccines varied notably between Use Cases. In the base case (PxV 2030, Screening status quo), TxV1 averted 169.6–834.0 thousand cervical cancer cases across Use Cases (2.6–12.7% reduction), while TxV2 averted 160.4 thousand cases (2.4% reduction) in Use Case 2. In contrast, in the most pessimistic scenario (PxV status quo, Screening status quo), the projected impact of therapeutic vaccination increased substantially: TxV1 averted 829.3-1908.8 thousand cases (9.4–21.7% reduction), and TxV2 averted 335.1 thousand cases (3.8% reduction). Therapeutic vaccines also demonstrated a similar impact on cervical cancer deaths, with detailed results provided in the supplementary file (Additional File 1: Table S11-16). The additional dose-standardised analysis showed that Use Case 2 had substantially lower NNV values than Use Case 1(Additional File 1: Table S19).

**Table 2:** Estimated cervical cancer cases across different scenarios in China(thousands)

Scenario	Use Case	Type of TxV	Cervical cancer cases				
			2025–2050	2051–2075	2076–2100	2101–2125	2025–2125
Base case (PxV 2030, Screening status quo)							
	-	TxV 0	2971.3 (2894.2–3103.1)	1932.2 (1871.9–2043.1)	997.7 (965.4–1055.2)	633.9 (609.4–676.0)	6532.9 (6338.6–6875.1)
	1	TxV 1^*^	2931.1 (2856.0–3063.1)	1835.0 (1772.5–1932.5)	975.5 (941.9–1031.1)	632.8 (608.4–674.8)	6373.4 (6183.5–6701.8)
	1	TxV 1^#^	2807.4 (2724.5–2930.4)	1707.2 (1635.9–1804.6)	939.0 (902.7–992.6)	632.5 (608.1–674.4)	6089.9 (5878.2–6402.3)
	2	TxV 1	2810.6 (2729.2–2937.4)	1467.0 (1392.6–1562.7)	816.8 (779.5–868.8)	629.8 (605.3–671.1)	5734.6 (5518.7–6051.8)
	2	TxV 2	2893.2 (2821.9–3023.1)	1858.9 (1801.3–1964.7)	985.5 (954.2–1044.2)	633.7 (609.2–675.7)	6368.9 (6189.6–6695.1)
**PxV 2050**,** Screening status quo**							
	-	TxV 0	2977.6 (2900.6–3108.4)	2074.8 (2011.7–2192.9)	1132.4 (1097.4–1195.5)	678.0 (652.9–720.4)	6860.4 (6669.7–7221.3)
	1	TxV 1^*^	2937.1 (2861.9–3068.7)	1924.5 (1855.9–2031.0)	1074.0 (1035.5–1132.4)	667.2 (642.2–709.2)	6598.7 (6398.6–6930.2)
	1	TxV 1^#^	2813.2 (2729.9–2936.2)	1793.4 (1716.1–1899.5)	1035.0 (993.9–1094.3)	666.0 (640.6–707.4)	6313.8 (6088.5–6640.1)
	2	TxV 1	2816.3 (2735.3–2943.7)	1554.2 (1474.1–1659.4)	861.6 (818.4–919.2)	642.8 (617.2–684.3)	5882.2 (5652.1–6212.5)
	2	TxV 2	2899.4 (2827.8–3029.6)	1990.0 (1929.7–2107.8)	1110.7 (1076.4–1172.9)	676.5 (651.5–719.0)	6674.4 (6490.6–7019.8)
**PxV 2030**,** Screening 2030**							
	-	TxV 0	2631.6 (2530.2–2809.6)	1264.0 (1190.6–1394.3)	643.0 (609.8–697.3)	358.7 (332.8–399.7)	4892.4 (4665.4–5297.0)
	1	TxV 1^*^	2601.7 (2500.1–2778.3)	1205.6 (1137.0–1326.7)	626.9 (593.4–681.3)	357.8 (332.1–399.1)	4790.6 (4567.1–5190.5)
	1	TxV 1^#^	2501.2 (2405.8–2667.6)	1131.7 (1058.5–1242.5)	596.0 (559.5–650.9)	357.4 (331.8–398.9)	4589.6 (4358.9–4946.2)
	2	TxV 1	2445.0 (2343.3–2612.0)	813.1 (743.9–911.9)	442.8 (406.7–493.3)	353.6 (328.6–395.6)	4064.9 (3830.3–4410.3)
	2	TxV 2	2520.5 (2427.5–2696.4)	1162.3 (1094.3–1294.0)	629.3 (597.8–685.6)	358.6 (332.6–399.5)	4674.6 (4453.3–5073.5)
**PxV 2050**,** Screening 2050**							
	-	TxV 0	3063.1 (2974.9–3213.2)	1635.5 (1553.9–1783.2)	735.6 (697.3–802.0)	398.9 (373.2–442.1)	5832.8 (5607.6–6243.1)
	1	TxV 1^*^	3026.5 (2940.2–3177.8)	1525.1 (1444.4–1657.8)	700.1 (661.2–764.9)	389.2 (363.4–432.6)	5644.0 (5414.9–6027.3)
	1	TxV 1^#^	2900.4 (2810.1–3039.7)	1429.8 (1345.1–1558.6)	667.4 (624.5–729.6)	387.9 (362.2–431.2)	5382.1 (5145.0–5753.2)
	2	TxV 1	2901.5 (2809.6–3044.9)	1154.5 (1067.5–1277.8)	482.2 (440.7–539.2)	358.6 (333.2–401.3)	4905.5 (4664.2–5272.7)
	2	TxV 2	2975.8 (2894.5–3125.3)	1523.5 (1446.6–1678.8)	708.1 (671.8–776.4)	397.2 (371.7–440.2)	5604.9 (5385.2–6012.3)
**PxV status quo**,** Screening status quo**							
	-	TxV 0	2982.3 (2905.3–3113.3)	2292.6 (2223.3–2421.9)	1851.0 (1793.3–1952.8)	1631.0 (1579.1–1718.8)	8754.0 (8504.8–9207.8)
	1	TxV 1^*^	2941.3 (2866.2–3073.0)	2074.9 (1997.7–2186.6)	1574.7 (1505.4–1669.2)	1373.2 (1308.8–1456.2)	7975.7 (7687.1–8379.3)
	1	TxV 1^#^	2817.5 (2734.1–2940.6)	1939.2 (1852.6–2054.0)	1531.9 (1457.7–1624.9)	1369.7 (1305.0–1452.5)	7657.8 (7354.3–8073.4)
	2	TxV 1	2820.9 (2739.8–2948.4)	1713.5 (1625.5–1833.4)	1257.3 (1184.9–1354.8)	1101.7 (1038.2–1189.2)	6897.6 (6589.8–7305.1)
	2	TxV 2	2903.9 (2832.3–3034.4)	2190.4 (2124.9–2322.8)	1766.5 (1712.6–1873.8)	1554.8 (1507.0–1647.3)	8413.6 (8177.3–8881.3)
**PxV status quo**,** Screening 2030**							
	-	TxV 0	2642.5 (2540.3–2820.6)	1528.3 (1440.9–1684.7)	1191.7 (1119.1–1311.8)	1043.4 (977.4–1149.0)	6403.4 (6080.4–6975.9)
	1	TxV 1^*^	2611.7 (2510.1–2788.0)	1390.2 (1302.3–1528.0)	1025.6 (952.0–1128.0)	885.2 (820.2–976.2)	5905.1 (5591.1–6408.7)
	1	TxV 1^#^	2511.3 (2415.6–2678.1)	1311.5 (1220.1–1443.0)	986.5 (913.8–1091.1)	882.5 (817.3–973.1)	5695.1 (5367.7–6183.0)
	2	TxV 1	2456.0 (2353.7–2623.5)	965.5 (880.6–1076.5)	639.5 (577.9–724.9)	554.4 (500.6–628.5)	4627.4 (4320.9–5051.4)
	2	TxV 2	2530.9 (2437.9–2707.4)	1384.2 (1301.6–1546.3)	1070.4 (1005.2–1196.2)	934.1 (874.5–1044.5)	5919.6 (5617.4–6504.9)
**PxV status quo**,** Screening 2050**							
	-	TxV 0	3067.9 (2979.6–3218.2)	1811.5 (1720.2–1975.0)	1205.3 (1131.9–1328.1)	1043.5 (977.4–1149.1)	7123.3 (6814.8–7676.4)
	1	TxV 1^*^	3030.9 (2944.5–3182.2)	1649.6 (1557.8–1794.5)	1039.2 (964.2–1144.2)	885.2 (820.2–976.3)	6597.9 (6294.4–7081.4)
	1	TxV 1^#^	2904.7 (2814.3–3044.1)	1550.8 (1456.7–1692.7)	999.7 (925.6–1107.5)	882.5 (817.4–973.2)	6344.8 (6014.9–6816.7)
	2	TxV 1	2906.1 (2814.2–3049.6)	1271.0 (1174.7–1405.0)	670.3 (607.1–761.8)	556.8 (502.7–631.3)	5409.4 (5106.5–5839.5)
	2	TxV 2	2980.4 (2899.1–3130.1)	1673.8 (1587.6–1849.0)	1083.7 (1017.5–1212.5)	934.2 (874.6–1044.6)	6671.7 (6380.8–7240.0)

Base case = two-dose prophylactic HPV vaccine coverage among 13-year-old girls increases linearly to 90% by 2030, while screening coverage remains unchanged

Status quo = current prophylactic HPV vaccination or/and screening&treatment practices; PxV 2030/2050 = two-dose prophylactic HPV vaccine coverage among 13-year-old girls increases linearly to 90% by 2030/2050; Screening 2030/2050 = HPV DNA-based screening coverage increases linearly to 70% by 2030/2050; TxV0 = no therapeutic vaccination, with screen-detected CIN2/3 managed according to existing conventional treatment pathways; Use Case 1 (TxV1*) = population-based vaccination with a type 1 therapeutic vaccine targeting clearance of oncogenic HPV infections, administered annually to women aged 30 years; Use Case 1 (TxV1#) = same as TxV1*, with an initial catch-up campaign for women aged 31–45 years in the first year of roll-out; Use Case 2 (TxV1) = screening-based vaccination with a type 1 therapeutic vaccine targeting HPV-positive, triage-negative women; Use Case 2 (TxV2) = screening-based vaccination with a type 2 therapeutic vaccine targeting women diagnosed with CIN2/3 through HPV-based screening

The age-stratified subgroup analysis showed that the distribution of benefits variedby Use Cases. In the base-case scenario, cervical cancer cases averted by population-based TxV1 without catch-up were concentrated mainly among women aged 35–54 years, accounting for 68.7% of total averted cases. In contrast, the benefits of population-based TxV1 with catch-up and screening-integrated TxV1/TxV2 were concentrated primarily among women aged 45 years and older, accounting for 80.6%, 93.7%, and 86.7% of total averted cases, respectively. Notably, for screening-integrated TxV1, the largest share of averted cases occurred among women aged ≥ 65 years, accounting for 41.6% of total averted cases (Additional File 1: Table S6 and Table S12). Urban-rural stratified results showed that therapeutic vaccination provided benefits in both populations, with patterns varying by strategy. Relative reductions in cervical cancer cases were broadly comparable between urban and rural populations for population-based TxV1, mixed across scenarios for screening-integrated TxV1, and consistently higher in rural populations for TxV2. (Additional File 1: Table S7,8,10,13,14,15,16).

In Use Case 1, the number of cervical cancer cases averted ranged from107.3 thousand to 829.3 thousand for single-age cohort vaccination strategy and from 323.2 thousand to 1148.8 thousand for catch-up strategy across different scenarios. For both strategies, the largest relative reductions—9.4% for single-age cohort vaccination and 13.0% for catch-up vaccination—were observed under the status quo scenario. In contrast, the smallest reductions—2.2% and 6.7%, respectively—were observed under the PxV 2030 with Screening 2030 scenario (Fig. 1). In Use Case 2, where therapeutic vaccination was integrated into screening programs, compared to no therapeutic vaccination, TxV1 could avert between 834.0 and 1908.8 thousand cases, while TxV2 could avert 160.4 to 481.0 thousand cases across different scenarios (Fig. 1, Additional File 1 Table S9).

**Fig. 2:**
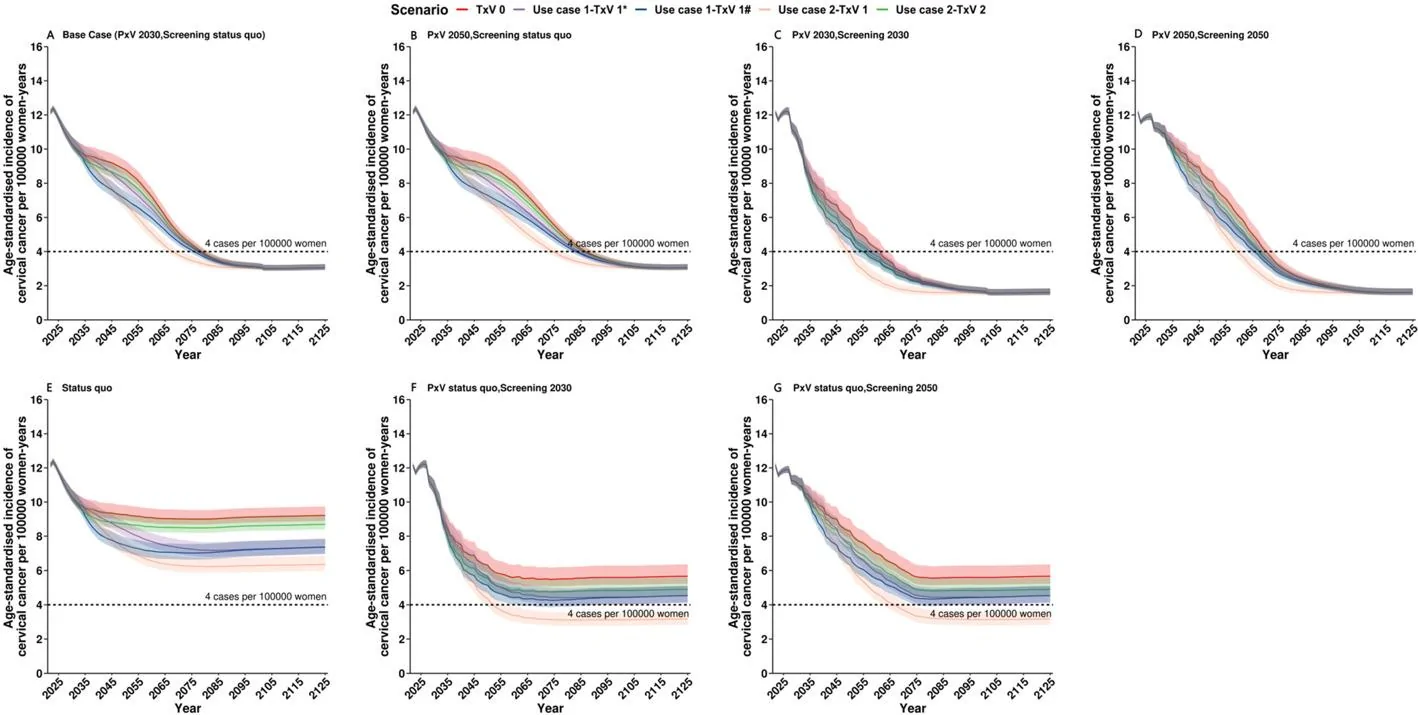
Age-standardised annual cervical cancer incidence in China under different scenarios and types of therapeutic vaccines, 2025-2125. Panels A–G show the projected trajectories of age-standardised cervical cancer incidence per 100 000 women under different combinations of prophylactic HPV vaccination (PvV) status and screening coverage. The line in red, purple, blue, yellow and green represent TxV0, Use Case 1 (TxV1*), Use Case 1 (TxV1#), Use Case 2 (TxV1) and Use Case 2 (TxV2) respectively. Shaded areas indicate the 80% uncertainty intervals (10th - 90th percentiles) from 500 Monte Carlo simulations. The horizontal dashed line marks the WHO cervical cancer elimination threshold (4 cases per 100 000 women). Base case = two-dose prophylactic HPV vaccine coverage among 13-year-old girls increases linearly to 90% by 2030, while screening coverage remains unchanged. Status quo = current prophylactic HPV vaccination or/and screening&treatment practices; PxV 2030/2050 = two-dose prophylactic HPV vaccine coverage among 13-year-old girls increases linearly to 90% by 2030/2050; Screening 2030/2050 = HPV DNA-based screening coverage increases linearly to 70% by 2030/2050; TxV0 = no therapeutic vaccination, with screen-detected CIN2/3 managed according to existing conventional treatment pathways; Use Case 1 (TxV1*) = population-based vaccination with a type 1 therapeutic vaccine targeting clearance of oncogenic HPV infections, administered annually to women aged 30 years; Use Case 1 (TxV1#) = same as TxV1*, with an initial catch-up campaign for women aged 31-45 years in the first year of roll-out; Use Case 2 (TxV1) = screening-based vaccination with a type 1 therapeutic vaccine targeting HPV-positive, triage-negative women; Use Case 2 (TxV2) = screening-based vaccination with a type 2 therapeutic vaccine targeting women diagnosed with CIN2/3 through HPV-based screening

Following the introduction of therapeutic HPV vaccines, the age-standardised annual incidence of cervical cancer in China showed a noticeable decline over approximately the first 30–40 years, compared to scenarios without therapeutic vaccination. The reduction was particularly pronounced in Use Case 2 involving TxV1, where a steeper decrease in incidence was observed. After this initial decline phase, the incidence curves gradually plateaued across all scenarios, indicating a slower rate of reduction in the long term (Fig. 2). Additionally, the number of cervical cancer cases averted by therapeutic vaccines decreased markedly in the later stages of the simulation period in scenarios that included prophylactic vaccination. For instance, under the PxV 2030 with Screening 2030 scenario, TxV1 in Use Case 2 averted approximately 205.6 thousand cases during 2076–2100, but only 4.0 thousand cases during 2101–2125. In terms of cervical cancer elimination, the annual age-standardised incidence is projected to fall below four cases per 100 000 women (the elimination threshold) by 2079 in the base case without therapeutic vaccination. Incorporating an infection-clearing therapeutic vaccine (TxV1) accelerated elimination by approximately 1–11 years across different Use Cases. The earliest elimination year, 2050, occurred in the PxV 2030 with Screening 2030 scenario when TxV1 was implemented in Use Case 2, representing a 12-year acceleration compared with no therapeutic vaccine (Fig. 2; Table 3).

**Table 3:** Projected year of cervical cancer elimination across different scenarios in China

Scenario	Use Case	Type of TxV	Projected elimination year: median, upper and lower limit of uncertainty range	Years of elimination advanced compared with no therapeutic vaccine
**Base case (PxV 2030, Screening status quo)**				
	0	TxV 0	2079 (2078–2082)	REF
	1	TxV 1^*^	2078 (2076–2081)	1 (1–2)
	1	TxV 1^#^	2076 (2074–2079)	3 (2–4)
	2	TxV 1	2068 (2066–2071)	11 (9–12)
	2	TxV 2	2078 (2077–2081)	1 (0–1)
**PxV 2050**,** Screening status quo**				
	0	TxV 0	2087 (2086–2091)	REF
	1	TxV 1^*^	2085 (2083–2088)	3 (2–4)
	1	TxV 1^#^	2084 (2081–2087)	4 (3–5)
	2	TxV 1	2075 (2072–2078)	13 (11–14)
	2	TxV 2	2087 (2085–2090)	1 (0–1)
**PxV 2030**,** Screening 2030**				
	0	TxV 0	2062 (2059–2065)	REF
	1	TxV 1^*^	2058 (2055–2062)	3 (2–4)
	1	TxV 1^#^	2055 (2052–2060)	6 (4–8)
	2	TxV 1	2050 (2048–2052)	12 (10–13)
	2	TxV 2	2057 (2054–2062)	4 (3–6)
**PxV 2050**,** Screening 2050**				
	0	TxV 0	2070 (2069–2073)	REF
	1	TxV 1^*^	2068 (2065–2071)	3 (2–4)
	1	TxV 1^#^	2066 (2063–2069)	5 (3–6)
	2	TxV 1	2060 (2058–2063)	11 (9–12)
	2	TxV 2	2068 (2066–2071)	2 (2–3)
**PxV status quo**,** Screening status quo**				
	0	TxV 0	Not achieved	REF
	1	TxV 1^*^	Not achieved	/
	1	TxV 1^#^	Not achieved	/
	2	TxV 1	Not achieved	/
	2	TxV 2	Not achieved	/
**PxV status quo**,** Screening 2030**				
	0	TxV 0	Not achieved	REF
	1	TxV 1^*^	Not achieved (Not achieved–2063)	/
	1	TxV 1^#^	Not achieved (Not achieved–2063)	/
	2	TxV 1	2052 (2049–2058)	/
	2	TxV 2	Not achieved	/
**PxV status quo**,** Screening 2050**				
	0	TxV 0	Not achieved	REF
	1	TxV 1^*^	Not achieved	/
	1	TxV 1^#^	Not achieved (Not achieved–2074)	/
	2	TxV 1	2067 (2062–2072)	/
	2	TxV 2	Not achieved	/

Cervical cancer elimination was defined as achieving an age-standardised incidence below 4 cases per 100 000 women. The elimination year refers to the first year in which this threshold was reached. Years of elimination advanced compared with no therapeutic vaccine indicate the number of years by which cervical cancer elimination is achieved earlier relative to the corresponding scenario without therapeutic vaccination. Positive values denote earlier elimination

Base case = two-dose prophylactic HPV vaccine coverage among 13-year-old girls increases linearly to 90% by 2030, while screening coverage remains unchanged

Status quo = current prophylactic HPV vaccination or/and screening&treatment practices; PxV 2030/2050 = two-dose prophylactic HPV vaccine coverage among 13-year-old girls increases linearly to 90% by 2030/2050; Screening 2030/2050 = HPV DNA-based screening coverage increases linearly to 70% by 2030/2050; TxV0 = no therapeutic vaccination, with screen-detected CIN2/3 managed according to existing conventional treatment pathways; Use Case 1 (TxV1*) = population-based vaccination with a type 1 therapeutic vaccine targeting clearance of oncogenic HPV infections, administered annually to women aged 30 years; Use Case 1 (TxV1#) = same as TxV1*, with an initial catch-up campaign for women aged 31–45 years in the first year of roll-out; Use Case 2 (TxV1) = screening-based vaccination with a type 1 therapeutic vaccine targeting HPV-positive, triage-negative women; Use Case 2 (TxV2) = screening-based vaccination with a type 2 therapeutic vaccine targeting women diagnosed with CIN2/3 through HPV-based screening

**Fig. 3:**
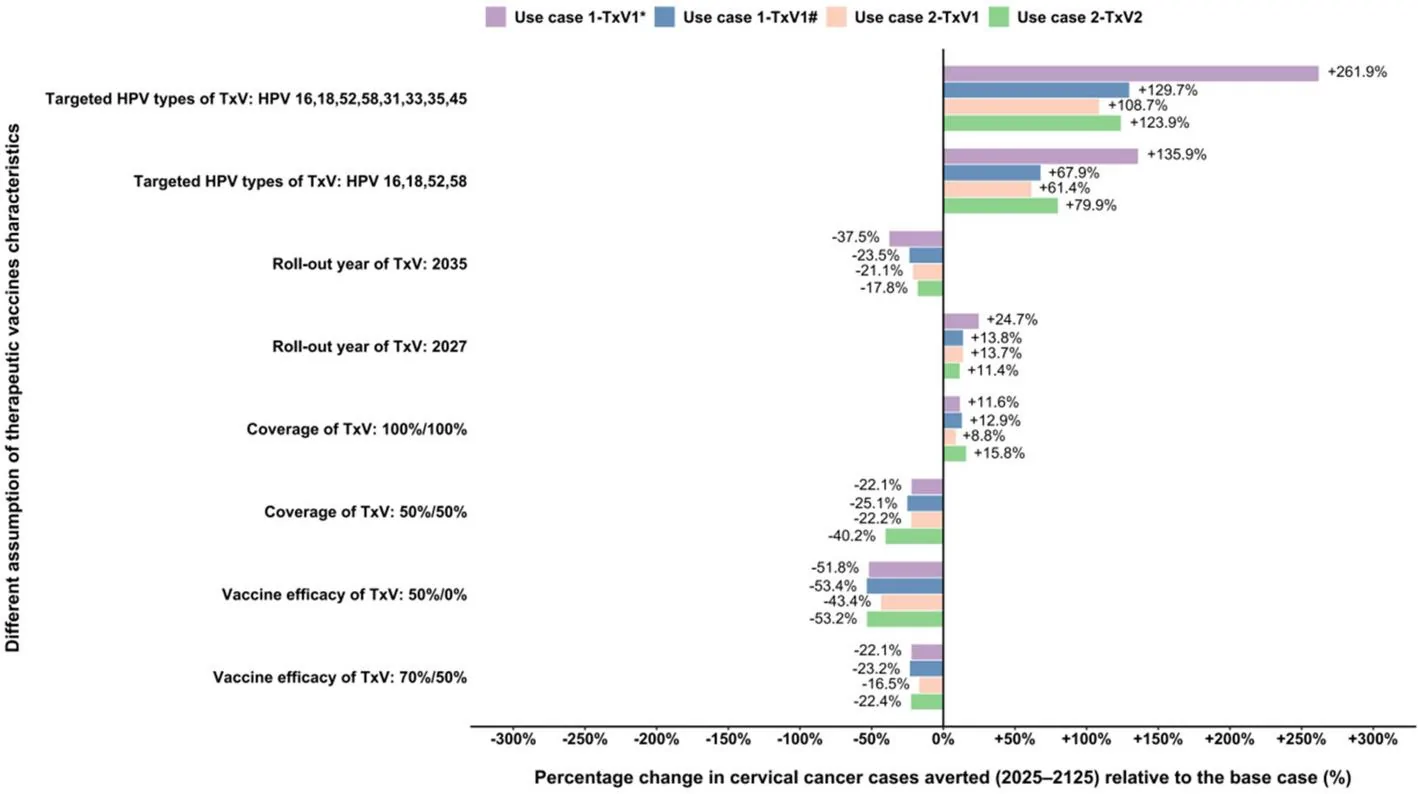
Sensitivity analysis of the effect of different assumptions about therapeutic vaccine characteristics on the percentage change in cervical cancer cases averted compared with the base case. Bars show the relative change of cervical cancer cases averted compared with the base case scenario. Purple and blue bars represent Use Case 1 (TxV1) with and without catch-up vaccination, respectively. Yellow and green bars represent Use Case 2 (TxV1 and TxV2). The vertical dashed line indicates the base-case estimate. Base case=two-dose prophylactic HPV vaccine coverage among 13-year-old girls increases linearly to 90% by 2030, while screening coverage remains unchanged, and therapeutic vaccine with 90% of efficacy for two doses and 70% for one dose, 70% of coverage for two doses and 20% for one dose, roll-out year of 2030 and targeted HPV infection or precancerous lesions related with HPV 16/18; TxV efficacy 70/50= therapeutic vaccine with 70% of efficacy for two doses and 50% for one dose; TxV efficacy 50/0= therapeutic vaccine with 50% of efficacy for two doses and 0% for one dose; TxV coverage 100/0= therapeutic vaccine with 100% of coverage for two doses; TxV coverage 50/0= therapeutic vaccine with 50% of coverage for two doses and 0% for one dose; TxV targeted HPV 16/18/52/58= therapeutic vaccine targeted HPV infection or precancerous lesions related with HPV 16/18/52/58; TxV targeted HPV 16/18/52/58/31/33/35/45= therapeutic vaccine targeted HPV infection or precancerous lesions related with HPV 16/18/52/58/31/33/35/45. Use Case 1 (TxV1*) = population-based vaccination with a type 1 therapeutic vaccine targeting clearance of oncogenic HPV infections, administered annually to women aged 30 years; Use Case 1 (TxV1#) = same as TxV1*, with an initial catch-up campaign for women aged 31-45 years in the first year of roll-out; Use Case 2 (TxV1) = screening-based vaccination with a type 1 therapeutic vaccine targeting HPV-positive, triage-negative women; Use Case 2 (TxV2) = screening-based vaccination with a type 2 therapeutic vaccine targeting women diagnosed with CIN2/3 through HPV-based screening

Figure 3 shows the results of one-way sensitivity analyses, representing the difference in cervical cancer cases averted compared to the base case scenario. The health impact of therapeutic vaccines were sensitive to several key parameters, including efficacy against HPV infection and precancerous lesions, vaccine coverage, year of roll-out, and the spectrum of targeted HPV types. A reduction in vaccine efficacy from 90% to 50% under a two-dose schedule led to 43.4%-53.4% of decrease in the number of cervical cancer cases averted between 2025 and 2125. Similarly, delaying the roll-out year by 5 years resulted in 17.8–37.5% of reduction in cases averted. Conversely, expanding the targeted HPV types to include additional high-risk HPV types (HPV 16/18/52/58) would increase by 61.4-135.9%.

**Fig. 4:**
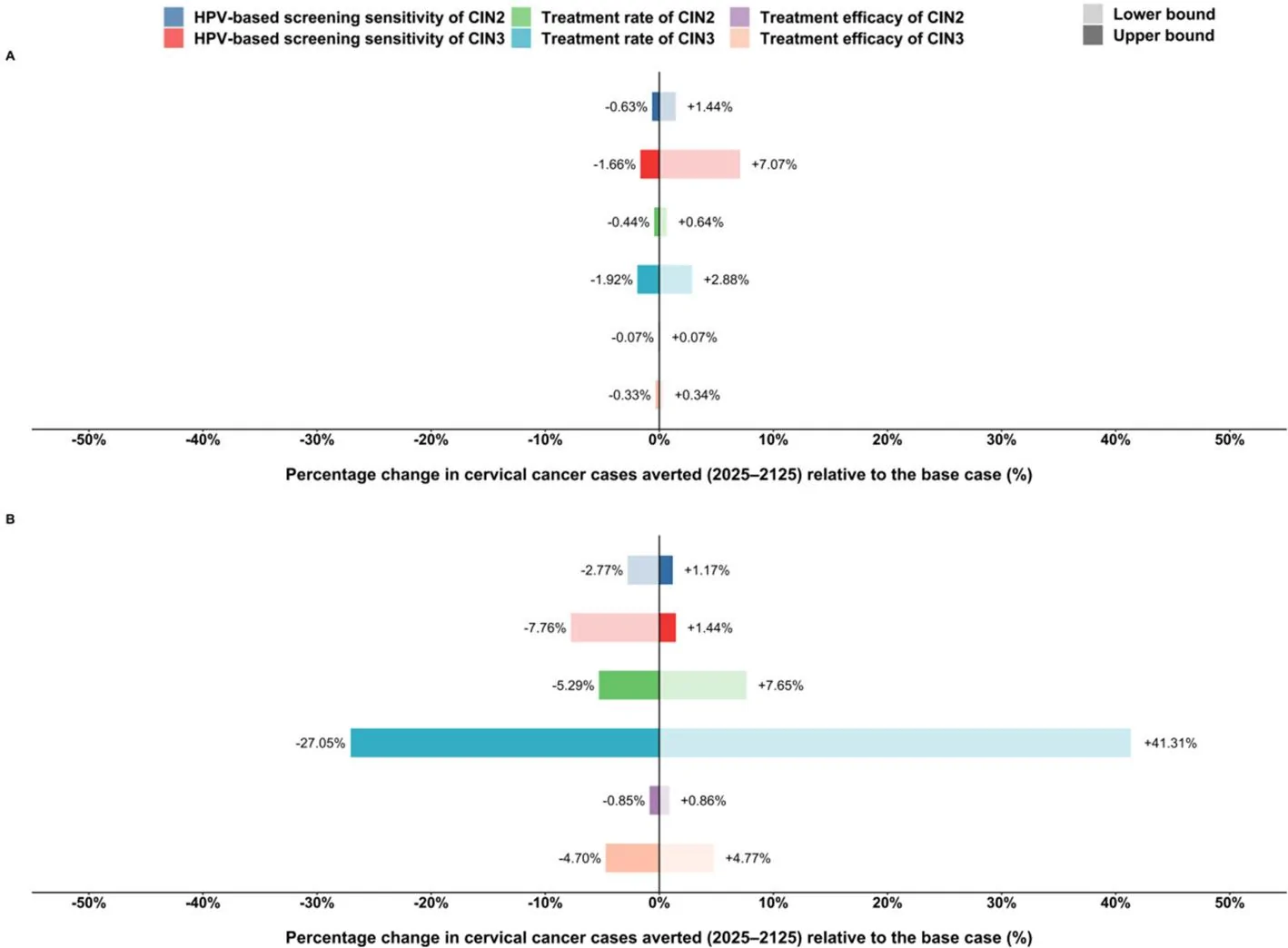
Sensitivity analysis of screening and treatment parameters influencing the cervical cancer cases averted by TxV1 (A) and TxV2 (B) in Use Case 2, 2025–2125. Bars show the percentage change in the number of cervical cancer cases averted compared with the base-case scenario when varying key screening and treatment parameters within their uncertainty ranges. Parameters include HPV-based screening sensitivity for CIN2 and CIN3 (blue-grey and red), treatment rates for CIN2 and CIN3 (green and turquoise), and treatment efficacy for CIN2 and CIN3 (purple and orange). Light-colored bars represent the lower bound of each parameter, and dark-colored bars represent the upper bound. The vertical dashed line indicates the base-case estimate. Positive values indicate increased cervical cancer cases averted, whereas negative values indicate reduced impact relative to the base case

Since Use Case 2 integrates therapeutic vaccination into existing cervical cancer screening programs, the health impact of therapeutic vaccines in this Use Case is also likely to be influenced by parameters related to screening sensitivity and treatment success rates. For TxV1, variations in screening sensitivity and treatment parameters had a relatively modest impact. Decreasing the HPV-based screening sensitivity for CIN3 detection led to a 7.07% increase in cases averted in TxV1. TxV2 was highly sensitive to the traditional treatment rates of CIN2 and CIN3, particularly CIN3. When the lowest traditional treatment rate for CIN3 was assumed, the number of cervical cancer cases averted by TxV2 increased by 41.3% (Fig. 4).

## Discussion

This study evaluated the potential health impact of therapeutic HPV vaccines on cervical cancer prevention in China. Our modeling results indicate that therapeutic vaccines could substantially reduce cervical cancer cases and accelerate the timeline to elimination. The magnitude of impact varied depending on prophylactic vaccine coverage, screening strategies, and key characteristics of therapeutic vaccines. Notably, the infection-clearing vaccine (TxV1), when integrated into screening programs, averted the highest number of cervical cancer cases in the base case (PxV 2030, Screening status quo) —up to approximately 834.0 thousand, and accelarated the elimination timeline by 11 years. In the most pessimistic scenario (PxV status quo, Screening status quo), therapeutic vaccines could avert 335.1-1908.8 thousand cases across different Use Cases.

The health impact of therapeutic HPV vaccines is closely influenced by the broader context of cervical cancer prevention interventions. If China achieves the WHO 90–70–90 targets by 2030, the number of cervical cancer cases averted by therapeutic vaccines would decrease by 34.5%–87.0% compared with the status quo, consistent with other modelling studies showing diminishing incremental benefits as prophylactic vaccination and screening coverage increase [20, 27]. In particular, in the base case (PxV 2030, Screening status quo), our model indicates that the long-term health impact of therapeutic vaccines diminishes further, with only 0.1%–0.5% of cervical cancer cases being prevented after 2100, compared with reductions of 3.0-29.6% during 2051–2100. These findings highlight that therapeutic vaccines are likely to yield the greatest benefits during a “window of opportunity”—prior to the widespread scale-up of primary prevention programs, especially in the early stages of HPV vaccine introduction. In China, the HPV vaccine was only introduced into the national immunization program in late 2025 [5]. Prior to that, several provinces and cities had launched publicly funded or subsidized HPV vaccination initiatives, but the national HPV vaccine coverage among women was only 6.21% in 2022 [7].As a result, a large number of adult women are currently unvaccinated and are therefore still at risk of persistent HPV infection and cervical precancerous lesions. This interpretation was further supported by the age-stratified subgroup analysis, which showed that benefits were concentrated mainly among adult and older women: population-based TxV1 without catch-up mainly benefited women aged 35–54 years, whereas population-based TxV1 with catch-up and screening-integrated TxV1/TxV2 primarily benefited women aged ≥ 45 years. Therapeutic HPV vaccines may therefore serve as a supplementary intervention for adult women who have already passed the routine age for prophylactic vaccination.

Based on the WHO PPCs and the current development status of therapeutic HPV vaccines, our study modeled two distinct types of therapeutic vaccines [18]. Our results suggest that these vaccines may play complementary roles at different stages of cervical cancer control and under varying levels of health system capacity. For TxV1, population-based vaccination was associated with a 2.2%–13.0% reduction in cervical cancer incidence across scenarios, while screening-integrated TxV1 achieved higher impact, averting 12.7%–28.5% of cases. This increased effectiveness may be attributable to both the broader age coverage and the more precise identification of vaccine-type infections enabled by HPV-based screening. This greater impact was also accompanied by substantially greater dose efficiency: screening-integrated TxV1 required only 24 to 36 therapeutic vaccine doses per cervical cancer case averted, compared with approximately 1,077 to 6,677 doses for population-based TxV1. Accordingly, population-based TxV1 may be particularly beneficial in settings with high HPV prevalence but limited screening infrastructure, offering a feasible strategy to rapidly reduce the burden of vaccine-type HPV infections. In contrast, in regions with established HPV-based screening systems, integrating TxV1 into screening workflows may yield greater efficiency and health impact. Although our model incorporates spontaneous clearance of HPV infections, our findings may overestimate the efficiency of strategies involving TxV1. In practice, some women who would naturally clear their infection might still be identified as HPV-positive through screening and subsequently receive TxV1. While this does not affect the projected health impact in our model, it implies that TxV1 could be used in individuals who may not need treatment, reducing resource efficiency [28]. Future studies should assess the cost-effectiveness of TxV1 under targeted strategies that incorporate risk stratification tools such as HPV genotyping, viral load quantification, or biomarker-based triage, to better identify individuals with persistent infections who are most likely to benefit from vaccination.

For TxV2, this study evaluated only the screening-integrated delivery strategy. Similar to TxV1 in Use Case 2, TxV2 showed its highest impact under scenarios with low prophylactic vaccination coverage and high screening uptake—for example, under the PxV status quo with Screening 2030, where it reduced cervical cancer incidence by up to 7.4%. However, the overall magnitude of cancer reduction achieved by TxV2 was more limited compared to TxV1 across all scenarios. Sensitivity analyses further revealed that TxV2’s impact is highly dependent on the treatment rates for CIN2 and CIN3. When these rates were assumed to be at their lower bounds, the number of cervical cancer cases averted by TxV2 increased by 7.1% and 41.3%, respectively. Together with the rural-specific results, these findings underscore the role of TxV2 as a complementary intervention to existing excisional or ablative treatments, particularly in underserved settings where follow-up and treatment gaps remain [29, 30].The urban-rural stratified results further suggest that therapeutic HPV vaccination may support equity in cervical cancer prevention. Despite lower screening-related follow-up and treatment parameters in rural populations, the relative reductions in cervical cancer cases were broadly comparable between rural and urban areas in most scenarios, and slightly greater in rural areas in some scenarios. If future therapeutic vaccines can be delivered through simplified pathways, they may help reduce loss to follow-up after screening and partially narrow urban-rural gaps in access to diagnosis and treatment. In addition, as a non-invasive therapeutic option, TxV2 may offer fertility-sparing benefits and improve treatment acceptability among women of reproductive age. This suggests that lesion-targeted therapeutic vaccines could play a valuable role in expanding treatment choices and tailoring care for CIN patients, although further clinical validation is needed to confirm these benefits.

Although therapeutic HPV vaccines are not yet commercially available, modeling studies play a critical role in shaping vaccine development, informing policy planning, and forecasting public health impact under various implementation scenarios [18]. These analyses can help assess the public health value of target product characteristics, identify key drivers of population-level impact, and inform strategic investment and implementation planning before vaccine licensure. Importantly, our base-case assumptions should be interpreted as estimates of the potential population-level impact of future therapeutic HPV vaccines if they achieve preferred product characteristics. Future analyses should incorporate product-specific efficacy, durability, dose schedules, and delivery requirements as clinical evidence matures. Few modelling studies have evaluated the potential population-level impact of therapeutic HPV vaccines. One study projected potential effectiveness of a one-time therapeutic HPV vaccination campaign in Uganda [20], and a modelling report from the Daffodil Centre in Sydney examined the public health value of therapeutic vaccine in 78-LMIC [19]. Both studies concluded that the potential impact of therapeutic HPV vaccines was strongly influenced by the level of prophylactic vaccination coverage. Our findings reinforce this relationship, demonstrating similarly large differences in therapeutic vaccine impact across scenarios with varying primary prevention scale-up. However, previous analyses were primarily focused on low-and lower middle-income country settings. In contrast, China presents a distinct epidemiological and programmatic context, since it has a large population and was late to introduce population-wide HPV vaccination. Beyond evaluating large-scale population-based therapeutic vaccination strategies, our study examined the integration of therapeutic vaccines into China’s existing HPV-based screening programme, offering practical insights into vaccines operationalization within the current prevention infrastructure. Furthermore, by aligning analysis with the WHO cervical cancer elimination framework, we quantified the extent to which therapeutic vaccination could accelerate China’s progress toward elimination.

Our sensitivity analyses indicated that vaccine efficacy is a major determinant of population-level impact. Compared to a two-dose efficacy of 90%, reducing efficacy to 70% led to a 16.5–23.2% decline in cervical cancer cases averted, while lowering efficacy to 50% resulted in a 43.4–53.4% reduction in impact. Because the base-case efficacy assumptions were derived from WHO PPCs rather than licensed-product clinical evidence, they should be interpreted as an optimistic target-product-profile scenario. The reduced impact observed under lower-efficacy sensitivity analyses highlights that the real-world value of therapeutic HPV vaccines will depend strongly on the efficacy achieved by future products. Recent genotype-attribution evidence indicates that HPV16 and HPV18 remain the leading genotypes attributable to invasive cervical cancer globally, with HPV18 also remaining the second most common HPV type in invasive cervical cancer in China [31]. This supports our base-case HPV16/18-targeted vaccine assumption, while the high prevalence of HPV52 and HPV58 in China supports additional sensitivity analyses exploring broader genotype coverage [32, 33]. Our sensitivity analyses showed that including HPV52 and HPV58, alongside HPV16 and 18, could avert an additional 61.4–135.9% of cervical cancer cases. Importantly, cancers attributable to HPV types outside the vaccine target spectrum remained included in the model and contributed to the total age-standardised incidence used to assess elimination. Furthermore, the delayed introduction of prophylactic HPV vaccination in China also highlights the importance of timely policy adoption for future therapeutic vaccines. Large cohorts of adult women remain unvaccinated, creating a limited window during which therapeutic vaccination may provide substantial additional benefit. Our sensitivity analysis showed that delaying therapeutic vaccine roll-out from 2030 to 2035 reduced cervical cancer cases averted by 17.8%-37.5%, suggesting that slow adoption after licensure could diminish its public health value. Early planning for target populations, integration with screening pathways, and timely access will therefore be essential.

This study has several strengths. First, we employed a two-stage hybrid model to evaluate the potential health impact of therapeutic HPV vaccines. The first stage involved a dynamic transmission model, which allowed us to capture not only the direct effects of vaccination but also the potential herd protection conferred by therapeutic vaccines. Second, we incorporated comprehensive assumptions regarding key characteristics of therapeutic vaccines, including efficacy, coverage rate, genotype coverage, and year of introduction. We modeled two types of therapeutic vaccines with different mechanisms of action and evaluated two distinct delivery strategies, directly aligning with the scenarios outlined in the WHO PPCs for therapeutic HPV vaccines. In addition, we simulated multiple scenarios for prophylactic HPV vaccination and cervical cancer screening to reflect the range of cervical cancer prevention strategies that may be adopted in China in the coming decades, ensuring that the modeled implementation context for therapeutic vaccines closely approximates real-world conditions.

This study also has several limitations. First, the results are sensitive to key assumptions regarding therapeutic vaccine characteristics; therefore, the findings should be interpreted with caution. Rather than identifying a single optimal strategy, our goal was to explore a range of plausible scenarios and provide guidance for future research and policy planning. Second, our modeling assumptions did not account for potential durable immune memory, reduced reinfection risk, or reduced recurrence after therapeutic vaccination. Therapeutic vaccines were assumed to act only on existing HPV infections or CIN2/3 lesions at the time of vaccination. This conservative assumption may underestimate their long-term impact if future products are shown to confer sustained immune protection. We also did not capture potential reproductive health benefits, such as reductions in preterm births associated with decreased use of excisional treatment for CIN2/3. In addition, due to the absence of licensed therapeutic HPV vaccine products, we did not include price assumptions or conduct a cost-effectiveness analysis. We recognize this as an important area for future investigation. As clinical trial data and product-specific characteristics become available, we intend to incorporate more empirical and real-world parameters to further strengthen the robustness and applicability of our model outcomes.

## Conclusions

Our findings suggest that therapeutic HPV vaccines could play an important role in reducing cervical cancer incidence and accelerating elimination in China. As part of a comprehensive cervical cancer prevention strategy that includes prophylactic vaccination and HPV-based screening, therapeutic vaccines represent a promising tool to address persistent gaps and enhance health equity. These results provide timely and robust evidence to support China’s cervical cancer control efforts and inform broader global strategies aligned with the WHO elimination goals.

## Supplementary Information

Below is the link to the electronic supplementary material.

Supplementary Material 1: Additional File 1: Supplementary Materials. Tables S1–S19 and Figures S1–S12.

## Data Availability

The data generating the findings of this article are included within the article and its additional files.

## Abbreviations

CIN: Cervical intraepithelial neoplasia
HPV: Human papillomaviruses
LEEP: Loop electrosurgical excision procedure
LMICs: Low- and middle-income countries
NIP: National immunization program
PPC: Preferred Product Characteristics
WHO: World Health Organization
