# Supplementary material for: Potential health impact of therapeutic HPV vaccines in China: a modeling study
Source: BMC Med. 2026 Jul 7;24:539. doi: 10.1186/s12916-026-05050-0 (PMC13637135; doi:10.1186/s12916-026-05050-0)
Supplement: Supplementary file 1 [file 12916_2026_5050_MOESM1_ESM.docx]

**Potential health impact of therapeutic HPV vaccines in China: a modeling study**

**Supplementary Materials**

Chenghao Pan, Shangying Hu Tingting You, Meng Gao, Yanling Zhong, Xinyuan Wang, Youlin Qiao, Xuelian Zhao, Fanghui Zhao, Mark Jit

**Content**

[List of tables	2](#_Toc16929)

[List of figures	3](#_Toc6294)

[Methods	4](#_Toc13069)

[1.1 Model structure	4](#_Toc26242)

[1.2 Model calibration and validation	6](#_Toc4482)

[1.3 Model assumptions and inputs	12](#_Toc5780)

[1.3.1 Key parameters	12](#_Toc12957)

[1.3.2 Demographic and epidemiological data	13](#_Toc25718)

[1.4 Scenarios	14](#_Toc26108)

[1.5 Timeline of therapeutic vaccination	19](#_Toc24939)

[Checklists	23](#_Toc24457)

[Additional results	30](#_Toc13581)

[References	71](#_Toc12354)

# List of tables

**Table S1.** Model inputs

**Table S2.** Prophylactic HPV vaccine, screening and therapeitic HPV vaccine scenarios

**Table S3.** Checklist for HPV-FRAME core reporting standards

Table S4. Checklist for HPV-FRAME reporting standards for models of vaccination in adolescent individuals

Table S5. CHEERS 2022 Checklist

**Table S6.** Cervical cancer cases averted by therapeutic HPV vaccination and percentage reduction across different scenarios and age groups (thousands)

**Table S7.** Estimated cervical cancer cases across different scenarios in urban areas in China (thousands)

**Table S8.** Estimated cervical cancer cases across different scenarios in rural areas in China (thousands)

**Table S9.** Estimated cervical cancer cases averted by therapeutic vaccines across different scenarios in China (thousands)

**Table S10.** Estimated cervical cancer cases averted by therapeutic vaccines across different scenarios in urban and rural areas in China, 2025-2125 (thousands)

**Table S11.** Estimated cervical cancer deaths across different scenarios in China (thousands)

**Table S12.** Cervical cancer deaths averted by therapeutic HPV vaccination and percentage reduction across different scenarios and age groups (thousands)

**Table S13.** Estimated cervical cancer deaths across different scenarios in urban areas in China (thousands)

**Table S14.** Estimated cervical cancer deaths across different scenarios in rural areas in China (thousands)

**Table S15.** Estimated cervical cancer deaths averted by therapeutic vaccines across different scenarios in urban and rural areas in China (thousands)

**Table S16.** Estimated cervical cancer deaths averted by tharapeutic vaccines across different scenarios in urban and rural areas in China, 2025-2125 (thousands)

**Table S17.** Estimated cervical cancer cases and cases averted by tharapeutic vaccines for sensitivity analysis in China (thousands)

**Table S18.** Estimated cervical cancer deaths and deaths averted by tharapeutic vaccines for sensitivity analysis in China (thousands)

**Table S19.** Number of therapeutic vaccine doses needed to avert one cervical cancer case (2025-2100)

# List of figures

**Figure S1.** Two-stage hybrid model structure

**Figure S2.** Calibrated prevalence of HR-HPV

**Figure S3.** Calibrated HPV type distribution in various cervical conditions

**Figure S4.** Calibrated incidence and mortality of cervical cancer

**Figure S5.** Calibrated age-specific incidence of cervical cancer

**Figure S6.** Validation outputs from the 1000 Monte Carlo simulations

**Figure S7.** Rollout schedule of therapeutic HPV vaccination under Use Case 1-TxV1*

**Figure S8.** Rollout schedule of therapeutic HPV vaccination under Use Case 1-TxV1#

**Figure S9.** Rollout schedule of therapeutic HPV vaccination under Use Case 2-TxV1

**Figure S10.** Rollout schedule of therapeutic HPV vaccination under Use Case 2-TxV2

**Figure S11.** Sensitivity analysis of the effect of different assumptions about therapeutic vaccine characteristics on the percentage change in cervical cancer deaths averted compared with the base case

**Figure S12.** Sensitivity analysis of screening and treatment parameters influencing the cervical cancer deaths averted by TxV1 (A) and TxV2 (B) in Use Case 2, 2025–2125

# Methods

## 1.1 Model structure

In our previous work, we described our two-stage hybrid model combining a dynamic HPV transmission component and a natural history component^1^. To simulate the long-term health impact of therapeutic HPV vaccines in China, we extended the model to incorporate therapeutic HPV vaccines.

The previous publication describes this in detail; here we briefly summarise the main features. The dynamic transmission model simulated HPV transmission between males and females, incorporating sexual mixing patterns determined by partner acquisition rates and assortativity by age, area of residence (urban vs. rural), and sexual activity^2^ (categorized as none, low [one lifetime partner], or high [multiple partners]). This matched the stratification in the sexual behaviour survey we used to parameterize the model^2^. Thirteen high-risk HPV types (HPV16, 18, 31, 33, 35, 39, 45, 51, 52, 56, 58, 59, and 68) were modeled independently, allowing for type-specific differences in transmission probability and vaccine efficacy^1^. Infection-acquired natural immunity was assumed to confer temporary protection against reinfection with the same HPV type, with no cross-protection for other types, and waned over time. Girls vaccinated at predefined coverage levels acquired lifelong immunity according to vaccine efficacy assumptions.

The natural history model tracked the progression of HPV infection to cervical intraepithelial neoplasia (CIN1, CIN2, CIN3), invasive cervical cancer, and death. Individuals transitioned between health states: susceptible; immune (from infection or vaccination); infected; CIN1, CIN2, CIN3; effectively detected and treated; cervical cancer (preclinical or symptomatic); cured; and death (absorbing state). In the absence of screening, cervical cancer was diagnosed only upon symptom onset. Women with symptomatic cancer were subject to age-specific probabilities of death or cure. Those surviving five years after diagnosis were considered cured and removed from further cancer risk simulation.

Vaccination and screening interventions were embedded at relevant simulation stages. For prophylactic vaccination, girls aged 13 years were vaccinated at the specified coverage levels within the dynamic model. For cervical cancer screening, women aged 35–64 were screened every five years. Each woman’s probability of being screened in a given year was determined by the scenario-specific annual coverage, such that the average 5-year coverage matched empirical estimates and projected scale-up targets. Detection sensitivity was applied to HPV infection, CIN, and cancer. The average screening coverage within this age group varied by scenario and year, reflecting differences in program implementation and population uptake. Based on the “Action Plan for Accelerating the Elimination of Cervical Cancer (2022-2030)”^3^, we defined a screening scenario (HPV2030) in which screening coverage increases linearly to 70% by 2030. We also defined a more conservative scenario (HPV2050) to represent a slower scale-up of screening coverage.

The model population included all females alive or projected to be born in China between 2025 and 2125. This framework has previously been used to evaluate the cost-effectiveness of HPV vaccination and cervical cancer screening in China and to inform strategies for cervical cancer elimination.

**Figure S1.** Two-stage hybrid model structure

HPV=human papillomavirus. FOI=force of infection. CIN=cervical intraepithelial neoplasia. CC=cervical cancer.

## 1.2 Model calibration and validation

## 1.2.1 Calibration

The model was calibrated in three stages using epidemiological data on Chinese HPV prevalence, cervical cancer incidence and mortality in 2015, and HPV type distributions in various cervical conditions. The calibration assumed unchanged demographic characteristics. First, the annual HPV prevalence estimated by the dynamic model was calibrated to population-based epidemiological data. Second, results from calibrated dynamic model were fed into the natural history model to estimate the HPV type-specific cervical cancer incidence. Last, estimated cervical cancer mortality was calibrated to data from Chinese Cancer Registry. Calibration was performed using a least-squares (LS) approach to minimise the discrepancy between simulated outputs and calibration targets. We identified a subset of parameter sets that achieved good model fit, defined as those within the top 10% of the lowest residual sum of squares across all calibration targets. This subset of well-fitting parameter sets was used to characterise uncertainty arising from the calibration process. Parameter uncertainty was subsequently propagated through model projections by sampling from this retained parameter set in probabilistic sensitivity analyses.Model calibration outputs are shown in Figure S2-S5. Details of model calibration and validation have been reported in our previous study^1^.

**Figure S2.** Calibrated prevalence of HR-HPV

Models were calibrated using epidemiological data on HPV prevalence. HR-HPV=high risk human papillomavirus.

**Figure S3.** Calibrated HPV type distribution in various cervical conditions

Models were calibrated using epidemiological data of HPV type distributions in women with normal cervical cytology, cervical intraepithelial neoplasia, and invasive cervical cancer. CIN= cervical intraepithelial neoplasia.

**Figure S4.** Calibrated incidence and mortality of cervical cancer

Models were calibrated using epidemiological data of cervical cancer incidence and mortality. High sexual activity: individuals with multiple lifetime sexual partners. Low sexual activity: individuals with one lifetime sexual partner.

**Figure S5.** Calibrated age-specific incidence of cervical cancer

Models were calibrated using age-specific incidence of cervical cancer in China^4^. Shaded areas indicate the 95% uncertainty intervals of cervical cancer incidence calibration targets.

**1.2.2 Validation**

The model was validated by comparing model predictions with observed data from a population-based cohort study in China^5^. We ran 1 000 Monte Carlo simulations to sample parameter values from their distributions and estimate three indicators: 1) prevalent risk of CIN2+ after one positive HPV test, 2) five-year cumulative risk of CIN2+ after one positive HPV test, and 3) 10-year cumulative detection rate of CIN2+outcomes. The results demonstrated good agreement between simulated and observed data (Figure S5).

**Figure S6.** Validation outputs from the 1000 Monte Carlo simulations

Red points in vertical represent the 95% confidential interval reported in the population-based cohort study. Box plots indicate the distribution of results by running 1 000 Monte Carlo simulations

## 1.3 Model assumptions and inputs

## 1.3.1 Key parameters

Key parameters relevant to this analysis are described in table S1. The sources and selection of these parameters have been detailed in our previous study, in which the model was calibrated and validated^1^. The range of values tested in the sensitivity analysis for each parameter was either based on the reported 95% confidence intervals in the referenced studies or determined by assuming a 25% change from the base-case value. And probabilistic sensitivity analysis was conducted by performing 500 Monte Carlo simulations to sample parameter values from their distributions and estimate outcomes.

**Table S1. Model inputs**

| **Parameter** | **Base case** | **Range** | **Distribution** | **Source** |  |
| --- | --- | --- | --- | --- | --- |
| **Probability of HPV clearance** | |  |  |  | |
| 12-24 years | 0.7188 | 0.6463-0.7830 | Beta | ^6-9^ |  |
| 25-29 years | 0.6984 | 0.5898-0.7952 | Beta | ^6-9^ |  |
| 30-39 years | 0.3503 | 0.2860-0.4188 | Beta | ^6-9^ |  |
| 40-49 years | 0.2048 | 0.1118-0.3022 | Beta | ^6-10^ |  |
| >50 years | 0.1004 | 0.0546-0.1567 | Beta | ^6-9^ |  |
| **Probability of progression and regression** | |  |  |  | |
| Infection to CIN1 | 0.075 | 0.056-0.1154 | Beta | ^6,11^ |  |
| CIN1 to CIN2 | 0.2240 | 0.1608-0.2972 | Beta | ^6-9^ |  |
| CIN2 to CIN3 | 0.3498 | 0.0904-0.8654 | Beta | ^6-9^ |  |
| CIN3 to CC Ⅰ | 0.1019 | 0.0189-0.2906 | Beta | ^6-9^ |  |
| CIN2 to CIN1 | 0.2494 | 0.1994-0.2992 | Beta | ^6-9^ |  |
| CIN3 to CIN2 | 0.0135 | ±25% | Beta | ^6^ |  |
| CIN2 to infection/immunity | 0.1901 | ±25% | Beta | ^6,12^ |  |
| CIN1 to CIN3 | 0.0464 | 0.0098-0.1297 | Beta | ^6-9^ |  |
| CC Ⅰ to CC Ⅱ | 0.4376587 | ±25% | Beta | ^11,13^ |  |
| CC Ⅱ to CC Ⅲ | 0.5358411 | ±25% | Beta | ^11,13^ |  |
| CC Ⅲ to CC Ⅳ | 0.6837722 | ±25% | Beta | ^11,13^ |  |
| Waning of natural immunity | 0.021 | 0.015-0.027 | Beta | ^[9]^ |  |
| **Cancer symptoms** |  |  |  |  |  |
| CC Ⅰ | 0.15 | ±25% | Beta | ^11,13^ |  |
| CC Ⅱ | 0.225 | ±25% | Beta | ^11,13^ |  |
| CC Ⅲ | 0.6 | ±25% | Beta | ^11,13^ |  |
| CC Ⅳ | 0.9 | ±25% | Beta | ^11,13^ |  |
| **Vaccine efficacy** |  |  |  |  |  |
| Target types | 1 | 0.8-1.0 | Binomial | Assumed^14-16^ |  |
| **Screening sensitivity** |  |  |  |  |  |
| **Liquid-based cytology** |  |  |  |  |  |
| CIN1 | 0.47 | ±25% | Beta | ^17^ |  |
| CIN2/3 | 0.53 | 0.486-0.574 | Beta | ^17^ |  |
| CC Ⅰ | 0.90 | ±25% | Beta | Assumed |  |
| CC Ⅱ | 0.92 | ±25% | Beta | Assumed |  |
| CC Ⅲ | 0.95 | ±25% | Beta | Assumed |  |
| CC Ⅳ | 0.99 | ±25% | Beta | Assumed |  |
| **HPV test** |  |  |  |  |  |
| CIN1 | 0.80 | ±25% | Beta | Assumed |  |
| CIN2 | 0.90 | ±25% | Beta | Assumed^16^ |  |
| CIN3 | 0.94 | ±25% | Beta | Assumed^16^ |  |
| CC Ⅰ-Ⅳ | 1.00 | .. | .. | Assumed |  |
| **Precancerous lesion management** | |  |  |  | |
| **Urban** |  |  |  |  |  |
| Follow-up of CIN1 | 0.4631 | 0.4168-0.5094 | Beta | ^1^ |  |
| Treatment of CIN1 | 0.4421 | 0.3979-0.4863 | Beta | ^1^ |  |
| Treatment of CIN2/3 | 0.9533 | 0.8580-1.0000 | Beta | ^1^ |  |
| **Rural** |  |  |  |  |  |
| Follow-up of CIN1 | 0.3871 | 0.3484-0.4258 | Beta | ^1^ |  |
| Treatment of CIN1 | 0.4535 | 0.4082-0.4989 | Beta | ^1^ |  |
| Treatment of CIN2/3 | 0.8946 | 0.8051-0.9841 | Beta | ^1^ |  |
| **Treatment efficacy** |  |  |  |  |  |
| **Urban** |  |  |  |  |  |
| CIN1 treatment efficacy | 1.0000 | 0.9956-1.0000 | .. | ^1^ |  |
| CIN2/3 treatment efficacy | 0.9367 | 0.9278-0.9455 | Beta | ^1^ |  |
| **Rural** |  |  |  |  |  |
| CIN1 treatment efficacy | 0.9978 | 0.9943-1.0000 | Beta | ^1^ |  |
| CIN2/3 treatment efficacy | 0.9000 | 0.8846-0.9154 | Beta | ^1^ |  |
| **Achievable coverage of vaccination** | 0.90 | 0.70-0.95 | Binomial | Assumed^18^ |  |
| **Achievable coverage of screening** | 0.90 | 0.70-0.90 | Binomial | ^18,19^ |  |

Abbreviations: CIN, cervical intraepithelial neoplasia; CC, cervical cancer; LBC, liquid-based cytology; HPV, Human Papillomavirus.

Urban population refers to all people residing in urban areas, while rural population refers to people residing in rural areas.

## 1.3.2 Demographic and epidemiological data

Demographic and epidemiological data were obtained from open-source publications or government-released online datasets^20-27^ and were reported in the previous study.^1,28^ Population size in 2015 was obtained from National Bureau of Statistics of China.^20,21^ The all-cause mortality rate was obtained from National Health Commission of China.^22^ Age-specific incidence and mortality of cervical cancer in 2015 were obtained from Chinese Cancer Registry Report released by National Cancer Center of China.^23^ In addition, the data sources and analytical methods of HPV prevalence, fertility rate, sexual activity, and screening coverage were reported in the previous study.^1^ The projected fertility rate of women aged 15-49 years^24^ and urbanisation data^25,26^ were from the UN Population Division. The patterns in sexual activity in China were projected with historical data from the USA as a reference and were adjusted for the Human Development Index.^27^

## 1.4 Scenarios

We considered different levels of prophylactic HPV vaccination coverage and cervical cancer screening uptake as background conditions for therapeutic HPV vaccine implementation. In brief, these background scenarios included maintaining the current situation (status quo), achieving 90% coverage of prophylactic HPV vaccination among eligible girls by 2030 or 2050 (PxV2030 and PxV2050), and achieving 70% coverage of HPV-based cervical cancer screening among women aged 35–64 years by 2030 or 2050 (HPV2030 and HPV2050). By combining these assumptions, we generated seven main analytical scenarios: (1) both vaccination and screening maintained at the status quo; (2) vaccination at the status quo with screening reaching 70% coverage by 2030 (PxV status quo, HPV2030); (3) vaccination at the status quo with screening reaching 70% coverage by 2050 (PxV status quo, HPV2050); (4) vaccination reaching 90% coverage by 2030 with screening at the status quo (PxV2030, HPV status quo); (5) vaccination reaching 90% coverage by 2050 with screening at the status quo (PxV2050, HPV status quo); (6) both vaccination and screening reaching their targets by 2030 (PxV2030, HPV2030); and (7) both reaching their targets by 2050 (PxV2050, HPV2050) (Table S2).

In the sensitivity analysis, we varied the characteristics of the therapeutic HPV vaccines under the status quo background scenario to assess their influence on the overall health impact of therapeutic vaccination (Table S2)

**Table S2. Prophylactic HPV vaccine, screening and therapeitic HPV vaccine scenarios**

| **Scenario** | **PxV**  **scenario** | **Screening scenario** | **Use case** | **Type of TxV** | **Vaccine efficacy of 2 doses / 1 dose of TxV in preventing HPV infections or precancerous lesions** | **Coverage of TxV**  **(at least 1 dose/ 2 doses)** | **Roll-out year of TxV** | **Targeted HPV types of TxV** | **Comparator**  **(averted numbers)** |
| --- | --- | --- | --- | --- | --- | --- | --- | --- | --- |
| 1a | PxV 2030 | Status quo | 0 | TxV 0 | - | - | - | - | - |
| 1b | PxV 2030 | Status quo | 1 | TxV 1^*^ | 90%/70% | 90%/70% | 2030 | 16,18 | 1a |
| 1c | PxV 2030 | Status quo | 1 | TxV 1^#^ | 90%/70% | 90%/70% | 2030 | 16,18 | 1a |
| 1d | PxV 2030 | Status quo | 2 | TxV 1 | 90%/70% | 90%/70% | 2030 | 16,18 | 1a |
| 1e | PxV 2030 | Status quo | 2 | TxV 2 | 90%/70% | 90%/70% | 2030 | 16,18 | 1a |
| 2a | PxV 2050 | Status quo | 0 | TxV 0 | - | - | - | - | - |
| 2b | PxV 2050 | Status quo | 1 | TxV 1^*^ | 90%/70% | 90%/70% | 2030 | 16,18 | 2a |
| 2c | PxV 2050 | Status quo | 1 | TxV 1^#^ | 90%/70% | 90%/70% | 2030 | 16,18 | 2a |
| 2d | PxV 2050 | Status quo | 2 | TxV 1 | 90%/70% | 90%/70% | 2030 | 16,18 | 2a |
| 2e | PxV 2050 | Status quo | 2 | TxV 2 | 90%/70% | 90%/70% | 2030 | 16,18 | 2a |
| 3a | PxV 2030 | HPV 2030 | 0 | TxV 0 | - | - | - | - | - |
| 3b | PxV 2030 | HPV 2030 | 1 | TxV 1^*^ | 90%/70% | 90%/70% | 2030 | 16,18 | 3a |
| 3c | PxV 2030 | HPV 2030 | 1 | TxV 1^#^ | 90%/70% | 90%/70% | 2030 | 16,18 | 3a |
| 3d | PxV 2030 | HPV 2030 | 2 | TxV 1 | 90%/70% | 90%/70% | 2030 | 16,18 | 3a |
| 3e | PxV 2030 | HPV 2030 | 2 | TxV 2 | 90%/70% | 90%/70% | 2030 | 16,18 | 3a |
| 4a | PxV 2050 | HPV 2050 | 0 | TxV 0 | - | - | - | - | - |
| 4b | PxV 2050 | HPV 2050 | 1 | TxV 1^*^ | 90%/70% | 90%/70% | 2030 | 16,18 | 4a |
| 4c | PxV 2050 | HPV 2050 | 1 | TxV 1^#^ | 90%/70% | 90%/70% | 2030 | 16,18 | 4a |
| 4d | PxV 2050 | HPV 2050 | 2 | TxV 1 | 90%/70% | 90%/70% | 2030 | 16,18 | 4a |
| 4e | PxV 2050 | HPV 2050 | 2 | TxV 2 | 90%/70% | 90%/70% | 2030 | 16,18 | 4a |
| 5a | Status quo | Status quo | 0 | TxV 0 | - | - | - | - | - |
| 5b | Status quo | Status quo | 1 | TxV 1^*^ | 90%/70% | 90%/70% | 2030 | 16,18 | 5a |
| 5c | Status quo | Status quo | 1 | TxV 1^#^ | 90%/70% | 90%/70% | 2030 | 16,18 | 5a |
| 5d | Status quo | Status quo | 2 | TxV 1 | 90%/70% | 90%/70% | 2030 | 16,18 | 5a |
| 5e | Status quo | Status quo | 2 | TxV 2 | 90%/70% | 90%/70% | 2030 | 16,18 | 5a |
| 6a | Status quo | HPV 2030 | 0 | TxV 0 | - | - | - | - | - |
| 6b | Status quo | HPV 2030 | 1 | TxV 1^*^ | 90%/70% | 90%/70% | 2030 | 16,18 | 6a |
| 6c | Status quo | HPV 2030 | 1 | TxV 1^#^ | 90%/70% | 90%/70% | 2030 | 16,18 | 6a |
| 6d | Status quo | HPV 2030 | 2 | TxV 1 | 90%/70% | 90%/70% | 2030 | 16,18 | 6a |
| 6e | Status quo | HPV 2030 | 2 | TxV 2 | 90%/70% | 90%/70% | 2030 | 16,18 | 6a |
| 7a | Status quo | HPV 2050 | 0 | TxV 0 | - | - | - | - | - |
| 7b | Status quo | HPV 2050 | 1 | TxV 1^*^ | 90%/70% | 90%/70% | 2030 | 16,18 | 7a |
| 7c | Status quo | HPV 2050 | 1 | TxV 1^#^ | 90%/70% | 90%/70% | 2030 | 16,18 | 7a |
| 7d | Status quo | HPV 2050 | 2 | TxV 1 | 90%/70% | 90%/70% | 2030 | 16,18 | 7a |
| 7e | Status quo | HPV 2050 | 2 | TxV 2 | 90%/70% | 90%/70% | 2030 | 16,18 | 7a |
| S1a | PxV 2030 | Status quo | 1 | TxV 1^*^ | 70%/50% | 90%/70% | 2030 | 16,18 | 1a |
| S1b | PxV 2030 | Status quo | 1 | TxV 1^#^ | 70%/50% | 90%/70% | 2030 | 16,18 | 1a |
| S1c | PxV 2030 | Status quo | 2 | TxV 1 | 70%/50% | 90%/70% | 2030 | 16,18 | 1a |
| S1d | PxV 2030 | Status quo | 2 | TxV 2 | 70%/50% | 90%/70% | 2030 | 16,18 | 1a |
| S1e | PxV 2030 | Status quo | 1 | TxV 1^*^ | 50%/0% | 90%/70% | 2030 | 16,18 | 1a |
| S1f | PxV 2030 | Status quo | 1 | TxV 1^#^ | 50%/0% | 90%/70% | 2030 | 16,18 | 1a |
| S1g | PxV 2030 | Status quo | 2 | TxV 1 | 50%/0% | 90%/70% | 2030 | 16,18 | 1a |
| S1h | PxV 2030 | Status quo | 2 | TxV 2 | 50%/0% | 90%/70% | 2030 | 16,18 | 1a |
| S2a | PxV 2030 | Status quo | 1 | TxV 1^*^ | 90%/70% | 50%/50% | 2030 | 16,18 | 1a |
| S2b | PxV 2030 | Status quo | 1 | TxV 1^#^ | 90%/70% | 50%/50% | 2030 | 16,18 | 1a |
| S2c | PxV 2030 | Status quo | 2 | TxV 1 | 90%/70% | 50%/50% | 2030 | 16,18 | 1a |
| S2d | PxV 2030 | Status quo | 2 | TxV 2 | 90%/70% | 50%/50% | 2030 | 16,18 | 1a |
| S2e | PxV 2030 | Status quo | 1 | TxV 1^*^ | 90%/70% | 100%/100% | 2030 | 16,18 | 1a |
| S2f | PxV 2030 | Status quo | 1 | TxV 1^#^ | 90%/70% | 100%/100% | 2030 | 16,18 | 1a |
| S2g | PxV 2030 | Status quo | 2 | TxV 1 | 90%/70% | 100%/100% | 2030 | 16,18 | 1a |
| S2h | PxV 2030 | Status quo | 2 | TxV 2 | 90%/70% | 100%/100% | 2030 | 16,18 | 1a |
| S3a | PxV 2030 | Status quo | 1 | TxV 1^*^ | 90%/70% | 90%/70% | 2027 | 16,18 | 1a |
| S3b | PxV 2030 | Status quo | 1 | TxV 1^#^ | 90%/70% | 90%/70% | 2027 | 16,18 | 1a |
| S3c | PxV 2030 | Status quo | 2 | TxV 1 | 90%/70% | 90%/70% | 2027 | 16,18 | 1a |
| S3d | PxV 2030 | Status quo | 2 | TxV 2 | 90%/70% | 90%/70% | 2027 | 16,18 | 1a |
| S3e | PxV 2030 | Status quo | 1 | TxV 1^*^ | 90%/70% | 90%/70% | 2035 | 16,18 | 1a |
| S3f | PxV 2030 | Status quo | 1 | TxV 1^#^ | 90%/70% | 90%/70% | 2035 | 16,18 | 1a |
| S3g | PxV 2030 | Status quo | 2 | TxV 1 | 90%/70% | 90%/70% | 2035 | 16,18 | 1a |
| S3h | PxV 2030 | Status quo | 2 | TxV 2 | 90%/70% | 90%/70% | 2035 | 16,18 | 1a |
| S4a | PxV 2030 | Status quo | 1 | TxV 1^*^ | 90%/70% | 90%/70% | 2030 | 16,18,52,58 | 1a |
| S4b | PxV 2030 | Status quo | 1 | TxV 1^#^ | 90%/70% | 90%/70% | 2030 | 16,18,52,58 | 1a |
| S4c | PxV 2030 | Status quo | 2 | TxV 1 | 90%/70% | 90%/70% | 2030 | 16,18,52,58 | 1a |
| S4d | PxV 2030 | Status quo | 2 | TxV 2 | 90%/70% | 90%/70% | 2030 | 16,18,52,58 | 1a |
| S4e | PxV 2030 | Status quo | 1 | TxV 1^*^ | 90%/70% | 90%/70% | 2030 | 16,18,52,58,31,33,35,45 | 1a |
| S4f | PxV 2030 | Status quo | 1 | TxV 1^#^ | 90%/70% | 90%/70% | 2030 | 16,18,52,58,31,33,35,45 | 1a |
| S4g | PxV 2030 | Status quo | 2 | TxV 1 | 90%/70% | 90%/70% | 2030 | 16,18,52,58,31,33,35,45 | 1a |
| S4h | PxV 2030 | Status quo | 2 | TxV 2 | 90%/70% | 90%/70% | 2030 | 16,18,52,58,31,33,35,45 | 1a |

Base case = two-dose prophylactic HPV vaccine coverage among 13-year-old girls increases linearly to 90% by 2030, while screening coverage remains unchanged.

Status quo = current prophylactic HPV vaccination or/and screening&treatment practices; PxV 2030/2050 = two-dose prophylactic HPV vaccine coverage among 13-year-old girls increases linearly to 90% by 2030/2050; Screening 2030/2050 = HPV DNA-based screening coverage increases linearly to 90% by 2030/2050; TxV0 = no therapeutic vaccination; Use Case 1 (TxV1*) = population-based vaccination with a type 1 therapeutic vaccine targeting clearance of oncogenic HPV infections, administered annually to women aged 30 years; Use Case 1 (TxV1#) = same as TxV1*, with an initial catch-up campaign for women aged 31-45 years in the first year of roll-out; Use Case 2 (TxV1) = screening-based vaccination with a type 1 therapeutic vaccine targeting HPV-positive, triage-negative women; Use Case 2 (TxV2) = screening-based vaccination with a type 2 therapeutic vaccine targeting women diagnosed with CIN2/3 through HPV-based screening.

## 1.5 Timeline of therapeutic vaccination

Two implementation Use Cases were considered. In Use Case 1, a population-based delivery strategy for therapeutic vaccination was modelled, with rollout beginning in 2030. Two delivery options were evaluated. In Use Case 1-TxV1*, women aged 30 years received a two-dose therapeutic vaccine targeting clearance of HPV16/18 infections (Figure S7). In Use Case 1-TxV1#, this strategy was supplemented with a one-time catch-up campaign in the first year of rollout, targeting women aged 31-45 years (Figure S8) .

**Figure S7.** Rollout schedule of therapeutic HPV vaccination under Use Case 1-TxV1*

**Figure S8.** Rollout schedule of therapeutic HPV vaccination under Use Case 1-TxV1#

In Use Case 2, therapeutic vaccination was integrated into HPV-based cervical cancer screening programmes, with rollout beginning in 2030. In China, the national cervical cancer screening programme targets women aged 35–64 years. Two screening-based delivery strategies were modelled. In Use Case 2–TxV1, women who tested positive for HPV but had a negative triage result received a two-dose therapeutic vaccine targeting clearance of HPV16/18 infections (Figure S9). In Use Case 2–TxV2, women diagnosed with cervical intraepithelial neoplasia grade 2 or 3 (CIN2/3) following a positive HPV test and histopathological confirmation received a two-dose therapeutic vaccine targeting precancerous lesions (Figure S10).

**Figure S9.** Rollout schedule of therapeutic HPV vaccination under Use Case 2-TxV1

**Figure S10.** Rollout schedule of therapeutic HPV vaccination under Use Case 2-TxV2

# Checklists

**Table S3.** Checklist for HPV-FRAME core reporting standards

| **Inputs** | **Reported by age? (Y/N)** | **Reported by sex? (B/F/M/N)** | **Comments** |
| --- | --- | --- | --- |
| Target population for intervention | Y | F | Girls aged 14 years |
| Sexual behavior | Y | B | Sexual mixing was modelled based on partner acquisition rates and assortativity by age, area (urban and rural), and sexual activity (none, low [individuals with one lifetime sexual partner], and high [individuals with multiple lifetime sexual partners]). Partner acquisition rates were adjusted to maintain the number of male-female sexual partnerships. The Gaussian kernel was used to model age assortativity. |
| Cohort examined for evaluation/time horizon | Y (implicitly) | F | We evaluated outcomes in the whole female population over a 100-year time horizon |
| Quality of life assumptions | N | N | N |
| Calibration | Y | F | Reported in the previous articles^1,28^ |
| Validation | Y | F | Reported in the previous articles^1,28^ |
| Costs | N | N | N |
| **Outputs** | **Reported by age? (Y/N)** | **Reported by sex? (B/F/M/N)** | **Report as calibration or validation target? (Y/N)** |
| Cancer incidence, mortality, life years, QALYs/DALYs | Y (implicitly) | F | N (Only reported as outcomes in this study; age-standardized/age-specific cervical cancer incidence and/or mortality were reported as calibration outputs in our previous work) |
| HPV prevalence, pre-intervention | N | N | N (age-standardized/age-specific HR-HPV prevalence was reported as calibration outputs in our previous work) |
| CIN2 detected | N | N | N |
| Sensitivity analysis of key inputs | N | F | Probabilistic sensitivity analysis was conducted by performing 500 Monte Carlo simulations to sample parameter values from their distributions and estimate outcomes.  Scenario sensitivity analysis was also considered in this study. |
| Incremental cost-effectiveness ratios and costs saved | N | N | N |

Y: yes; N: no; B: both sexes; F: female-only; M: male-only

Table S4 Checklist for HPV-FRAME reporting standards for models of vaccination in adolescent individuals

| **Inputs** | **Reported? (Y/N)** | **Reported by age? (Y/N)** | **Reported by sex? (B/F/M/N)** | **Comments** |
| --- | --- | --- | --- | --- |
| Vaccine uptake | Y | Y | F | Coverage for routine vaccination of girls aged 14 was assumed to be 90%. Vaccination in males was not considered in this study. |
| Vaccine efficacy | Y | N | F | Two-dose schedule: 100% lifelong protection against vaccine-targeted HPV types (independent of sex). |
| Vaccine cross-protection | Y | N | F | Domestically produced HPV-2 was assumed to provide no cross-protection for HPV types not included as vaccine antigens due to the absence of evidence (independent of sex and age). |
| Vaccine duration and warning | Y | Y (implicitly) | F | Vaccine-acquired immunity was assumed to be lifelong (independent of sex and age). |
| Vaccine and delivery costs | N | N | N |  |
| Pre-vaccination disease burden | Y | Y | F | Reported in the previous articles^1,28^ |
| Heterogeneity in sexual behavior | Y | Y | B | Sexual activity: none, low (individuals with one lifetime sexual partner), and high (individuals with multiple lifetime sexual partners). |
| Duration of natural immunity | Y | Y (implicitly) | F | Reported as the parameter for waning of natural immunity (independent of sex and age). |
| **Outputs** |  |  |  |  |
| Absolute reductions in HPV infections, and/or warts, post-vaccination | N | N | N |  |
| Absolute reductions in CIN2+ post-vaccination | N | N | N |  |
| Absolute reductions in invasive cancer post-vaccination | Y | Y | F | Reported in the appendix Table S8-9, Table S13-14, |

Y: yes; N: no; B: both sexes; F: female-only; M: male-only

Table S5 CHEERS 2022 Checklist

| **Topic** | **No.** | **Item** | **Location where item is reported** |
| --- | --- | --- | --- |
| **Title** | 1 | Identify the study as an economic evaluation and specify the interventions being compared. | Page1, title |
| **Abstract** | 2 | Provide a structured summary that highlights context, key methods, results, and alternative analyses. | Page4, abstract |
| **Introduction** |  |  |  |
| **Background and objectives** | 3 | Give the context for the study, the study question, and its practical relevance for decision making in policy or practice. | Page 5-6 |
| **Methods** |  |  |  |
| **Health economic analysis plan** | 4 | Indicate whether a health economic analysis plan was developed and where available. | Not Reported |
| **Study population** | 5 | Describe characteristics of the study population (such as age range, demographics, socioeconomic, or clinical characteristics). | Page 7, method section, line 126-129 |
| **Setting and location** | 6 | Provide relevant contextual information that may influence findings. | Page 7, Vaccination and screening scenarios, inputs and assumption; appendix Page12-13 |
| **Comparators** | 7 | Describe the interventions or strategies being compared and why chosen. | Page 9, method section, line 177-178;  appendix Page 15-17 |
| **Perspective** | 8 | State the perspective(s) adopted by the study and why chosen. | N/A |
| **Time horizon** | 9 | State the time horizon for the study and why appropriate. | Page 9, line 175-176 |
| **Discount rate** | 10 | Report the discount rate(s) and reason chosen. | N/A |
| **Selection of outcomes** | 11 | Describe what outcomes were used as the measure(s) of benefit(s) and harm(s). | Page 8, Outcomes and analysis |
| **Measurement of outcomes** | 12 | Describe how outcomes used to capture benefit(s) and harm(s) were measured. | Page 8, Outcomes and analysis |
| **Valuation of outcomes** | 13 | Describe the population and methods used to measure and value outcomes. | Page 8, Outcomes and analysis |
| **Measurement and valuation of resources and costs** | 14 | Describe how costs were valued. | N/A |
| **Currency, price date, and conversion** | 15 | Report the dates of the estimated resource quantities and unit costs, plus the currency and year of conversion. | N/A |
| **Rationale and description of model** | 16 | If modelling is used, describe in detail and why used. Report if the model is publicly available and where it can be accessed. | Page 6, Overview; appendix 1.1 Model structure; 1.2 Model calibration and validation |
| **Analytics and assumptions** | 17 | Describe any methods for analysing or statistically transforming data, any extrapolation methods, and approaches for validating any model used. | appendix 1.1 Model structure; 1.2 Model calibration and validation and previous work^1,28^ |
| **Characterising heterogeneity** | 18 | Describe any methods used for estimating how the results of the study vary for subgroups. | Page 6-7, Therapeutic vaccine assumptions and vaccination strategies ; appendix table S2 |
| **Characterising distributional effects** | 19 | Describe how impacts are distributed across different individuals or adjustments made to reflect priority populations. | N/A |
| **Characterising uncertainty** | 20 | Describe methods to characterise any sources of uncertainty in the analysis. | Page 9, Outcomes and analysis; appendix 1.2 Model calibration and validation |
| **Approach to engagement with patients and others affected by the study** | 21 | Describe any approaches to engage patients or service recipients, the general public, communities, or stakeholders (such as clinicians or payers) in the design of the study. | N/A |
| **Results** |  |  |  |
| **Study parameters** | 22 | Report all analytic inputs (such as values, ranges, references) including uncertainty or distributional assumptions. | Appendix table S1 |
| **Summary of main results** | 23 | Report the mean values for the main categories of costs and outcomes of interest and summarise them in the most appropriate overall measure. | Page 8-10, Result; Page 22-32, Tables and figures |
| **Effect of uncertainty** | 24 | Describe how uncertainty about analytic judgments, inputs, or projections affect findings. Report the effect of choice of discount rate and time horizon, if applicable. | Page 22-32,Tables and figures |
| **Effect of engagement with patients and others affected by the study** | 25 | Report on any difference patient/service recipient, general public, community, or stakeholder involvement made to the approach or findings of the study | N/A |
| **Discussion** |  |  |  |
| **Study findings, limitations, generalisability, and current knowledge** | 26 | Report key findings, limitations, ethical or equity considerations not captured, and how these could affect patients, policy, or practice. | Page 10-15 |
| **Other relevant information** |  |  |  |
| **Source of funding** | 27 | Describe how the study was funded and any role of the funder in the identification, design, conduct, and reporting of the analysis | Page 4, Abstract ; Page 10, Role of the funding source; Page 16, Acknowledgement |
| **Conflicts of interest** | 28 | Report authors conflicts of interest according to journal or International Committee of Medical Journal Editors requirements. | Page 16, Declaration of interest |

# Additional results

**Table S6.** Cervical cancer cases averted by therapeutic HPV vaccination and percentage reduction across different scenarios and age groups (thousands)

| **Scenario** | **Use case** | **Type of TxV** | **Cases of cervical cancer averted by TxV and percentage reduction** | | | | |
| --- | --- | --- | --- | --- | --- | --- | --- |
|  |  |  | **0-34 years** | **35-44 years** | **45-54 years** | **55-64 years** | **65+ years** |
| **Base case (PxV 2030, Screening status quo)** | | |  |  |  |  |  |
|  | 1 | TxV 1^*^ | 3.9 (2.4%) | 54.9 (34.2%) | 55.3 (34.5%) | 26.4 (16.4%) | 20.1 (12.5%) |
|  | 1 | TxV 1^#^ | 4.4 (1%) | 79.7 (18.4%) | 153.8 (35.5%) | 101.5 (23.5%) | 93.3 (21.6%) |
|  | 2 | TxV 1 | 0.9 (0.1%) | 43.3 (6.2%) | 168.8 (24.1%) | 196.2 (28%) | 291.1 (41.6%) |
|  | 2 | TxV 2 | 1.8 (1.2%) | 17.9 (12.1%) | 49.6 (33.5%) | 43.9 (29.7%) | 34.8 (23.5%) |
| **PxV 2050,Screening status quo** | | |  |  |  |  |  |
|  | 1 | TxV 1^*^ | 7 (2.7%) | 81 (31.1%) | 89 (34.2%) | 45.8 (17.6%) | 37.5 (14.4%) |
|  | 1 | TxV 1^#^ | 7.9 (1.5%) | 106.5 (19.8%) | 189.2 (35.1%) | 122.5 (22.7%) | 112.6 (20.9%) |
|  | 2 | TxV 1 | 2 (0.2%) | 60.3 (7%) | 217.6 (25.2%) | 239.7 (27.8%) | 343.5 (39.8%) |
|  | 2 | TxV 2 | 2.4 (1.4%) | 22 (12.9%) | 58 (33.9%) | 50 (29.2%) | 38.5 (22.5%) |
| **PxV 2030,Screening 2030** | | |  |  |  |  |  |
|  | 1 | TxV 1^*^ | 3.9 (3.9%) | 35.5 (35.8%) | 34.6 (34.8%) | 12.1 (12.2%) | 13.2 (13.3%) |
|  | 1 | TxV 1^#^ | 4.4 (1.5%) | 53.8 (18.8%) | 108.9 (37.9%) | 54.3 (18.9%) | 65.5 (22.8%) |
|  | 2 | TxV 1 | 1 (0.2%) | 43.6 (6.4%) | 154.7 (22.7%) | 154.1 (22.6%) | 328.4 (48.2%) |
|  | 2 | TxV 2 | 1.8 (0.9%) | 22.3 (10.8%) | 71.2 (34.6%) | 69.7 (33.9%) | 40.7 (19.8%) |
| **PxV 2050,Screening 2050** | | |  |  |  |  |  |
|  | 1 | TxV 1^*^ | 7 (3.7%) | 61.4 (32.7%) | 68.9 (36.7%) | 24.9 (13.3%) | 25.7 (13.7%) |
|  | 1 | TxV 1^#^ | 7.9 (1.8%) | 83.6 (19.5%) | 165.2 (38.6%) | 86.3 (20.1%) | 85.6 (20%) |
|  | 2 | TxV 1 | 2.1 (0.3%) | 58.7 (7.5%) | 198.6 (25.2%) | 186.7 (23.7%) | 340.5 (43.3%) |
|  | 2 | TxV 2 | 2.4 (1.1%) | 23.1 (11%) | 71.8 (34.2%) | 70.8 (33.7%) | 42.1 (20%) |
| **PxV status quo, Screening status quo** | | |  |  |  |  |  |
|  | 1 | TxV 1^*^ | 40.5 (5%) | 279.3 (34.7%) | 279.2 (34.7%) | 123.9 (15.4%) | 81 (10.1%) |
|  | 1 | TxV 1^#^ | 42.3 (3.9%) | 305.8 (28.1%) | 381 (35%) | 201.7 (18.5%) | 157.2 (14.4%) |
|  | 2 | TxV 1 | 14.5 (0.9%) | 181.8 (10.7%) | 512.3 (30.1%) | 457.5 (26.9%) | 534.5 (31.4%) |
|  | 2 | TxV 2 | 8 (2.5%) | 55.7 (17.6%) | 115.4 (36.5%) | 83.4 (26.4%) | 53.9 (17%) |
| **PxV status quo,Screening 2030** | | |  |  |  |  |  |
|  | 1 | TxV 1^*^ | 40.5 (8.1%) | 176.8 (35.4%) | 172.9 (34.6%) | 57.4 (11.5%) | 51.7 (10.4%) |
|  | 1 | TxV 1^#^ | 42.3 (6.1%) | 196.3 (28.3%) | 249.2 (35.9%) | 100.7 (14.5%) | 106 (15.3%) |
|  | 2 | TxV 1 | 17.6 (1.1%) | 174 (11.2%) | 464.9 (29.9%) | 338.8 (21.8%) | 558.6 (35.9%) |
|  | 2 | TxV 2 | 8 (1.8%) | 78.3 (17.1%) | 175.6 (38.3%) | 132.5 (28.9%) | 63.6 (13.9%) |
| **PxV status quo,Screening 2050** | | |  |  |  |  |  |
|  | 1 | TxV 1^*^ | 40.5 (7.7%) | 188.4 (35.6%) | 187.8 (35.5%) | 60.9 (11.5%) | 52.2 (9.8%) |
|  | 1 | TxV 1^#^ | 42.3 (5.5%) | 211.3 (27.3%) | 285.1 (36.8%) | 122.6 (15.8%) | 112.6 (14.5%) |
|  | 2 | TxV 1 | 16.8 (1.1%) | 173.2 (11.4%) | 465.6 (30.7%) | 341.4 (22.5%) | 520.5 (34.3%) |
|  | 2 | TxV 2 | 8 (1.9%) | 72 (16.9%) | 162.4 (38%) | 124.1 (29.1%) | 60.6 (14.2%) |
|  |  |  |  |  |  |  |  |

Base case = two-dose prophylactic HPV vaccine coverage among 13-year-old girls increases linearly to 90% by 2030, while screening coverage remains unchanged.

Status quo = current prophylactic HPV vaccination or/and screening&treatment practices; PxV 2030/2050 = two-dose prophylactic HPV vaccine coverage among 13-year-old girls increases linearly to 90% by 2030/2050; Screening 2030/2050 = HPV DNA-based screening coverage increases linearly to 90% by 2030/2050; TxV0 = no therapeutic vaccination; Use Case 1 (TxV1*) = population-based vaccination with a type 1 therapeutic vaccine targeting clearance of oncogenic HPV infections, administered annually to women aged 30 years; Use Case 1 (TxV1#) = same as TxV1*, with an initial catch-up campaign for women aged 31-45 years in the first year of roll-out; Use Case 2 (TxV1) = screening-based vaccination with a type 1 therapeutic vaccine targeting HPV-positive, triage-negative women; Use Case 2 (TxV2) = screening-based vaccination with a type 2 therapeutic vaccine targeting women diagnosed with CIN2/3 through HPV-based screening.

**Table S7.** Estimated cervical cancer cases across different scenarios in urban areas in China (thousands)

| **Scenario** | **Use case** | **Type of TxV** | **Cervical cancer cases** | | | | |
| --- | --- | --- | --- | --- | --- | --- | --- |
|  |  |  | **2025-2050** | **2051-2075** | **2076-2100** | **2101-2125** | **2025-2125** |
| **Base case (PxV 2030, Screening status quo)** | | |  |  |  |  |  |
|  | - | TxV 0 | 1570.9 (1521.5–1652.3) | 951.5 (917.3–1015.4) | 445.2 (427.9–476.0) | 258.9 (247.2–280.6) | 3226.4 (3116.4–3431.2) |
|  | 1 | TxV 1^*^ | 1550.6 (1502.1–1633.5) | 902.8 (868.6–961.6) | 434.9 (417.6–463.9) | 258.6 (247.0–280.2) | 3146.2 (3036.9–3339.6) |
|  | 1 | TxV 1^#^ | 1480.8 (1430.8–1563.9) | 840.8 (803.5–898.0) | 417.0 (399.0–446.4) | 258.5 (246.9–280.0) | 2997.3 (2883.6–3189.1) |
|  | 2 | TxV 1 | 1476.5 (1426.5–1560.0) | 716.4 (675.3–772.1) | 360.4 (342.4–389.1) | 257.5 (245.7–278.6) | 2815.2 (2693.5–2999.6) |
|  | 2 | TxV 2 | 1532.2 (1486.6–1616.2) | 918.8 (888.7–982.6) | 440.4 (424.0–470.3) | 258.9 (247.2–280.4) | 3148.2 (3048.8–3349.1) |
| **PxV 2050,Screening status quo** | | |  |  |  |  |  |
|  | - | TxV 0 | 1574.1 (1525.0–1655.9) | 1025.2 (988.8–1092.5) | 508.4 (490.2–542.7) | 278.4 (266.5–300.1) | 3384.2 (3269.1–3593.9) |
|  | 1 | TxV 1^*^ | 1553.8 (1505.2–1636.2) | 948.2 (912.2–1012.5) | 480.5 (461.5–512.9) | 274.0 (261.9–295.5) | 3257.3 (3141.4–3455.3) |
|  | 1 | TxV 1^#^ | 1483.8 (1434.0–1567.2) | 885.9 (844.2–945.4) | 461.8 (441.7–495.0) | 273.3 (261.1–294.6) | 3105.1 (2984.1–3306.4) |
|  | 2 | TxV 1 | 1479.8 (1430.1–1563.6) | 757.9 (715.0–818.6) | 381.4 (359.5–411.4) | 262.8 (250.5–283.6) | 2885.8 (2758.4–3073.3) |
|  | 2 | TxV 2 | 1535.3 (1490.0–1619.2) | 987.0 (954.6–1053.0) | 499.2 (481.4–532.6) | 277.8 (266.0–299.6) | 3299.8 (3191.1–3501.5) |
| **PxV 2030,Screening 2030** | | |  |  |  |  |  |
|  | - | TxV 0 | 1393.7 (1332.6–1504.3) | 626.3 (589.6–698.9) | 287.0 (271.9–316.6) | 145.6 (134.9–166.0) | 2453.7 (2328.4–2686.4) |
|  | 1 | TxV 1^*^ | 1379.2 (1318.3–1489.4) | 598.8 (564.0–663.1) | 279.6 (264.6–307.9) | 145.4 (134.6–165.6) | 2402.9 (2283.8–2629.4) |
|  | 1 | TxV 1^#^ | 1323.7 (1264.1–1429.9) | 560.9 (524.9–625.9) | 265.1 (248.2–293.8) | 145.3 (134.5–165.4) | 2292.6 (2172.3–2508.2) |
|  | 2 | TxV 1 | 1291.2 (1230.9–1399.4) | 404.1 (366.7–457.0) | 195.8 (179.7–221.6) | 144.1 (132.8–163.9) | 2037.2 (1913.5–2244.7) |
|  | 2 | TxV 2 | 1343.5 (1286.4–1450.8) | 585.9 (552.5–656.8) | 282.3 (267.2–311.2) | 145.5 (134.8–165.8) | 2355.9 (2242.3–2587.9) |
| **PxV 2050,Screening 2050** | | |  |  |  |  |  |
|  | - | TxV 0 | 1644.5 (1589.5–1739.0) | 822.6 (776.2–907.3) | 331.1 (312.3–365.3) | 163.9 (152.4–184.4) | 2959.3 (2830.6–3203.0) |
|  | 1 | TxV 1^*^ | 1626.4 (1572.6–1721.8) | 764.8 (723.4–842.4) | 314.4 (296.4–347.1) | 159.8 (148.6–179.8) | 2864.4 (2740.3–3091.6) |
|  | 1 | TxV 1^#^ | 1554.7 (1500.4–1649.5) | 716.5 (673.8–791.4) | 297.8 (278.6–331.5) | 159.2 (148.0–179.1) | 2726.2 (2600.2–2953.9) |
|  | 2 | TxV 1 | 1552.2 (1496.9–1649.2) | 580.9 (533.1–647.3) | 214.1 (195.3–243.6) | 146.2 (135.2–166.0) | 2495.7 (2364.0–2706.6) |
|  | 2 | TxV 2 | 1603.2 (1551.7–1697.3) | 775.6 (735.8–859.9) | 320.6 (303.3–354.5) | 163.4 (151.9–183.7) | 2861.1 (2742.0–3096.6) |
| **PxV status quo, Screening status quo** | | |  |  |  |  |  |
|  | - | TxV 0 | 1576.7 (1527.6–1658.5) | 1131.9 (1091.9–1205.3) | 827.3 (797.2–882.2) | 670.6 (646.0–715.7) | 4204.4 (4062.2–4464.7) |
|  | 1 | TxV 1^*^ | 1556.2 (1507.6–1638.6) | 1020.7 (980.1–1088.6) | 700.5 (666.4–747.3) | 561.6 (533.3–601.3) | 3839.3 (3692.3–4087.1) |
|  | 1 | TxV 1^#^ | 1486.0 (1436.3–1569.6) | 956.8 (911.6–1020.5) | 680.7 (644.8–729.0) | 560.2 (531.6–599.5) | 3688.1 (3527.2–3920.5) |
|  | 2 | TxV 1 | 1482.3 (1432.6–1566.2) | 834.0 (786.9–900.6) | 550.6 (516.6–601.9) | 443.4 (415.5–484.7) | 3318.4 (3152.9–3546.8) |
|  | 2 | TxV 2 | 1537.8 (1492.5–1621.8) | 1086.5 (1050.8–1158.9) | 793.5 (766.8–847.6) | 643.5 (621.4–687.3) | 4061.5 (3930.9–4312.0) |
| **PxV status quo,Screening 2030** | | |  |  |  |  |  |
|  | - | TxV 0 | 1399.9 (1338.2–1510.7) | 759.8 (711.3–844.6) | 532.9 (497.2–594.1) | 429.1 (399.7–479.5) | 3117.9 (2945.9–3430.6) |
|  | 1 | TxV 1^*^ | 1384.8 (1323.9–1495.2) | 688.5 (645.5–766.2) | 453.9 (422.2–507.2) | 360.2 (333.8–403.2) | 2884.3 (2724.9–3173.0) |
|  | 1 | TxV 1^#^ | 1329.0 (1269.3–1435.7) | 648.5 (605.9–722.8) | 437.7 (403.3–490.2) | 358.9 (332.3–401.8) | 2772.2 (2614.7–3055.8) |
|  | 2 | TxV 1 | 1297.2 (1237.0–1405.6) | 477.5 (433.9–543.1) | 284.4 (255.7–326.9) | 226.7 (203.2–261.3) | 2289.2 (2131.0–2525.6) |
|  | 2 | TxV 2 | 1349.2 (1292.5–1457.0) | 699.4 (656.9–787.4) | 487.4 (456.0–548.8) | 391.8 (365.9–442.3) | 2927.2 (2771.2–3240.7) |
| **PxV status quo,Screening 2050** | | |  |  |  |  |  |
|  | - | TxV 0 | 1647.2 (1592.1–1741.7) | 908.1 (857.2–1001.5) | 538.9 (502.8–601.4) | 429.1 (399.7–479.5) | 3520.2 (3352.1–3831.1) |
|  | 1 | TxV 1^*^ | 1628.8 (1575.0–1724.3) | 824.5 (778.4–909.1) | 459.8 (427.7–515.1) | 360.2 (333.8–403.3) | 3271.7 (3114.1–3554.6) |
|  | 1 | TxV 1^#^ | 1557.0 (1502.7–1651.8) | 776.5 (727.6–855.6) | 443.4 (408.6–497.5) | 358.9 (332.3–401.9) | 3136.1 (2975.7–3413.2) |
|  | 2 | TxV 1 | 1554.7 (1499.4–1651.8) | 637.2 (584.5–711.2) | 299.2 (269.0–344.4) | 227.7 (204.1–262.4) | 2720.3 (2557.8–2959.4) |
|  | 2 | TxV 2 | 1605.7 (1554.2–1699.9) | 851.7 (807.2–944.4) | 493.4 (462.1–557.0) | 391.8 (366.0–442.4) | 3342.3 (3189.4–3647.4) |
|  |  |  |  |  |  |  |  |

Base case = two-dose prophylactic HPV vaccine coverage among 13-year-old girls increases linearly to 90% by 2030, while screening coverage remains unchanged.

Status quo = current prophylactic HPV vaccination or/and screening&treatment practices; PxV 2030/2050 = two-dose prophylactic HPV vaccine coverage among 13-year-old girls increases linearly to 90% by 2030/2050; Screening 2030/2050 = HPV DNA-based screening coverage increases linearly to 90% by 2030/2050; TxV0 = no therapeutic vaccination; Use Case 1 (TxV1*) = population-based vaccination with a type 1 therapeutic vaccine targeting clearance of oncogenic HPV infections, administered annually to women aged 30 years; Use Case 1 (TxV1#) = same as TxV1*, with an initial catch-up campaign for women aged 31-45 years in the first year of roll-out; Use Case 2 (TxV1) = screening-based vaccination with a type 1 therapeutic vaccine targeting HPV-positive, triage-negative women; Use Case 2 (TxV2) = screening-based vaccination with a type 2 therapeutic vaccine targeting women diagnosed with CIN2/3 through HPV-based screening.

**Table S8.** Estimated cervical cancer cases across different scenarios in rural areas in China (thousands)

| **Scenario** | **Use case** | **Type of TxV** | **Cervical cancer cases** | | | | |
| --- | --- | --- | --- | --- | --- | --- | --- |
|  |  |  | **2025-2050** | **2051-2075** | **2076-2100** | **2101-2125** | **2025-2125** |
| **Base case (PxV 2030, Screening status quo)** | | |  |  |  |  |  |
|  | - | TxV 0 | 1402.2 (1364.2–1455.9) | 980.4 (948.9–1031.5) | 552.7 (534.0–581.7) | 374.8 (360.1–398.7) | 3310.9 (3207.8–3467.8) |
|  | 1 | TxV 1^*^ | 1380.0 (1342.4–1434.5) | 930.5 (897.7–976.8) | 540.9 (522.0–568.9) | 374.4 (359.5–398.1) | 3228.5 (3122.8–3375.7) |
|  | 1 | TxV 1^#^ | 1322.7 (1283.8–1374.7) | 865.1 (828.1–913.1) | 521.0 (500.6–548.5) | 374.2 (359.3–397.9) | 3084.2 (2975.2–3227.3) |
|  | 2 | TxV 1 | 1333.1 (1294.0–1385.4) | 754.2 (714.5–802.6) | 454.4 (434.6–481.5) | 372.7 (357.8–396.2) | 2912.5 (2806.7–3061.4) |
|  | 2 | TxV 2 | 1363.0 (1328.5–1413.3) | 939.3 (910.6–986.7) | 546.1 (528.3–574.3) | 374.8 (360.0–398.6) | 3225.7 (3129.0–3367.8) |
| **PxV 2050,Screening status quo** | | |  |  |  |  |  |
|  | - | TxV 0 | 1405.1 (1367.0–1458.9) | 1051.2 (1016.2–1105.1) | 624.8 (604.5–656.3) | 399.9 (384.7–423.8) | 3482.1 (3372.2–3645.9) |
|  | 1 | TxV 1^*^ | 1382.7 (1345.0–1437.2) | 974.4 (939.3–1022.1) | 593.0 (572.1–623.0) | 393.9 (378.4–417.4) | 3346.1 (3234.0–3498.7) |
|  | 1 | TxV 1^#^ | 1325.2 (1286.4–1377.1) | 906.9 (867.8–959.3) | 571.6 (549.4–601.9) | 393.4 (377.5–416.5) | 3197.2 (3084.8–3350.2) |
|  | 2 | TxV 1 | 1336.0 (1296.5–1387.7) | 796.6 (754.6–847.2) | 480.0 (456.2–510.3) | 380.6 (364.3–403.4) | 2991.0 (2877.2–3145.6) |
|  | 2 | TxV 2 | 1365.5 (1330.8–1416.2) | 1003.0 (971.9–1053.6) | 612.2 (593.0–642.2) | 399.1 (384.0–422.8) | 3380.9 (3280.2–3530.3) |
| **PxV 2030,Screening 2030** | | |  |  |  |  |  |
|  | - | TxV 0 | 1238.8 (1186.8–1315.8) | 635.5 (594.5–701.2) | 355.0 (335.1–385.4) | 212.6 (196.2–237.5) | 2444.7 (2316.9–2640.1) |
|  | 1 | TxV 1^*^ | 1222.3 (1171.2–1297.6) | 608.5 (567.7–666.8) | 346.6 (327.0–377.0) | 212.2 (195.8–237.1) | 2388.6 (2261.2–2578.2) |
|  | 1 | TxV 1^#^ | 1178.4 (1127.7–1247.0) | 569.2 (529.6–624.1) | 330.4 (310.0–360.5) | 212.1 (195.6–236.9) | 2290.7 (2164.9–2471.1) |
|  | 2 | TxV 1 | 1153.3 (1102.2–1224.2) | 410.9 (374.4–461.6) | 245.8 (226.1–273.2) | 210.2 (193.8–235.0) | 2021.8 (1901.5–2186.5) |
|  | 2 | TxV 2 | 1180.8 (1133.7–1251.2) | 577.1 (541.0–637.9) | 347.7 (328.3–376.7) | 212.6 (196.1–237.4) | 2318.8 (2200.5–2505.3) |
| **PxV 2050,Screening 2050** | | |  |  |  |  |  |
|  | - | TxV 0 | 1420.1 (1376.4–1481.4) | 815.0 (768.0–888.0) | 405.1 (381.5–442.0) | 235.6 (218.8–260.8) | 2878.5 (2745.0–3073.2) |
|  | 1 | TxV 1^*^ | 1400.2 (1357.2–1461.7) | 759.6 (715.2–825.5) | 386.4 (363.1–421.9) | 230.2 (213.4–255.9) | 2776.4 (2649.8–2960.5) |
|  | 1 | TxV 1^#^ | 1343.5 (1300.3–1401.3) | 710.6 (665.6–771.3) | 368.4 (343.9–402.6) | 229.4 (212.8–255.2) | 2652.0 (2525.9–2828.9) |
|  | 2 | TxV 1 | 1347.3 (1303.6–1406.3) | 575.0 (530.7–633.9) | 266.3 (244.4–297.5) | 213.0 (196.4–238.8) | 2401.8 (2278.8–2570.9) |
|  | 2 | TxV 2 | 1374.7 (1335.6–1432.0) | 748.4 (707.6–815.9) | 388.8 (367.3–422.7) | 234.6 (217.9–259.8) | 2748.9 (2628.0–2935.3) |
| **PxV status quo, Screening status quo** | | |  |  |  |  |  |
|  | - | TxV 0 | 1407.3 (1369.2–1461.1) | 1162.3 (1123.9–1221.3) | 1024.8 (990.5–1077.6) | 960.4 (927.9–1010.1) | 4556.9 (4412.1–4770.9) |
|  | 1 | TxV 1^*^ | 1384.6 (1346.9–1439.3) | 1050.0 (1010.8–1103.5) | 875.0 (836.1–924.3) | 812.3 (774.6–859.5) | 4119.1 (3974.3–4323.2) |
|  | 1 | TxV 1^#^ | 1327.2 (1288.3–1379.1) | 982.1 (937.7–1038.6) | 851.9 (810.3–903.0) | 810.2 (772.3–857.6) | 3970.3 (3811.0–4181.8) |
|  | 2 | TxV 1 | 1338.0 (1298.6–1389.8) | 880.2 (834.2–936.7) | 707.4 (666.8–761.3) | 659.4 (621.0–709.9) | 3584.3 (3422.8–3792.5) |
|  | 2 | TxV 2 | 1367.7 (1332.9–1418.3) | 1104.5 (1070.8–1159.6) | 974.0 (943.8–1023.5) | 912.8 (883.9–959.6) | 4360.8 (4232.2–4563.5) |
| **PxV status quo,Screening 2030** | | |  |  |  |  |  |
|  | - | TxV 0 | 1244.0 (1191.7–1321.0) | 771.3 (720.7–848.5) | 660.0 (615.5–726.9) | 614.7 (572.6–677.8) | 3292.8 (3102.7–3575.1) |
|  | 1 | TxV 1^*^ | 1227.0 (1175.4–1302.1) | 700.5 (653.1–770.8) | 568.3 (526.5–622.6) | 522.9 (482.2–575.3) | 3021.9 (2842.0–3271.4) |
|  | 1 | TxV 1^#^ | 1183.0 (1132.1–1251.5) | 659.4 (611.4–722.6) | 549.2 (506.6–606.2) | 521.4 (480.7–573.6) | 2913.7 (2735.6–3151.8) |
|  | 2 | TxV 1 | 1158.0 (1107.1–1228.6) | 487.9 (444.6–550.3) | 355.1 (320.7–405.0) | 328.3 (296.5–375.2) | 2328.6 (2172.3–2551.5) |
|  | 2 | TxV 2 | 1185.4 (1138.0–1256.2) | 685.4 (641.0–759.1) | 583.5 (545.6–648.5) | 542.4 (506.5–604.3) | 2997.2 (2834.2–3263.0) |
| **PxV status quo,Screening 2050** | | |  |  |  |  |  |
|  | - | TxV 0 | 1422.3 (1378.6–1483.6) | 904.9 (852.5–985.6) | 667.5 (622.3–736.4) | 614.8 (572.6–677.9) | 3612.8 (3425.4–3884.4) |
|  | 1 | TxV 1^*^ | 1402.2 (1359.2–1463.8) | 822.8 (774.4–892.5) | 575.5 (533.0–631.5) | 522.9 (482.2–575.3) | 3323.2 (3154.2–3563.4) |
|  | 1 | TxV 1^#^ | 1345.5 (1302.2–1403.3) | 772.3 (723.3–840.2) | 556.1 (513.2–614.2) | 521.4 (480.7–573.6) | 3197.2 (3023.0–3427.0) |
|  | 2 | TxV 1 | 1349.5 (1305.7–1408.5) | 634.5 (585.5–700.9) | 372.3 (335.7–423.9) | 329.7 (297.6–376.7) | 2688.0 (2528.2–2906.5) |
|  | 2 | TxV 2 | 1376.8 (1337.7–1434.2) | 823.6 (778.2–898.0) | 590.8 (551.8–657.0) | 542.5 (506.5–604.3) | 3336.3 (3178.7–3587.9) |

Base case = two-dose prophylactic HPV vaccine coverage among 13-year-old girls increases linearly to 90% by 2030, while screening coverage remains unchanged.

Status quo = current prophylactic HPV vaccination or/and screening&treatment practices; PxV 2030/2050 = two-dose prophylactic HPV vaccine coverage among 13-year-old girls increases linearly to 90% by 2030/2050; Screening 2030/2050 = HPV DNA-based screening coverage increases linearly to 90% by 2030/2050; TxV0 = no therapeutic vaccination; Use Case 1 (TxV1*) = population-based vaccination with a type 1 therapeutic vaccine targeting clearance of oncogenic HPV infections, administered annually to women aged 30 years; Use Case 1 (TxV1#) = same as TxV1*, with an initial catch-up campaign for women aged 31-45 years in the first year of roll-out; Use Case 2 (TxV1) = screening-based vaccination with a type 1 therapeutic vaccine targeting HPV-positive, triage-negative women; Use Case 2 (TxV2) = screening-based vaccination with a type 2 therapeutic vaccine targeting women diagnosed with CIN2/3 through HPV-based screening.

**Table S9.** Estimated cervical cancer cases averted by therapeutic vaccines across different scenarios in China (thousands)

| **Scenario** | **Use case** | **Type of TxV** | **Cases of cervical cancer averted by TxV** | | | | |  | **Reduction of case vs TxV0** | | | | |
| --- | --- | --- | --- | --- | --- | --- | --- | --- | --- | --- | --- | --- | --- |
|  |  |  | **2025-**  **2050** | **2051-**  **2075** | **2076-**  **2100** | **2101-**  **2125** | **2025-**  **2125** |  | **2025-**  **2050** | **2051-**  **2075** | **2076-**  **2100** | **2101-**  **2125** | **2025-**  **2125** |
| **Base case (PxV 2030, Screening status quo)** | | | | | | | | | | | | | |
|  | - | TxV 0 | REF | REF | REF | REF | REF |  | REF | REF | REF | REF | REF |
|  | 1 | TxV 1^*^ | 41.8 (34.5–46.4) | 102.8 (83.9–115.6) | 23.8 (18.8–27.4) | 0.7 (0.4–1.3) | 169.6 (137.8–189.3) |  | 1.4% (1.2–1.5%) | 5.3% (4.4–5.9%) | 2.4% (1.9–2.7%) | 0.1% (0.1–0.2%) | 2.6% (2.1–2.9%) |
|  | 1 | TxV 1^#^ | 174.1 (141.7–195.6) | 235.6 (189.6–266.1) | 62.9 (49.3–71.2) | 0.9 (0.6–1.7) | 474.0 (380.9–534.5) |  | 5.9% (4.8–6.5%) | 12.1% (9.9–13.6%) | 6.2% (4.9–7.1%) | 0.1% (0.1–0.3%) | 7.2% (5.9–8.1%) |
|  | 2 | TxV 1 | 167.7 (140.5–185.6) | 476.1 (412.0–519.2) | 187.7 (165.8–200.6) | 3.4 (2.5–5.7) | 834.0 (723.2–908.2) |  | 5.6% (4.7–6.2%) | 24.5% (21.3–26.5%) | 18.7% (16.5–20.0%) | 0.5% (0.4–0.9%) | 12.7% (11.1–13.7%) |
|  | 2 | TxV 2 | 76.5 (58.5–95.0) | 72.3 (55.9–89.4) | 11.5 (8.8–14.4) | 0.1 (0.1–0.2) | 160.4 (123.3–198.8) |  | 2.6% (2.0–3.2%) | 3.7% (2.9–4.6%) | 1.1% (0.9–1.4%) | 0.0% (0.0–0.0%) | 2.4% (1.9–3.0%) |
| **PxV 2050,Screening status quo** | | | | | | | | | | | | | |
|  | - | TxV 0 | REF | REF | REF | REF | REF |  | REF | REF | REF | REF | REF |
|  | 1 | TxV 1^*^ | 42.4 (35.0–47.0) | 158.8 (131.3–178.5) | 62.6 (50.3–70.6) | 11.0 (8.7–12.2) | 275.3 (225.0–308.9) |  | 1.4% (1.2–1.6%) | 7.6% (6.3–8.4%) | 5.5% (4.5–6.1%) | 1.6% (1.3–1.8%) | 4.0% (3.3–4.4%) |
|  | 1 | TxV 1^#^ | 174.8 (142.3–196.4) | 295.1 (239.0–332.6) | 104.5 (82.7–118.2) | 12.4 (9.8–14.0) | 586.9 (473.2–660.9) |  | 5.9% (4.8–6.5%) | 14.1% (11.5–15.7%) | 9.2% (7.3–10.3%) | 1.8% (1.4–2.1%) | 8.5% (6.9–9.5%) |
|  | 2 | TxV 1 | 168.0 (140.7–185.8) | 535.6 (464.7–585.9) | 279.6 (248.3–299.6) | 36.2 (32.5–38.1) | 1020.8 (886.2–1109.1) |  | 5.6% (4.7–6.2%) | 25.7% (22.2–27.8%) | 24.5% (21.8–26.1%) | 5.3% (4.7–5.6%) | 14.8% (12.8–15.9%) |
|  | 2 | TxV 2 | 76.7 (58.6–95.3) | 84.8 (65.4–104.9) | 21.8 (16.9–27.0) | 1.5 (1.1–1.8) | 184.6 (142.1–229.4) |  | 2.6% (2.0–3.2%) | 4.1% (3.1–5.0%) | 1.9% (1.5–2.4%) | 0.2% (0.2–0.3%) | 2.7% (2.1–3.3%) |
| **PxV 2030,Screening 2030** | | | | | | | | | | | | | |
|  | - | TxV 0 | REF | REF | REF | REF | REF |  | REF | REF | REF | REF | REF |
|  | 1 | TxV 1^*^ | 32.2 (26.7–37.0) | 57.2 (46.3–69.4) | 16.3 (12.8–18.7) | 0.6 (0.4–1.1) | 107.2 (86.6–125.6) |  | 1.2% (1.0–1.4%) | 4.6% (3.7–5.1%) | 2.5% (2.0–2.9%) | 0.2% (0.1–0.3%) | 2.2% (1.8–2.4%) |
|  | 1 | TxV 1^#^ | 134.5 (110.2–157.9) | 138.8 (110.6–164.7) | 50.1 (39.2–57.2) | 0.8 (0.6–1.5) | 323.2 (261.7–379.9) |  | 5.2% (4.2–5.8%) | 11.0% (8.9–12.3%) | 7.7% (6.1–8.8%) | 0.2% (0.2–0.4%) | 6.7% (5.4–7.4%) |
|  | 2 | TxV 1 | 194.2 (161.7–220.3) | 458.5 (400.9–511.2) | 205.6 (188.4–216.2) | 4.0 (3.0–6.5) | 862.5 (755.9–952.2) |  | 7.4% (6.1–8.2%) | 36.2% (32.1–38.8%) | 31.7% (28.7–33.7%) | 1.1% (0.8–1.8%) | 17.6% (15.5–18.8%) |
|  | 2 | TxV 2 | 108.8 (80.7–137.6) | 98.7 (75.4–122.2) | 12.6 (9.7–15.7) | 0.1 (0.1–0.2) | 219.7 (166.4–275.1) |  | 4.1% (3.1–5.2%) | 7.8% (6.1–9.5%) | 1.9% (1.5–2.4%) | 0.0% (0.0–0.1%) | 4.4% (3.4–5.5%) |
| **PxV 2050,Screening 2050** | | | | | | | | | | | | | |
|  | - | TxV 0 | REF | REF | REF | REF | REF |  | REF | REF | REF | REF | REF |
|  | 1 | TxV 1^*^ | 38.2 (31.7–43.0) | 116.0 (95.5–136.4) | 37.5 (29.9–43.6) | 9.6 (7.6–10.8) | 200.8 (164.5–233.5) |  | 1.3% (1.0–1.4%) | 7.1% (5.9–7.9%) | 5.1% (4.1–5.7%) | 2.4% (1.9–2.7%) | 3.5% (2.8–3.8%) |
|  | 1 | TxV 1^#^ | 170.6 (139.2–193.9) | 217.2 (176.1–254.1) | 73.8 (57.8–85.4) | 11.0 (8.6–12.4) | 472.8 (380.9–546.4) |  | 5.6% (4.6–6.2%) | 13.3% (10.8–14.8%) | 10.0% (7.9–11.2%) | 2.7% (2.1–3.1%) | 8.1% (6.6–9.1%) |
|  | 2 | TxV 1 | 169.2 (140.5–190.3) | 494.6 (427.2–553.2) | 261.1 (237.0–280.8) | 40.5 (37.7–42.0) | 963.8 (841.7–1064.3) |  | 5.5% (4.5–6.1%) | 30.2% (26.1–32.6%) | 35.3% (31.9–37.4%) | 10.0% (9.0–10.8%) | 16.5% (14.4–17.8%) |
|  | 2 | TxV 2 | 85.0 (62.8–109.1) | 111.8 (84.3–139.9) | 27.0 (20.7–33.3) | 1.5 (1.2–1.9) | 225.4 (168.6–284.8) |  | 2.8% (2.0–3.5%) | 6.7% (5.1–8.4%) | 3.6% (2.8–4.4%) | 0.4% (0.3–0.5%) | 3.8% (2.9–4.8%) |
| **PxV status quo, Screening status quo** | | | | | | | | | | | | | |
|  | - | TxV 0 | REF | REF | REF | REF | REF |  | REF | REF | REF | REF | REF |
|  | 1 | TxV 1^*^ | 42.8 (35.3–47.5) | 231.3 (191.3–259.4) | 287.4 (233.5–324.0) | 267.4 (216.2–302.0) | 829.3 (675.6–933.4) |  | 1.4% (1.2–1.6%) | 10.1% (8.4–11.1%) | 15.4% (12.6–17.1%) | 16.3% (13.3–18.1%) | 9.4% (7.7–10.4%) |
|  | 1 | TxV 1^#^ | 175.3 (142.7–197.0) | 369.4 (300.0–415.2) | 331.9 (267.0–374.1) | 271.1 (219.1–306.1) | 1148.8 (928.2–1292.7) |  | 5.9% (4.8–6.5%) | 16.0% (13.1–17.7%) | 17.9% (14.5–19.8%) | 16.6% (13.4–18.4%) | 13.0% (10.6–14.5%) |
|  | 2 | TxV 1 | 168.1 (140.9–186.0) | 595.1 (515.3–651.6) | 606.0 (532.0–655.7) | 538.9 (474.4–582.5) | 1908.8 (1661.0–2076.9) |  | 5.6% (4.7–6.2%) | 25.9% (22.4–27.9%) | 32.6% (28.7–34.8%) | 32.9% (29.1–35.1%) | 21.7% (18.9–23.3%) |
|  | 2 | TxV 2 | 76.8 (58.8–95.5) | 101.3 (78.1–125.2) | 82.9 (64.2–102.0) | 74.0 (57.2–90.7) | 335.1 (258.8–415.4) |  | 2.6% (2.0–3.2%) | 4.4% (3.4–5.4%) | 4.4% (3.5–5.4%) | 4.5% (3.5–5.5%) | 3.8% (2.9–4.7%) |
| **PxV status quo,Screening 2030** | | | | | | | | | | | | | |
|  | - | TxV 0 | REF | REF | REF | REF | REF |  | REF | REF | REF | REF | REF |
|  | 1 | TxV 1^*^ | 33.3 (27.6–38.2) | 146.2 (120.6–173.9) | 175.8 (142.5–209.6) | 166.2 (133.4–197.8) | 521.4 (423.5–619.6) |  | 1.3% (1.0–1.4%) | 9.6% (8.0–10.6%) | 14.9% (12.1–16.5%) | 16.0% (12.9–17.9%) | 8.2% (6.7–9.1%) |
|  | 1 | TxV 1^#^ | 135.8 (111.1–159.4) | 229.6 (186.7–272.1) | 212.6 (170.7–251.2) | 168.9 (135.4–200.8) | 746.0 (603.2–885.2) |  | 5.2% (4.2–5.8%) | 15.1% (12.3–16.8%) | 17.9% (14.4–20.0%) | 16.2% (13.1–18.2%) | 11.7% (9.5–13.1%) |
|  | 2 | TxV 1 | 194.7 (162.1–220.8) | 574.4 (503.4–647.6) | 557.9 (502.9–619.4) | 493.6 (445.7–546.2) | 1820.7 (1614.4–2036.5) |  | 7.4% (6.1–8.1%) | 37.7% (33.0–40.2%) | 47.0% (42.6–49.3%) | 47.4% (43.2–49.7%) | 28.5% (25.3–30.3%) |
|  | 2 | TxV 2 | 109.2 (81.0–138.1) | 144.1 (110.3–177.8) | 120.3 (92.3–146.8) | 108.3 (83.5–132.1) | 481.0 (366.6–594.0) |  | 4.1% (3.1–5.2%) | 9.3% (7.2–11.4%) | 10.0% (7.8–12.1%) | 10.2% (8.0–12.5%) | 7.4% (5.7–9.1%) |
| **PxV status quo,Screening 2050** | | | | | | | | | | | | | |
|  | - | TxV 0 | REF | REF | REF | REF | REF |  | REF | REF | REF | REF | REF |
|  | 1 | TxV 1^*^ | 38.7 (32.1–43.5) | 170.8 (140.7–199.6) | 176.2 (142.9–210.0) | 166.2 (133.4–197.8) | 552.2 (447.9–651.0) |  | 1.3% (1.0–1.4%) | 9.5% (7.8–10.4%) | 14.8% (12.0–16.4%) | 16.0% (12.9–17.9%) | 7.8% (6.3–8.7%) |
|  | 1 | TxV 1^#^ | 171.1 (139.7–194.5) | 273.4 (223.2–319.4) | 213.6 (171.4–252.5) | 168.9 (135.4–200.8) | 826.1 (669.2–967.9) |  | 5.6% (4.6–6.2%) | 15.2% (12.3–16.8%) | 17.8% (14.3–19.9%) | 16.2% (13.1–18.2%) | 11.6% (9.4–13.0%) |
|  | 2 | TxV 1 | 169.3 (140.7–190.5) | 555.3 (478.9–622.9) | 540.5 (483.0–602.8) | 491.1 (443.1–544.1) | 1759.4 (1545.1–1963.3) |  | 5.5% (4.5–6.1%) | 30.6% (26.4–33.1%) | 44.9% (40.5–47.5%) | 47.2% (43.0–49.5%) | 24.8% (21.8–26.4%) |
|  | 2 | TxV 2 | 85.2 (62.9–109.3) | 135.6 (102.3–169.7) | 120.5 (92.5–147.3) | 108.3 (83.5–132.1) | 449.5 (341.1–558.1) |  | 2.8% (2.0–3.5%) | 7.4% (5.6–9.2%) | 9.9% (7.7–12.1%) | 10.2% (8.0–12.5%) | 6.2% (4.8–7.7%) |

The number of cervical cancer cases and deaths averted was calculated as the difference between outcomes in the TxV vaccination and no-vaccination scenarios. The reduction in cases or deaths was defined as the number of cases or deaths averted divided by the corresponding number in the scenario without TxV vaccination. Base case = two-dose prophylactic HPV vaccine coverage among 13-year-old girls increases linearly to 90% by 2030, while screening coverage remains unchanged.

Status quo = current prophylactic HPV vaccination or/and screening&treatment practices; PxV 2030/2050 = two-dose prophylactic HPV vaccine coverage among 13-year-old girls increases linearly to 90% by 2030/2050; Screening 2030/2050 = HPV DNA-based screening coverage increases linearly to 90% by 2030/2050; TxV0 = no therapeutic vaccination; Use Case 1 (TxV1*) = population-based vaccination with a type 1 therapeutic vaccine targeting clearance of oncogenic HPV infections, administered annually to women aged 30 years; Use Case 1 (TxV1#) = same as TxV1*, with an initial catch-up campaign for women aged 31-45 years in the first year of roll-out; Use Case 2 (TxV1) = screening-based vaccination with a type 1 therapeutic vaccine targeting HPV-positive, triage-negative women; Use Case 2 (TxV2) = screening-based vaccination with a type 2 therapeutic vaccine targeting women diagnosed with CIN2/3 through HPV-based screening.

**Table S10.** Estimated cervical cancer cases averted by therapeutic vaccines across different scenarios in urban and rural areas in China, 2025-2125 (thousands)

| **Scenario** | **Use case** | **Type of TxV** | **Cases of cervical cancer averted by TxV** | |  | **Reduction of case vs TxV0** | |
| --- | --- | --- | --- | --- | --- | --- | --- |
|  |  |  | **Urban** | **Rural** |  | **Urban** | **Rural** |
| **Base case (PxV 2030, Screening status quo)** | | |  |  |  |  |  |
|  | - | TxV 0 | REF | REF |  | REF | REF |
|  | 1 | TxV 1^*^ | 80.0 (65.4–90.5) | 89.3 (72.7–99.7) |  | 2.5% (2.0–2.7%) | 2.7% (2.2–3.0%) |
|  | 1 | TxV 1^#^ | 235.3 (191.0–267.7) | 236.8 (190.2–267.6) |  | 7.3% (6.0–8.2%) | 7.1% (5.8–8.0%) |
|  | 2 | TxV 1 | 426.2 (368.6–467.3) | 407.9 (352.3–442.0) |  | 13.2% (11.4–14.2%) | 12.3% (10.7–13.3%) |
|  | 2 | TxV 2 | 70.8 (54.1–94.2) | 85.8 (63.6–109.6) |  | 2.2% (1.7–2.9%) | 2.6% (1.9–3.3%) |
| **PxV 2050,Screening status quo** | | |  |  |  |  |  |
|  | - | TxV 0 | REF | REF |  | REF | REF |
|  | 1 | TxV 1^*^ | 132.2 (108.7–148.8) | 142.7 (117.0–159.1) |  | 3.9% (3.2–4.3%) | 4.1% (3.4–4.5%) |
|  | 1 | TxV 1^#^ | 291.1 (236.2–329.8) | 294.6 (237.2–330.3) |  | 8.6% (7.0–9.6%) | 8.4% (6.8–9.4%) |
|  | 2 | TxV 1 | 517.7 (447.9–563.9) | 501.2 (433.0–543.7) |  | 15.2% (13.2–16.4%) | 14.4% (12.5–15.5%) |
|  | 2 | TxV 2 | 80.8 (61.7–107.5) | 99.5 (73.8–127.1) |  | 2.4% (1.8–3.2%) | 2.9% (2.1–3.6%) |
| **PxV 2030,Screening 2030** | | |  |  |  |  |  |
|  | - | TxV 0 | REF | REF |  | REF | REF |
|  | 1 | TxV 1^*^ | 50.2 (40.8–60.6) | 56.1 (45.9–66.6) |  | 2.1% (1.7–2.3%) | 2.3% (1.9–2.6%) |
|  | 1 | TxV 1^#^ | 162.6 (131.1–193.0) | 161.1 (129.5–188.5) |  | 6.7% (5.4–7.5%) | 6.6% (5.3–7.4%) |
|  | 2 | TxV 1 | 429.3 (373.4–478.6) | 433.6 (380.1–478.5) |  | 17.5% (15.4–18.8%) | 17.7% (15.8–18.9%) |
|  | 2 | TxV 2 | 91.1 (67.8–123.2) | 124.0 (90.9–159.1) |  | 3.7% (2.8–4.9%) | 5.1% (3.8–6.4%) |
| **PxV 2050,Screening 2050** | | |  |  |  |  |  |
|  | - | TxV 0 | REF | REF |  | REF | REF |
|  | 1 | TxV 1^*^ | 97.4 (79.4–114.2) | 103.7 (85.3–119.9) |  | 3.3% (2.7–3.7%) | 3.6% (3.0–4.0%) |
|  | 1 | TxV 1^#^ | 240.2 (193.6–277.9) | 232.4 (190.1–267.1) |  | 8.1% (6.6–9.1%) | 8.1% (6.5–9.1%) |
|  | 2 | TxV 1 | 480.3 (417.3–533.5) | 484.6 (424.9–532.8) |  | 16.2% (14.1–17.5%) | 16.9% (14.7–18.1%) |
|  | 2 | TxV 2 | 93.0 (68.0–127.3) | 127.1 (91.4–164.5) |  | 3.1% (2.3–4.2%) | 4.4% (3.2–5.6%) |
| **PxV status quo, Screening status quo** | | |  |  |  |  |  |
|  | - | TxV 0 | REF | REF |  | REF | REF |
|  | 1 | TxV 1^*^ | 379.5 (311.2–428.1) | 448.8 (366.9–502.2) |  | 9.0% (7.4–9.9%) | 9.8% (8.0–10.9%) |
|  | 1 | TxV 1^#^ | 541.5 (441.4–613.0) | 604.4 (488.9–676.8) |  | 12.9% (10.5–14.3%) | 13.2% (10.7–14.7%) |
|  | 2 | TxV 1 | 915.2 (794.9–997.5) | 993.0 (859.6–1075.5) |  | 21.7% (18.9–23.3%) | 21.8% (18.9–23.4%) |
|  | 2 | TxV 2 | 134.9 (103.1–179.5) | 192.8 (142.9–245.3) |  | 3.2% (2.5–4.2%) | 4.2% (3.2–5.3%) |
| **PxV status quo,Screening 2030** | | |  |  |  |  |  |
|  | - | TxV 0 | REF | REF |  | REF | REF |
|  | 1 | TxV 1^*^ | 238.1 (193.6–285.2) | 282.8 (231.1–334.4) |  | 7.7% (6.3–8.6%) | 8.7% (7.0–9.7%) |
|  | 1 | TxV 1^#^ | 354.5 (285.9–423.2) | 392.1 (319.2–462.5) |  | 11.5% (9.3–12.8%) | 12.0% (9.7–13.4%) |
|  | 2 | TxV 1 | 845.5 (746.5–960.8) | 973.0 (860.3–1088.2) |  | 27.3% (24.1–29.0%) | 29.8% (26.5–31.5%) |
|  | 2 | TxV 2 | 182.7 (136.6–245.3) | 288.3 (213.4–367.4) |  | 5.8% (4.4–7.7%) | 8.8% (6.6–10.9%) |
| **PxV status quo,Screening 2050** | | |  |  |  |  |  |
|  | - | TxV 0 | REF | REF |  | REF | REF |
|  | 1 | TxV 1^*^ | 253.9 (205.8–302.4) | 297.7 (243.2–349.9) |  | 7.3% (6.0–8.1%) | 8.3% (6.8–9.2%) |
|  | 1 | TxV 1^#^ | 398.6 (320.9–468.9) | 428.6 (348.7–499.7) |  | 11.4% (9.2–12.7%) | 11.9% (9.6–13.3%) |
|  | 2 | TxV 1 | 818.3 (720.1–923.9) | 936.0 (823.2–1045.7) |  | 23.4% (20.5–24.9%) | 26.1% (23.1–27.8%) |
|  | 2 | TxV 2 | 169.2 (126.4–229.5) | 270.0 (198.0–345.3) |  | 4.8% (3.6–6.4%) | 7.5% (5.5–9.4%) |

The number of cervical cancer cases and deaths averted was calculated as the difference between outcomes in the TxV vaccination and no-vaccination scenarios. The reduction in cases or deaths was defined as the number of cases or deaths averted divided by the corresponding number in the scenario without TxV vaccination. Base case = two-dose prophylactic HPV vaccine coverage among 13-year-old girls increases linearly to 90% by 2030, while screening coverage remains unchanged.

Status quo = current prophylactic HPV vaccination or/and screening&treatment practices; PxV 2030/2050 = two-dose prophylactic HPV vaccine coverage among 13-year-old girls increases linearly to 90% by 2030/2050; Screening 2030/2050 = HPV DNA-based screening coverage increases linearly to 90% by 2030/2050; TxV0 = no therapeutic vaccination; Use Case 1 (TxV1*) = population-based vaccination with a type 1 therapeutic vaccine targeting clearance of oncogenic HPV infections, administered annually to women aged 30 years; Use Case 1 (TxV1#) = same as TxV1*, with an initial catch-up campaign for women aged 31-45 years in the first year of roll-out; Use Case 2 (TxV1) = screening-based vaccination with a type 1 therapeutic vaccine targeting HPV-positive, triage-negative women; Use Case 2 (TxV2) = screening-based vaccination with a type 2 therapeutic vaccine targeting women diagnosed with CIN2/3 through HPV-based screening.

**Table S11.** Estimated cervical cancer deaths across different scenarios in China (thousands)

| **Scenario** | **Use case** | **Type of TxV** | **Cervical cancer deaths** | | | | |
| --- | --- | --- | --- | --- | --- | --- | --- |
|  |  |  | **2025-2050** | **2051-2075** | **2076-2100** | **2101-2125** | **2025-2125** |
| **Base case (PxV 2030, Screening status quo)** | | |  |  |  |  |  |
|  | - | TxV 0 | 1257.2 (1233.6–1298.2) | 1078.6 (1049.3–1132.6) | 639.0 (621.6–672.9) | 326.1 (314.2–348.4) | 3300.4 (3220.2–3452.1) |
|  | 1 | TxV 1^*^ | 1251.7 (1228.1–1291.9) | 1049.7 (1020.3–1102.9) | 621.0 (603.0–651.5) | 325.4 (313.3–347.0) | 3245.6 (3166.4–3393.1) |
|  | 1 | TxV 1^#^ | 1225.8 (1202.1–1264.2) | 988.9 (955.6–1039.6) | 582.2 (561.0–613.7) | 325.0 (313.0–346.6) | 3125.4 (3034.8–3261.7) |
|  | 2 | TxV 1 | 1226.1 (1202.1–1264.9) | 862.5 (825.2–914.4) | 470.4 (447.5–501.2) | 321.6 (309.8–342.4) | 2884.8 (2789.7–3023.3) |
|  | 2 | TxV 2 | 1238.4 (1215.9–1277.1) | 1043.8 (1016.2–1096.5) | 627.3 (610.8–660.5) | 325.9 (314.0–348.1) | 3234.3 (3160.4–3376.6) |
| **PxV 2050,Screening status quo** | | |  |  |  |  |  |
|  | - | TxV 0 | 1258.2 (1234.7–1299.3) | 1109.1 (1079.4–1164.9) | 695.9 (677.2–730.8) | 375.3 (363.0–397.4) | 3438.4 (3354.9–3592.8) |
|  | 1 | TxV 1^*^ | 1252.7 (1229.1–1292.9) | 1069.0 (1038.5–1121.5) | 662.6 (641.9–695.2) | 363.9 (350.8–384.7) | 3347.3 (3262.6–3497.4) |
|  | 1 | TxV 1^#^ | 1226.8 (1203.1–1265.2) | 1007.1 (973.2–1060.4) | 622.4 (598.7–656.9) | 362.1 (349.1–382.8) | 3222.3 (3124.5–3366.0) |
|  | 2 | TxV 1 | 1227.2 (1203.2–1265.9) | 882.5 (843.4–936.1) | 490.6 (465.6–526.7) | 336.1 (322.7–357.3) | 2937.8 (2839.1–3084.5) |
|  | 2 | TxV 2 | 1239.5 (1217.0–1278.2) | 1072.8 (1043.9–1127.7) | 679.7 (662.0–713.7) | 373.3 (361.0–395.2) | 3364.5 (3285.4–3511.5) |
| **PxV 2030,Screening 2030** | | |  |  |  |  |  |
|  | - | TxV 0 | 1128.2 (1100.3–1177.8) | 748.0 (717.2–805.6) | 436.1 (419.9–464.0) | 187.2 (176.9–204.6) | 2498.3 (2416.6–2648.6) |
|  | 1 | TxV 1^*^ | 1124.2 (1096.2–1173.3) | 734.3 (704.1–788.7) | 423.1 (406.9–451.1) | 186.4 (176.2–203.4) | 2466.4 (2387.8–2615.9) |
|  | 1 | TxV 1^#^ | 1107.4 (1080.2–1153.6) | 698.9 (667.3–749.9) | 390.4 (372.2–417.2) | 186.0 (176.0–203.2) | 2383.5 (2298.8–2523.4) |
|  | 2 | TxV 1 | 1094.8 (1066.5–1141.3) | 521.0 (486.1–569.9) | 250.1 (232.6–275.5) | 181.8 (172.0–198.7) | 2051.0 (1961.1–2184.9) |
|  | 2 | TxV 2 | 1103.7 (1077.4–1150.8) | 708.4 (679.0–765.9) | 423.4 (408.5–451.6) | 186.9 (176.8–204.3) | 2422.1 (2343.2–2564.8) |
| **PxV 2050,Screening 2050** | | |  |  |  |  |  |
|  | - | TxV 0 | 1288.0 (1262.3–1332.0) | 925.9 (892.3–990.7) | 481.5 (463.4–515.8) | 230.8 (220.3–249.0) | 2926.8 (2838.1–3087.0) |
|  | 1 | TxV 1^*^ | 1283.1 (1257.4–1326.1) | 900.5 (866.7–961.8) | 461.9 (442.8–493.5) | 220.9 (209.9–239.2) | 2865.4 (2781.3–3024.2) |
|  | 1 | TxV 1^#^ | 1258.5 (1232.3–1299.2) | 855.0 (819.3–913.5) | 427.8 (405.9–460.3) | 219.6 (208.5–238.1) | 2761.1 (2668.2–2910.2) |
|  | 2 | TxV 1 | 1258.5 (1232.2–1300.3) | 717.4 (676.5–776.7) | 283.2 (262.1–314.3) | 188.0 (177.0–205.6) | 2448.1 (2351.3–2593.0) |
|  | 2 | TxV 2 | 1267.5 (1243.4–1310.7) | 884.3 (851.6–949.0) | 464.6 (446.6–497.8) | 228.7 (218.4–247.0) | 2844.9 (2762.1–3001.1) |
| **PxV status quo, Screening status quo** | | |  |  |  |  |  |
|  | - | TxV 0 | 1259.0 (1235.4–1300.1) | 1151.2 (1120.4–1209.2) | 885.4 (860.9–930.6) | 756.0 (734.5–794.2) | 4051.5 (3951.7–4234.7) |
|  | 1 | TxV 1^*^ | 1253.3 (1229.8–1293.6) | 1098.5 (1066.3–1153.1) | 799.1 (770.6–840.1) | 663.6 (636.7–699.3) | 3818.6 (3708.6–3984.3) |
|  | 1 | TxV 1^#^ | 1227.5 (1203.8–1265.9) | 1036.2 (1000.4–1091.8) | 757.4 (724.8–800.0) | 660.6 (633.8–696.3) | 3683.7 (3567.1–3857.3) |
|  | 2 | TxV 1 | 1228.0 (1203.9–1266.6) | 914.5 (873.9–969.8) | 595.6 (562.5–640.3) | 501.9 (474.0–541.0) | 3243.2 (3116.5–3411.9) |
|  | 2 | TxV 2 | 1240.2 (1217.7–1278.9) | 1111.8 (1082.2–1169.5) | 854.3 (830.7–899.0) | 728.8 (709.1–767.5) | 3934.2 (3841.1–4114.4) |
| **PxV status quo,Screening 2030** | | |  |  |  |  |  |
|  | - | TxV 0 | 1130.0 (1102.1–1179.5) | 794.2 (760.9–857.0) | 568.5 (544.2–612.1) | 478.6 (456.6–514.9) | 2971.9 (2864.2–3163.8) |
|  | 1 | TxV 1^*^ | 1125.8 (1098.0–1174.9) | 767.0 (734.5–824.9) | 523.4 (497.6–561.0) | 426.2 (402.9–459.4) | 2841.4 (2737.0–3019.0) |
|  | 1 | TxV 1^#^ | 1109.0 (1081.9–1155.2) | 731.0 (697.1–785.0) | 489.1 (461.3–527.6) | 423.8 (400.6–456.5) | 2753.6 (2643.1–2924.6) |
|  | 2 | TxV 1 | 1096.5 (1068.3–1143.1) | 550.5 (513.3–601.8) | 296.6 (272.3–331.6) | 243.8 (224.2–271.7) | 2188.8 (2079.6–2345.7) |
|  | 2 | TxV 2 | 1105.5 (1079.1–1152.7) | 747.1 (715.5–811.1) | 530.6 (507.6–575.2) | 445.1 (425.1–482.7) | 2828.0 (2730.1–3023.5) |
| **PxV status quo,Screening 2050** | | |  |  |  |  |  |
|  | - | TxV 0 | 1288.8 (1263.0–1332.8) | 956.7 (921.5–1024.4) | 584.2 (558.7–631.9) | 478.6 (456.6–515.1) | 3308.6 (3200.1–3502.0) |
|  | 1 | TxV 1^*^ | 1283.8 (1258.1–1326.8) | 923.1 (887.5–986.3) | 538.3 (512.2–579.0) | 426.2 (402.9–459.6) | 3169.2 (3063.7–3348.8) |
|  | 1 | TxV 1^#^ | 1259.2 (1233.0–1299.9) | 876.6 (839.5–937.4) | 503.4 (474.5–545.9) | 423.9 (400.6–456.7) | 3067.1 (2950.8–3238.6) |
|  | 2 | TxV 1 | 1259.2 (1233.0–1301.1) | 739.3 (697.2–800.5) | 326.0 (299.1–365.3) | 246.2 (226.0–274.4) | 2570.9 (2458.2–2733.6) |
|  | 2 | TxV 2 | 1268.2 (1244.2–1311.5) | 911.1 (877.0–978.4) | 546.2 (522.2–594.5) | 445.1 (425.1–482.8) | 3170.7 (3072.5–3367.0) |

Base case = two-dose prophylactic HPV vaccine coverage among 13-year-old girls increases linearly to 90% by 2030, while screening coverage remains unchanged.

Status quo = current prophylactic HPV vaccination or/and screening&treatment practices; PxV 2030/2050 = two-dose prophylactic HPV vaccine coverage among 13-year-old girls increases linearly to 90% by 2030/2050; Screening 2030/2050 = HPV DNA-based screening coverage increases linearly to 90% by 2030/2050; TxV0 = no therapeutic vaccination; Use Case 1 (TxV1*) = population-based vaccination with a type 1 therapeutic vaccine targeting clearance of oncogenic HPV infections, administered annually to women aged 30 years; Use Case 1 (TxV1#) = same as TxV1*, with an initial catch-up campaign for women aged 31-45 years in the first year of roll-out; Use Case 2 (TxV1) = screening-based vaccination with a type 1 therapeutic vaccine targeting HPV-positive, triage-negative women; Use Case 2 (TxV2) = screening-based vaccination with a type 2 therapeutic vaccine targeting women diagnosed with CIN2/3 through HPV-based screening.

**Table S12.** Cervical cancer deaths averted by therapeutic HPV vaccination and percentage reduction across different scenarios and age groups (thousands)

| **Scenario** | **Use case** | **Type of TxV** | **Deaths of cervical cancer averted by TxV and percentage reduction** | | | | |
| --- | --- | --- | --- | --- | --- | --- | --- |
|  |  |  | **0-34 years** | **35-44 years** | **45-54 years** | **55-64 years** | **65+ years** |
| **Base case (PxV 2030, Screening status quo)** | | |  |  |  |  |  |
|  | 1 | TxV 1^*^ | 0.2 (0.4%) | 8.5 (18%) | 15.6 (33.1%) | 9.4 (20%) | 13.4 (28.5%) |
|  | 1 | TxV 1^#^ | 0.2 (0.2%) | 11.1 (7.9%) | 35.8 (25.5%) | 32.7 (23.3%) | 60.3 (43%) |
|  | 2 | TxV 1 | 0.1 (0%) | 4.8 (1.8%) | 34 (12.7%) | 51 (19.1%) | 177.8 (66.4%) |
|  | 2 | TxV 2 | 0.2 (0.4%) | 2.4 (4.8%) | 10.4 (21%) | 13.2 (26.7%) | 23.3 (47.2%) |
| **PxV 2050,Screening status quo** | | |  |  |  |  |  |
|  | 1 | TxV 1^*^ | 0.5 (0.6%) | 12.5 (16.1%) | 24.4 (31.3%) | 15.9 (20.4%) | 24.7 (31.7%) |
|  | 1 | TxV 1^#^ | 0.6 (0.3%) | 15.3 (8.8%) | 45 (26%) | 39.6 (22.9%) | 72.8 (42%) |
|  | 2 | TxV 1 | 0.2 (0.1%) | 6.9 (2.1%) | 44.5 (13.7%) | 63.4 (19.5%) | 210.6 (64.7%) |
|  | 2 | TxV 2 | 0.3 (0.5%) | 3 (5.2%) | 12.3 (21.7%) | 15.2 (26.8%) | 26 (45.8%) |
| **PxV 2030,Screening 2030** | | |  |  |  |  |  |
|  | 1 | TxV 1^*^ | 0.2 (0.7%) | 5.2 (21.8%) | 7.7 (32.5%) | 3.1 (13.1%) | 7.6 (31.9%) |
|  | 1 | TxV 1^#^ | 0.2 (0.3%) | 7.1 (8.9%) | 21.6 (27.1%) | 13.4 (16.8%) | 37.4 (46.9%) |
|  | 2 | TxV 1 | 0.1 (0%) | 4.6 (1.9%) | 27.6 (11.2%) | 30.3 (12.3%) | 183.4 (74.5%) |
|  | 2 | TxV 2 | 0.2 (0.3%) | 3.1 (5.1%) | 14 (23%) | 16.3 (26.7%) | 27.4 (44.9%) |
| **PxV 2050,Screening 2050** | | |  |  |  |  |  |
|  | 1 | TxV 1^*^ | 0.5 (1%) | 9.4 (19.6%) | 16.5 (34.3%) | 6.8 (14.1%) | 14.9 (31%) |
|  | 1 | TxV 1^#^ | 0.6 (0.5%) | 11.9 (9.7%) | 35.9 (29.3%) | 23.9 (19.5%) | 50.3 (41%) |
|  | 2 | TxV 1 | 0.2 (0.1%) | 6.8 (2.4%) | 38.5 (13.8%) | 40.6 (14.5%) | 193.5 (69.2%) |
|  | 2 | TxV 2 | 0.3 (0.4%) | 3.4 (5.2%) | 14.8 (22.9%) | 17.7 (27.4%) | 28.5 (44.1%) |
| **PxV status quo, Screening status quo** | | |  |  |  |  |  |
|  | 1 | TxV 1^*^ | 4.8 (2.2%) | 43.9 (19.9%) | 75.9 (34.5%) | 43.5 (19.8%) | 52 (23.6%) |
|  | 1 | TxV 1^#^ | 5.1 (1.6%) | 46.9 (14.8%) | 96.9 (30.6%) | 67.6 (21.3%) | 100.7 (31.7%) |
|  | 2 | TxV 1 | 2.1 (0.4%) | 22.3 (3.8%) | 107.6 (18.6%) | 125.5 (21.7%) | 322.3 (55.6%) |
|  | 2 | TxV 2 | 0.9 (0.9%) | 7.8 (8.1%) | 25.3 (26.2%) | 26.1 (27.1%) | 36.2 (37.6%) |
| **PxV status quo,Screening 2030** | | |  |  |  |  |  |
|  | 1 | TxV 1^*^ | 4.8 (4.3%) | 26.2 (23.7%) | 36.8 (33.3%) | 14.4 (13%) | 28.4 (25.7%) |
|  | 1 | TxV 1^#^ | 5.1 (3%) | 28.4 (16.8%) | 51 (30.2%) | 25 (14.8%) | 59.3 (35.1%) |
|  | 2 | TxV 1 | 2.7 (0.6%) | 19.8 (4.1%) | 83.8 (17.5%) | 69 (14.4%) | 304.3 (63.4%) |
|  | 2 | TxV 2 | 0.9 (0.7%) | 11.2 (9.2%) | 35.5 (29.2%) | 31.6 (25.9%) | 42.5 (34.9%) |
| **PxV status quo,Screening 2050** | | |  |  |  |  |  |
|  | 1 | TxV 1^*^ | 4.8 (4%) | 28.4 (23.8%) | 41.7 (34.9%) | 15.9 (13.3%) | 28.8 (24.1%) |
|  | 1 | TxV 1^#^ | 5.1 (2.6%) | 31.1 (15.9%) | 61.3 (31.4%) | 33.1 (16.9%) | 64.6 (33.1%) |
|  | 2 | TxV 1 | 2.5 (0.5%) | 20.2 (4.3%) | 86.9 (18.5%) | 73 (15.6%) | 286 (61%) |
|  | 2 | TxV 2 | 0.9 (0.8%) | 10.4 (9%) | 33.4 (28.8%) | 30.6 (26.4%) | 40.6 (35%) |
|  |  |  |  |  |  |  |  |

Base case = two-dose prophylactic HPV vaccine coverage among 13-year-old girls increases linearly to 90% by 2030, while screening coverage remains unchanged.

Status quo = current prophylactic HPV vaccination or/and screening&treatment practices; PxV 2030/2050 = two-dose prophylactic HPV vaccine coverage among 13-year-old girls increases linearly to 90% by 2030/2050; Screening 2030/2050 = HPV DNA-based screening coverage increases linearly to 90% by 2030/2050; TxV0 = no therapeutic vaccination; Use Case 1 (TxV1*) = population-based vaccination with a type 1 therapeutic vaccine targeting clearance of oncogenic HPV infections, administered annually to women aged 30 years; Use Case 1 (TxV1#) = same as TxV1*, with an initial catch-up campaign for women aged 31-45 years in the first year of roll-out; Use Case 2 (TxV1) = screening-based vaccination with a type 1 therapeutic vaccine targeting HPV-positive, triage-negative women; Use Case 2 (TxV2) = screening-based vaccination with a type 2 therapeutic vaccine targeting women diagnosed with CIN2/3 through HPV-based screening.

**Table S13.** Estimated cervical cancer deaths across different scenarios in urban areas in China (thousands)

| **Scenario** | **Use case** | **Type of TxV** | **Cervical cancer deaths** | | | | |
| --- | --- | --- | --- | --- | --- | --- | --- |
|  |  |  | **2025-2050** | **2051-2075** | **2076-2100** | **2101-2125** | **2025-2125** |
| **Base case (PxV 2030, Screening status quo)** | | |  |  |  |  |  |
|  | - | TxV 0 | 657.0 (642.0–682.6) | 583.2 (565.6–618.1) | 319.2 (309.1–338.7) | 148.1 (141.8–159.5) | 1706.7 (1659.2–1799.0) |
|  | 1 | TxV 1^*^ | 654.2 (639.3–679.6) | 569.2 (551.2–602.0) | 310.1 (299.8–328.7) | 147.6 (141.4–159.1) | 1681.2 (1632.6–1769.4) |
|  | 1 | TxV 1^#^ | 639.0 (624.3–663.8) | 538.1 (519.0–571.0) | 289.2 (277.4–307.7) | 147.3 (141.2–158.9) | 1613.3 (1564.0–1701.9) |
|  | 2 | TxV 1 | 638.0 (623.1–662.9) | 465.7 (443.2–497.0) | 231.8 (219.0–249.5) | 145.6 (139.6–157.3) | 1481.3 (1426.0–1565.9) |
|  | 2 | TxV 2 | 647.5 (633.3–672.8) | 567.0 (550.8–601.0) | 313.7 (304.2–333.0) | 148.0 (141.7–159.4) | 1676.5 (1631.1–1765.9) |
| **PxV 2050,Screening status quo** | | |  |  |  |  |  |
|  | - | TxV 0 | 657.4 (642.5–683.1) | 599.9 (581.0–635.8) | 345.9 (334.8–367.0) | 173.4 (167.0–186.1) | 1775.8 (1725.6–1871.7) |
|  | 1 | TxV 1^*^ | 654.7 (639.7–680.0) | 579.2 (560.9–612.3) | 329.1 (318.3–348.9) | 167.3 (160.7–179.2) | 1729.8 (1680.2–1818.9) |
|  | 1 | TxV 1^#^ | 639.5 (624.7–664.2) | 548.4 (528.1–581.8) | 308.1 (295.2–327.2) | 166.5 (159.8–178.3) | 1662.5 (1609.3–1754.5) |
|  | 2 | TxV 1 | 638.5 (623.6–663.4) | 475.9 (452.5–508.1) | 240.4 (227.0–259.7) | 152.6 (145.9–164.4) | 1508.5 (1450.2–1594.3) |
|  | 2 | TxV 2 | 648.0 (633.8–673.3) | 582.2 (565.3–617.2) | 338.4 (328.3–358.9) | 172.4 (166.2–184.9) | 1741.4 (1693.7–1832.6) |
| **PxV 2030,Screening 2030** | | |  |  |  |  |  |
|  | - | TxV 0 | 593.3 (576.2–623.8) | 419.7 (401.7–455.3) | 225.3 (217.3–241.3) | 88.3 (83.7–97.4) | 1327.0 (1278.9–1417.6) |
|  | 1 | TxV 1^*^ | 591.4 (574.3–621.8) | 412.7 (395.5–446.4) | 218.8 (210.8–233.6) | 88.0 (83.3–96.9) | 1311.1 (1264.6–1399.7) |
|  | 1 | TxV 1^#^ | 580.8 (564.4–610.8) | 393.2 (376.2–424.8) | 200.7 (190.9–215.8) | 87.8 (83.1–96.7) | 1262.0 (1216.4–1346.9) |
|  | 2 | TxV 1 | 574.2 (557.1–604.0) | 293.2 (272.6–323.9) | 128.5 (119.0–142.2) | 85.6 (81.1–94.1) | 1082.8 (1030.6–1161.9) |
|  | 2 | TxV 2 | 581.5 (565.5–612.1) | 401.9 (385.8–437.2) | 219.9 (212.4–235.2) | 88.2 (83.6–97.2) | 1292.0 (1248.9–1380.3) |
| **PxV 2050,Screening 2050** | | |  |  |  |  |  |
|  | - | TxV 0 | 682.6 (666.3–710.6) | 519.9 (498.7–559.9) | 248.4 (238.5–267.9) | 111.2 (106.3–120.7) | 1562.0 (1510.1–1659.7) |
|  | 1 | TxV 1^*^ | 680.0 (664.1–708.4) | 506.2 (485.8–542.8) | 238.3 (229.0–256.5) | 106.0 (100.9–115.3) | 1530.8 (1480.3–1621.0) |
|  | 1 | TxV 1^#^ | 665.4 (649.4–692.4) | 481.3 (461.0–519.2) | 219.7 (208.5–237.5) | 105.2 (100.1–114.5) | 1471.3 (1420.0–1566.5) |
|  | 2 | TxV 1 | 665.2 (648.9–692.3) | 403.4 (379.1–437.9) | 146.3 (134.8–162.9) | 88.7 (83.6–97.8) | 1304.5 (1247.5–1391.0) |
|  | 2 | TxV 2 | 672.4 (657.2–700.2) | 500.6 (481.9–540.7) | 241.0 (232.2–260.1) | 110.3 (105.5–119.8) | 1524.3 (1477.5–1620.3) |
| **PxV status quo, Screening status quo** | | |  |  |  |  |  |
|  | - | TxV 0 | 657.8 (642.8–683.4) | 620.8 (601.2–657.6) | 431.0 (416.9–457.5) | 339.2 (328.1–360.2) | 2048.2 (1989.7–2158.4) |
|  | 1 | TxV 1^*^ | 655.0 (640.0–680.4) | 593.6 (574.5–627.8) | 389.8 (374.8–413.7) | 297.5 (284.6–315.8) | 1934.5 (1875.5–2041.3) |
|  | 1 | TxV 1^#^ | 639.9 (625.0–664.5) | 562.4 (541.3–596.2) | 367.9 (350.7–391.7) | 296.0 (283.0–314.5) | 1867.0 (1801.2–1966.2) |
|  | 2 | TxV 1 | 638.9 (624.0–663.7) | 491.1 (467.2–524.4) | 285.8 (268.9–311.9) | 221.1 (208.1–241.5) | 1641.2 (1569.3–1736.8) |
|  | 2 | TxV 2 | 648.3 (634.1–673.6) | 601.5 (584.2–637.9) | 417.1 (404.9–443.1) | 328.4 (318.6–349.0) | 1996.8 (1941.9–2102.3) |
| **PxV status quo,Screening 2030** | | |  |  |  |  |  |
|  | - | TxV 0 | 594.1 (576.9–624.7) | 443.2 (423.2–481.6) | 286.4 (273.6–310.3) | 222.9 (212.5–241.6) | 1545.8 (1486.5–1659.2) |
|  | 1 | TxV 1^*^ | 592.1 (575.0–622.6) | 428.6 (410.3–463.7) | 263.9 (251.9–285.7) | 198.1 (187.6–215.2) | 1482.1 (1426.2–1585.8) |
|  | 1 | TxV 1^#^ | 581.5 (565.1–611.6) | 408.7 (390.7–443.5) | 245.4 (231.7–266.7) | 197.0 (186.3–213.9) | 1433.0 (1375.0–1538.3) |
|  | 2 | TxV 1 | 574.9 (557.8–604.8) | 307.2 (285.5–340.1) | 149.1 (136.8–167.6) | 113.1 (103.9–126.9) | 1147.3 (1085.0–1234.3) |
|  | 2 | TxV 2 | 582.3 (566.3–612.9) | 421.7 (404.4–459.6) | 271.1 (259.9–295.3) | 210.6 (201.7–229.9) | 1486.5 (1431.5–1596.3) |
| **PxV status quo,Screening 2050** | | |  |  |  |  |  |
|  | - | TxV 0 | 682.9 (666.7–711.0) | 534.9 (512.8–576.3) | 295.1 (281.4–321.4) | 222.9 (212.5–241.7) | 1735.2 (1673.8–1852.4) |
|  | 1 | TxV 1^*^ | 680.4 (664.4–708.7) | 516.6 (495.5–554.4) | 272.4 (259.8–296.1) | 198.1 (187.6–215.2) | 1667.3 (1608.1–1772.4) |
|  | 1 | TxV 1^#^ | 665.6 (649.7–692.7) | 492.2 (470.7–530.5) | 253.6 (239.2–275.6) | 197.1 (186.3–213.9) | 1608.2 (1547.3–1718.0) |
|  | 2 | TxV 1 | 665.5 (649.3–692.7) | 413.8 (388.8–449.7) | 165.5 (151.4–186.2) | 114.2 (104.9–128.1) | 1360.5 (1296.1–1454.5) |
|  | 2 | TxV 2 | 672.7 (657.5–700.5) | 514.0 (494.5–555.7) | 280.0 (268.0–305.3) | 210.6 (201.7–230.0) | 1677.2 (1621.7–1791.8) |

Base case = two-dose prophylactic HPV vaccine coverage among 13-year-old girls increases linearly to 90% by 2030, while screening coverage remains unchanged.

Status quo = current prophylactic HPV vaccination or/and screening&treatment practices; PxV 2030/2050 = two-dose prophylactic HPV vaccine coverage among 13-year-old girls increases linearly to 90% by 2030/2050; Screening 2030/2050 = HPV DNA-based screening coverage increases linearly to 90% by 2030/2050; TxV0 = no therapeutic vaccination; Use Case 1 (TxV1*) = population-based vaccination with a type 1 therapeutic vaccine targeting clearance of oncogenic HPV infections, administered annually to women aged 30 years; Use Case 1 (TxV1#) = same as TxV1*, with an initial catch-up campaign for women aged 31-45 years in the first year of roll-out; Use Case 2 (TxV1) = screening-based vaccination with a type 1 therapeutic vaccine targeting HPV-positive, triage-negative women; Use Case 2 (TxV2) = screening-based vaccination with a type 2 therapeutic vaccine targeting women diagnosed with CIN2/3 through HPV-based screening.

**Table S14.** Estimated cervical cancer deaths across different scenarios in rural areas in China (thousands)

| **Scenario** | **Use case** | **Type of TxV** | **Cervical cancer deaths** | | | | |
| --- | --- | --- | --- | --- | --- | --- | --- |
|  |  |  | **2025-2050** | **2051-2075** | **2076-2100** | **2101-2125** | **2025-2125** |
| **Base case (PxV 2030, Screening status quo)** | | |  |  |  |  |  |
|  | - | TxV 0 | 601.2 (589.2–618.4) | 495.2 (480.8–517.7) | 320.0 (310.5–334.8) | 178.1 (171.3–188.8) | 1594.2 (1552.6–1658.8) |
|  | 1 | TxV 1^*^ | 598.0 (585.9–615.0) | 480.8 (465.9–501.6) | 310.6 (300.9–324.4) | 177.7 (171.0–188.4) | 1566.9 (1524.6–1628.8) |
|  | 1 | TxV 1^#^ | 586.7 (574.5–602.5) | 449.8 (434.0–471.2) | 292.6 (281.9–307.0) | 177.5 (170.9–188.2) | 1506.6 (1462.1–1567.5) |
|  | 2 | TxV 1 | 588.0 (575.8–604.2) | 397.9 (380.3–419.7) | 239.7 (227.6–254.4) | 176.0 (169.4–186.7) | 1400.3 (1355.2–1462.0) |
|  | 2 | TxV 2 | 591.3 (580.6–607.3) | 477.5 (464.4–498.3) | 314.1 (305.2–328.1) | 177.9 (171.2–188.7) | 1561.7 (1521.8–1619.8) |
| **PxV 2050,Screening status quo** | | |  |  |  |  |  |
|  | - | TxV 0 | 601.8 (589.8–618.9) | 509.6 (494.7–533.3) | 350.4 (340.2–366.5) | 202.1 (195.1–213.1) | 1663.2 (1619.7–1731.8) |
|  | 1 | TxV 1^*^ | 598.5 (586.4–615.5) | 489.9 (474.8–511.3) | 333.1 (322.4–347.8) | 196.6 (189.1–207.0) | 1618.0 (1573.4–1679.6) |
|  | 1 | TxV 1^#^ | 587.3 (575.0–603.2) | 458.8 (442.3–480.3) | 314.5 (302.1–330.7) | 195.8 (188.3–206.2) | 1555.5 (1509.8–1619.4) |
|  | 2 | TxV 1 | 588.5 (576.3–604.8) | 407.3 (389.1–429.8) | 250.6 (237.4–267.3) | 183.5 (176.0–194.0) | 1429.6 (1380.1–1493.4) |
|  | 2 | TxV 2 | 591.9 (581.2–607.8) | 490.6 (477.2–512.8) | 341.9 (332.5–356.6) | 201.1 (194.3–212.0) | 1626.4 (1585.2–1687.4) |
| **PxV 2030,Screening 2030** | | |  |  |  |  |  |
|  | - | TxV 0 | 535.7 (521.2–557.4) | 328.2 (312.2–354.1) | 210.3 (201.4–224.2) | 98.7 (92.6–108.1) | 1173.7 (1128.7–1243.3) |
|  | 1 | TxV 1^*^ | 533.6 (518.9–554.9) | 321.9 (305.9–346.1) | 204.3 (195.3–217.8) | 98.3 (92.3–107.7) | 1158.0 (1113.0–1225.9) |
|  | 1 | TxV 1^#^ | 526.4 (512.0–546.3) | 304.4 (289.2–327.2) | 189.6 (179.9–203.0) | 98.2 (92.1–107.6) | 1119.0 (1074.4–1183.8) |
|  | 2 | TxV 1 | 520.7 (506.1–540.2) | 228.1 (211.8–249.9) | 121.7 (113.0–134.8) | 96.3 (90.3–105.8) | 967.4 (923.9–1027.5) |
|  | 2 | TxV 2 | 522.1 (509.5–542.5) | 306.6 (292.2–330.2) | 203.8 (195.5–216.8) | 98.5 (92.6–108.0) | 1132.3 (1090.6–1195.6) |
| **PxV 2050,Screening 2050** | | |  |  |  |  |  |
|  | - | TxV 0 | 606.0 (593.1–624.4) | 407.2 (389.4–436.2) | 233.3 (222.9–249.8) | 119.8 (113.3–129.7) | 1366.0 (1318.9–1440.0) |
|  | 1 | TxV 1^*^ | 603.3 (590.3–621.3) | 395.1 (377.8–421.2) | 223.3 (213.0–238.9) | 115.1 (108.5–124.9) | 1336.0 (1290.3–1405.8) |
|  | 1 | TxV 1^#^ | 592.9 (580.0–609.8) | 373.1 (355.8–397.2) | 207.8 (196.5–223.0) | 114.5 (107.8–124.4) | 1288.8 (1241.1–1354.0) |
|  | 2 | TxV 1 | 593.1 (580.0–610.1) | 313.6 (295.1–338.5) | 137.0 (126.2–151.9) | 99.2 (92.8–108.9) | 1142.8 (1095.6–1207.6) |
|  | 2 | TxV 2 | 595.2 (583.7–612.5) | 384.4 (368.9–411.0) | 223.2 (213.8–239.1) | 118.6 (112.4–128.5) | 1322.3 (1279.0–1388.0) |
| **PxV status quo, Screening status quo** | | |  |  |  |  |  |
|  | - | TxV 0 | 602.2 (590.2–619.3) | 531.0 (515.5–555.4) | 455.0 (441.4–476.2) | 417.4 (404.7–437.1) | 2005.0 (1951.5–2088.2) |
|  | 1 | TxV 1^*^ | 598.9 (586.8–615.9) | 504.9 (489.3–527.3) | 408.4 (393.8–428.6) | 366.4 (351.5–385.5) | 1879.0 (1823.6–1953.7) |
|  | 1 | TxV 1^#^ | 587.6 (575.4–603.6) | 473.6 (456.3–496.4) | 389.8 (372.6–410.8) | 365.0 (349.9–384.1) | 1814.7 (1755.0–1893.4) |
|  | 2 | TxV 1 | 589.0 (576.7–605.2) | 424.1 (405.1–447.6) | 310.1 (292.7–332.7) | 281.1 (265.5–302.1) | 1605.0 (1541.3–1684.5) |
|  | 2 | TxV 2 | 592.3 (581.6–608.2) | 510.2 (496.5–533.1) | 437.1 (424.8–457.2) | 401.0 (389.3–419.5) | 1941.2 (1892.0–2017.7) |
| **PxV status quo,Screening 2030** | | |  |  |  |  |  |
|  | - | TxV 0 | 536.8 (522.2–558.4) | 351.6 (333.8–379.7) | 282.5 (267.9–304.6) | 255.8 (242.3–276.4) | 1428.3 (1365.9–1519.5) |
|  | 1 | TxV 1^*^ | 534.6 (519.9–555.9) | 338.2 (321.4–364.0) | 258.4 (244.6–277.9) | 227.6 (213.9–245.4) | 1359.9 (1301.8–1444.1) |
|  | 1 | TxV 1^#^ | 527.4 (512.9–547.3) | 320.6 (304.2–344.5) | 242.9 (228.4–263.1) | 226.6 (212.9–244.6) | 1318.2 (1260.3–1397.5) |
|  | 2 | TxV 1 | 521.6 (507.0–541.2) | 243.2 (225.7–266.1) | 146.9 (134.4–164.7) | 130.8 (119.8–146.9) | 1042.8 (988.8–1117.6) |
|  | 2 | TxV 2 | 523.1 (510.5–543.5) | 325.6 (310.2–351.2) | 259.6 (247.1–280.9) | 234.7 (222.9–254.4) | 1344.5 (1291.5–1429.7) |
| **PxV status quo,Screening 2050** | | |  |  |  |  |  |
|  | - | TxV 0 | 606.4 (593.5–624.8) | 422.8 (404.3–453.1) | 289.6 (274.5–313.0) | 255.8 (242.3–276.4) | 1575.5 (1514.4–1667.5) |
|  | 1 | TxV 1^*^ | 603.7 (590.7–621.7) | 406.5 (388.5–433.6) | 265.3 (250.9–285.9) | 227.6 (213.9–245.4) | 1503.5 (1446.2–1586.7) |
|  | 1 | TxV 1^#^ | 593.2 (580.4–610.1) | 384.4 (366.4–409.2) | 249.2 (234.4–270.6) | 226.6 (212.9–244.6) | 1454.7 (1395.9–1532.4) |
|  | 2 | TxV 1 | 593.5 (580.4–610.5) | 325.1 (305.9–351.1) | 160.5 (146.6–179.4) | 131.8 (120.7–148.1) | 1211.4 (1154.3–1288.7) |
|  | 2 | TxV 2 | 595.6 (584.1–613.0) | 397.9 (381.6–425.5) | 266.7 (253.6–288.7) | 234.7 (223.0–254.5) | 1495.9 (1442.5–1581.1) |

Base case = two-dose prophylactic HPV vaccine coverage among 13-year-old girls increases linearly to 90% by 2030, while screening coverage remains unchanged.

Status quo = current prophylactic HPV vaccination or/and screening&treatment practices; PxV 2030/2050 = two-dose prophylactic HPV vaccine coverage among 13-year-old girls increases linearly to 90% by 2030/2050; Screening 2030/2050 = HPV DNA-based screening coverage increases linearly to 90% by 2030/2050; TxV0 = no therapeutic vaccination; Use Case 1 (TxV1*) = population-based vaccination with a type 1 therapeutic vaccine targeting clearance of oncogenic HPV infections, administered annually to women aged 30 years; Use Case 1 (TxV1#) = same as TxV1*, with an initial catch-up campaign for women aged 31-45 years in the first year of roll-out; Use Case 2 (TxV1) = screening-based vaccination with a type 1 therapeutic vaccine targeting HPV-positive, triage-negative women; Use Case 2 (TxV2) = screening-based vaccination with a type 2 therapeutic vaccine targeting women diagnosed with CIN2/3 through HPV-based screening.

**Table S15.** Estimated cervical cancer deaths averted by therapeutic vaccines across different scenarios in urban and rural areas in China (thousands)

| **Scenario** | **Use case** | **Type of TxV** | **Cervical cancer deaths** | | | | |  | **Deaths of cervical cancer averted by TxV (Reduction of cases vs TxV0)** | | | | | | | | | |
| --- | --- | --- | --- | --- | --- | --- | --- | --- | --- | --- | --- | --- | --- | --- | --- | --- | --- | --- |
|  |  |  | **2025-**  **2050** | **2051-**  **2075** | **2076-**  **2100** | **2101-**  **2125** | **2025-**  **2125** |  | **2025-**  **2050** | | **2051-**  **2075** | | **2076-**  **2100** | | **2101-**  **2125** | | **2025-**  **2125** | |
| **Base case (PxV 2030, Screening status quo)** | | |  |  |  |  |  |  |  | |  | |  | |  | |  | |
|  | - | TxV 0 | REF | REF | REF | REF | REF |  | REF | | REF | | REF | | REF | | REF | |
|  | 1 | TxV 1^*^ | 6.1 (5.1–6.8) | 30.2 (24.6–33.8) | 19.5 (15.5–21.9) | 0.8 (0.6–1.6) | 56.8 (46.0–63.7) |  | 0.5% (0.4–0.5%) | | 2.8% (2.3–3.1%) | | 3.0% (2.4–3.4%) | | 0.3% (0.2–0.5%) | | 1.7% (1.4–1.9%) | |
|  | 1 | TxV 1^#^ | 33.2 (27.0–37.3) | 94.4 (76.2–106.9) | 60.0 (47.1–68.1) | 1.2 (0.8–2.1) | 189.2 (151.4–213.7) |  | 2.6% (2.2–2.9%) | | 8.7% (7.1–9.8%) | | 9.3% (7.4–10.6%) | | 0.4% (0.2–0.6%) | | 5.7% (4.6–6.4%) | |
|  | 2 | TxV 1 | 32.7 (27.3–36.3) | 221.9 (191.0–242.6) | 172.6 (152.6–184.4) | 4.1 (3.0–6.6) | 431.9 (374.6–468.3) |  | 2.6% (2.2–2.9%) | | 20.5% (17.6–22.3%) | | 26.8% (23.8–28.6%) | | 1.3% (0.9–2.0%) | | 13.0% (11.4–14.1%) | |
|  | 2 | TxV 2 | 18.9 (14.5–23.6) | 33.6 (25.9–41.8) | 11.4 (8.8–14.1) | 0.2 (0.1–0.3) | 64.0 (49.3–79.7) |  | 1.5% (1.1–1.9%) | | 3.1% (2.4–3.8%) | | 1.8% (1.4–2.2%) | | 0.1% (0.0–0.1%) | | 1.9% (1.5–2.4%) | |
| **PxV 2050,**  **Screening status quo** | | |  |  |  |  |  |  |  | |  | |  | |  | |  | |
|  | - | TxV 0 | REF | REF | REF | REF | REF |  | REF | | REF | | REF | | REF | | REF | |
|  | 1 | TxV 1^*^ | 6.2 (5.1–6.9) | 41.9 (34.7–47.1) | 35.4 (28.4–39.9) | 12.3 (9.8–13.8) | 96.0 (77.8–107.6) |  | 0.5% (0.4–0.5%) | | 3.8% (3.1–4.2%) | | 5.1% (4.1–5.6%) | | 3.2% (2.6–3.6%) | | 2.8% (2.3–3.1%) | |
|  | 1 | TxV 1^#^ | 33.3 (27.1–37.4) | 106.8 (86.6–120.4) | 77.3 (61.0–87.4) | 14.0 (11.0–15.8) | 231.2 (185.7–260.7) |  | 2.6% (2.2–2.9%) | | 9.6% (7.8–10.7%) | | 11.0% (8.8–12.4%) | | 3.7% (2.9–4.2%) | | 6.7% (5.4–7.5%) | |
|  | 2 | TxV 1 | 32.7 (27.3–36.4) | 233.5 (201.0–255.0) | 210.0 (186.0–224.9) | 40.6 (36.6–42.9) | 517.0 (450.9–559.9) |  | 2.6% (2.2–2.9%) | | 21.0% (17.9–22.8%) | | 29.9% (26.7–32.0%) | | 10.7% (9.6–11.4%) | | 15.0% (13.0–16.1%) | |
|  | 2 | TxV 2 | 18.9 (14.5–23.6) | 36.2 (27.8–44.8) | 15.6 (12.0–19.4) | 2.0 (1.5–2.4) | 72.7 (55.9–90.1) |  | 1.5% (1.1–1.9%) | | 3.2% (2.5–4.0%) | | 2.2% (1.7–2.8%) | | 0.5% (0.4–0.6%) | | 2.1% (1.6–2.6%) | |
| **PxV 2030,**  **Screening 2030** | | |  |  |  |  |  |  |  | |  | |  | |  | |  | |
|  | - | TxV 0 | REF | REF | REF | REF | REF |  | REF | | REF | | REF | | REF | | REF | |
|  | 1 | TxV 1^*^ | 4.1 (3.4–4.7) | 13.9 (11.3–16.7) | 13.2 (10.4–15.0) | 0.7 (0.5–1.3) | 32.0 (25.6–37.3) |  | 0.4% (0.3–0.4%) | | 1.9% (1.5–2.1%) | | 3.0% (2.4–3.4%) | | 0.4% (0.3–0.7%) | | 1.3% (1.0–1.4%) | |
|  | 1 | TxV 1^#^ | 22.2 (18.2–26.1) | 51.9 (42.0–60.9) | 47.8 (37.3–54.4) | 1.0 (0.7–1.8) | 123.3 (97.8–142.3) |  | 2.0% (1.6–2.3%) | | 7.0% (5.6–7.8%) | | 10.9% (8.6–12.3%) | | 0.5% (0.4–0.9%) | | 4.9% (3.9–5.6%) | |
|  | 2 | TxV 1 | 35.4 (29.2–40.1) | 232.0 (201.4–256.0) | 188.8 (172.4–199.0) | 4.7 (3.5–7.4) | 461.6 (407.8–500.5) |  | 3.1% (2.6–3.5%) | | 30.9% (26.9–33.5%) | | 43.0% (39.3–45.2%) | | 2.5% (1.9–4.0%) | | 18.4% (16.3–19.6%) | |
|  | 2 | TxV 2 | 24.6 (18.3–31.4) | 39.5 (30.2–48.8) | 12.1 (9.3–14.8) | 0.2 (0.1–0.3) | 76.3 (58.1–95.5) |  | 2.2% (1.6–2.8%) | | 5.2% (4.0–6.5%) | | 2.8% (2.1–3.4%) | | 0.1% (0.1–0.2%) | | 3.0% (2.3–3.8%) | |
| **PxV 2050,**  **Screening 2050** | | |  |  |  |  |  |  |  | |  | |  | |  | |  | |
|  | - | TxV 0 | REF | REF | REF | REF | REF |  | | REF | | REF | | REF | | REF | | REF |
|  | 1 | TxV 1^*^ | 5.3 (4.4–6.0) | 26.9 (22.2–31.6) | 21.0 (16.6–24.2) | 10.4 (8.3–11.7) | 63.7 (51.5–73.3) |  | 0.4% (0.3–0.5%) | | 2.9% (2.4–3.3%) | | 4.3% (3.5–4.9%) | | 4.5% (3.6–5.0%) | | 2.2% (1.8–2.4%) | |
|  | 1 | TxV 1^#^ | 31.2 (25.5–35.5) | 75.1 (60.8–86.8) | 57.3 (44.8–65.5) | 11.9 (9.4–13.5) | 175.7 (140.3–201.4) |  | 2.4% (2.0–2.7%) | | 8.1% (6.6–9.1%) | | 11.8% (9.3–13.4%) | | 5.1% (4.0–5.8%) | | 6.0% (4.8–6.7%) | |
|  | 2 | TxV 1 | 31.2 (25.8–35.2) | 217.1 (185.1–239.7) | 202.2 (182.6–216.3) | 44.1 (41.1–45.9) | 495.2 (434.9–536.3) |  | 2.4% (2.0–2.7%) | | 23.2% (19.8–25.4%) | | 41.7% (37.5–44.1%) | | 18.9% (17.4–20.0%) | | 16.8% (14.8–18.1%) | |
|  | 2 | TxV 2 | 20.2 (14.9–25.9) | 41.4 (30.9–52.2) | 17.5 (13.3–21.5) | 2.0 (1.6–2.5) | 81.0 (60.7–102.3) |  | 1.6% (1.1–2.0%) | | 4.4% (3.3–5.6%) | | 3.6% (2.8–4.4%) | | 0.9% (0.7–1.1%) | | 2.8% (2.1–3.5%) | |
| **PxV status quo, Screening status quo** | | |  |  |  |  |  |  |  | |  | |  | |  | |  | |
|  | - | TxV 0 | REF | REF | REF | REF | REF |  | REF | | REF | | REF | | REF | | REF | |
|  | 1 | TxV 1^*^ | 6.2 (5.1–6.9) | 55.1 (45.6–61.8) | 91.5 (73.9–103.2) | 97.0 (77.0–109.8) | 249.9 (201.3–281.7) |  | 0.5% (0.4–0.5%) | | 4.8% (4.0–5.3%) | | 10.3% (8.3–11.4%) | | 12.8% (10.2–14.3%) | | 6.1% (5.0–6.8%) | |
|  | 1 | TxV 1^#^ | 33.3 (27.1–37.4) | 120.3 (97.7–135.4) | 133.9 (107.1–151.2) | 100.0 (79.2–113.3) | 387.6 (310.8–437.2) |  | 2.6% (2.2–3.0%) | | 10.4% (8.5–11.6%) | | 15.0% (12.1–16.8%) | | 13.1% (10.5–14.7%) | | 9.5% (7.7–10.6%) | |
|  | 2 | TxV 1 | 32.8 (27.3–36.4) | 243.8 (209.8–266.4) | 295.8 (260.9–318.9) | 259.1 (229.5–278.5) | 831.9 (727.5–900.0) |  | 2.6% (2.2–2.9%) | | 21.1% (18.1–22.9%) | | 33.2% (29.3–35.5%) | | 34.1% (30.2–36.3%) | | 20.5% (17.8–22.0%) | |
|  | 2 | TxV 2 | 18.9 (14.5–23.6) | 39.1 (30.1–48.5) | 31.4 (24.1–38.7) | 26.9 (20.8–33.1) | 116.4 (89.4–144.6) |  | 1.5% (1.1–1.9%) | | 3.4% (2.6–4.2%) | | 3.5% (2.7–4.3%) | | 3.5% (2.8–4.3%) | | 2.9% (2.2–3.5%) | |
| **PxV status quo,**  **Screening 2030** | | |  |  |  |  |  |  |  | |  | |  | |  | |  | |
|  | - | TxV 0 | REF | REF | REF | REF | REF |  | REF | | REF | | REF | | REF | | REF | |
|  | 1 | TxV 1^*^ | 4.2 (3.5–4.8) | 28.2 (23.2–33.7) | 48.1 (38.7–57.0) | 55.3 (43.4–65.3) | 135.4 (108.7–161.2) |  | 0.4% (0.3–0.4%) | | 3.5% (2.9–4.0%) | | 8.5% (6.9–9.6%) | | 11.6% (9.1–13.1%) | | 4.6% (3.7–5.2%) | |
|  | 1 | TxV 1^#^ | 22.3 (18.3–26.2) | 66.6 (53.9–78.5) | 83.6 (66.2–97.3) | 57.6 (44.8–67.9) | 230.2 (183.6–269.9) |  | 2.0% (1.6–2.3%) | | 8.4% (6.8–9.5%) | | 14.7% (11.7–16.5%) | | 12.0% (9.4–13.7%) | | 7.8% (6.2–8.8%) | |
|  | 2 | TxV 1 | 35.5 (29.2–40.2) | 249.4 (217.0–276.3) | 276.2 (251.5–299.3) | 236.9 (217.8–256.0) | 799.1 (717.3–873.8) |  | 3.1% (2.6–3.5%) | | 31.4% (27.2–33.9%) | | 48.5% (44.3–50.8%) | | 49.6% (45.7–51.6%) | | 26.9% (24.0–28.4%) | |
|  | 2 | TxV 2 | 24.6 (18.3–31.4) | 47.0 (36.2–58.4) | 37.8 (29.0–46.4) | 33.1 (25.5–40.4) | 142.6 (109.2–176.8) |  | 2.2% (1.6–2.8%) | | 5.9% (4.5–7.2%) | | 6.6% (5.1–8.0%) | | 6.8% (5.3–8.3%) | | 4.8% (3.7–5.9%) | |
| **PxV status quo,**  **Screening 2050** | | |  |  |  |  |  |  |  | |  | |  | |  | |  | |
|  | - | TxV 0 | REF | REF | REF | REF | REF |  | REF | | REF | | REF | | REF | | REF | |
|  | 1 | TxV 1^*^ | 5.3 (4.4–6.0) | 35.6 (29.4–41.6) | 48.5 (39.1–57.5) | 55.3 (43.4–65.3) | 145.0 (116.3–170.4) |  | 0.4% (0.3–0.5%) | | 3.7% (3.1–4.2%) | | 8.4% (6.7–9.4%) | | 11.6% (9.1–13.1%) | | 4.4% (3.5–5.0%) | |
|  | 1 | TxV 1^#^ | 31.3 (25.5–35.6) | 84.0 (68.1–97.2) | 85.0 (67.3–98.9) | 57.6 (44.8–67.9) | 258.1 (205.6–299.9) |  | 2.4% (2.0–2.7%) | | 8.8% (7.1–9.8%) | | 14.5% (11.6–16.3%) | | 12.0% (9.4–13.7%) | | 7.8% (6.2–8.8%) | |
|  | 2 | TxV 1 | 31.2 (25.8–35.2) | 226.0 (192.7–249.9) | 262.9 (236.6–286.9) | 235.0 (215.5–254.3) | 755.2 (671.6–824.4) |  | 2.4% (2.0–2.7%) | | 23.4% (19.9–25.6%) | | 44.8% (40.5–47.4%) | | 49.1% (45.2–51.2%) | | 22.8% (20.2–24.3%) | |
|  | 2 | TxV 2 | 20.2 (14.9–25.9) | 45.2 (33.8–57.1) | 37.9 (29.0–46.8) | 33.1 (25.5–40.4) | 136.6 (103.8–170.7) |  | 1.6% (1.1–2.0%) | | 4.7% (3.5–5.9%) | | 6.4% (5.0–7.9%) | | 6.8% (5.3–8.3%) | | 4.1% (3.1–5.1%) | |

The number of cervical cancer cases and deaths averted was calculated as the difference between outcomes in the TxV vaccination and no-vaccination scenarios. Base case = two-dose prophylactic HPV vaccine coverage among 13-year-old girls increases linearly to 90% by 2030, while screening coverage remains unchanged.

Status quo = current prophylactic HPV vaccination or/and screening&treatment practices; PxV 2030/2050 = two-dose prophylactic HPV vaccine coverage among 13-year-old girls increases linearly to 90% by 2030/2050; Screening 2030/2050 = HPV DNA-based screening coverage increases linearly to 90% by 2030/2050; TxV0 = no therapeutic vaccination; Use Case 1 (TxV1*) = population-based vaccination with a type 1 therapeutic vaccine targeting clearance of oncogenic HPV infections, administered annually to women aged 30 years; Use Case 1 (TxV1#) = same as TxV1*, with an initial catch-up campaign for women aged 31-45 years in the first year of roll-out; Use Case 2 (TxV1) = screening-based vaccination with a type 1 therapeutic vaccine targeting HPV-positive, triage-negative women; Use Case 2 (TxV2) = screening-based vaccination with a type 2 therapeutic vaccine targeting women diagnosed with CIN2/3 through HPV-based screening.

**Table S16.** Estimated cervical cancer deaths averted by tharapeutic vaccines across different scenarios in urban and rural areas in China, 2025-2125 (thousands)

| **Scenario** | **Use case** | **Type of TxV** | **Deaths of cervical cancer averted by TxV** | |  | **Reduction of death vs TxV0** | |
| --- | --- | --- | --- | --- | --- | --- | --- |
|  |  |  | **Urban** | **Rural** |  | **Urban** | **Rural** |
| **Base case (PxV 2030, Screening status quo)** | | |  |  |  |  |  |
|  | - | TxV 0 | REF | REF |  | REF | REF |
|  | 1 | TxV 1^*^ | 27.5 (22.4–31.1) | 29.2 (23.6–32.7) |  | 1.6% (1.3–1.8%) | 1.8% (1.5–2.0%) |
|  | 1 | TxV 1^#^ | 97.5 (78.8–110.7) | 91.2 (72.9–103.3) |  | 5.7% (4.6–6.4%) | 5.7% (4.6–6.4%) |
|  | 2 | TxV 1 | 233.2 (203.2–254.5) | 198.1 (171.3–214.4) |  | 13.6% (11.9–14.7%) | 12.4% (10.8–13.4%) |
|  | 2 | TxV 2 | 29.9 (22.7–39.6) | 32.8 (24.2–41.9) |  | 1.8% (1.3–2.3%) | 2.1% (1.5–2.6%) |
| **PxV 2050,Screening status quo** | | |  |  |  |  |  |
|  | - | TxV 0 | REF | REF |  | REF | REF |
|  | 1 | TxV 1^*^ | 47.1 (38.5–53.2) | 48.6 (39.5–54.3) |  | 2.7% (2.2–2.9%) | 2.9% (2.4–3.2%) |
|  | 1 | TxV 1^#^ | 118.8 (95.9–134.3) | 112.0 (90.0–126.3) |  | 6.7% (5.4–7.5%) | 6.7% (5.4–7.5%) |
|  | 2 | TxV 1 | 276.4 (241.2–300.1) | 239.4 (208.6–259.4) |  | 15.5% (13.5–16.7%) | 14.4% (12.6–15.5%) |
|  | 2 | TxV 2 | 33.6 (25.6–44.6) | 37.5 (27.8–47.9) |  | 1.9% (1.4–2.5%) | 2.3% (1.7–2.8%) |
| **PxV 2030,Screening 2030** | | |  |  |  |  |  |
|  | - | TxV 0 | REF | REF |  | REF | REF |
|  | 1 | TxV 1^*^ | 15.9 (12.8–18.9) | 16.0 (12.9–18.8) |  | 1.2% (1.0–1.4%) | 1.4% (1.1–1.5%) |
|  | 1 | TxV 1^#^ | 66.0 (52.6–76.6) | 57.0 (45.9–66.2) |  | 5.0% (4.0–5.6%) | 4.9% (3.9–5.5%) |
|  | 2 | TxV 1 | 251.3 (221.7–273.7) | 210.2 (186.2–228.5) |  | 18.9% (16.7–20.2%) | 17.9% (16.0–19.1%) |
|  | 2 | TxV 2 | 33.3 (24.9–45.1) | 41.2 (30.2–53.0) |  | 2.5% (1.9–3.3%) | 3.5% (2.6–4.4%) |
| **PxV 2050,Screening 2050** | | |  |  |  |  |  |
|  | - | TxV 0 | REF | REF |  | REF | REF |
|  | 1 | TxV 1^*^ | 32.4 (26.1–37.4) | 31.2 (25.6–36.0) |  | 2.1% (1.7–2.3%) | 2.3% (1.9–2.6%) |
|  | 1 | TxV 1^#^ | 94.0 (75.0–107.7) | 81.6 (65.8–93.5) |  | 6.0% (4.8–6.7%) | 6.0% (4.8–6.7%) |
|  | 2 | TxV 1 | 266.0 (232.6–289.6) | 228.2 (201.7–247.5) |  | 16.9% (14.8–18.3%) | 16.7% (14.7–17.9%) |
|  | 2 | TxV 2 | 35.4 (26.0–48.3) | 43.8 (31.3–56.6) |  | 2.3% (1.7–3.1%) | 3.2% (2.3–4.1%) |
| **PxV status quo, Screening status quo** | | |  |  |  |  |  |
|  | - | TxV 0 | REF | REF |  | REF | REF |
|  | 1 | TxV 1^*^ | 117.2 (95.2–132.7) | 132.4 (107.0–148.2) |  | 5.7% (4.7–6.3%) | 6.6% (5.3–7.3%) |
|  | 1 | TxV 1^#^ | 190.0 (153.3–215.4) | 196.9 (158.2–221.5) |  | 9.3% (7.5–10.3%) | 9.8% (7.9–10.9%) |
|  | 2 | TxV 1 | 421.4 (368.1–456.8) | 409.6 (355.6–442.5) |  | 20.5% (17.9–22.0%) | 20.4% (17.8–21.9%) |
|  | 2 | TxV 2 | 49.7 (38.0–66.0) | 64.0 (47.5–81.8) |  | 2.4% (1.8–3.2%) | 3.2% (2.4–4.0%) |
| **PxV status quo,Screening 2030** | | |  |  |  |  |  |
|  | - | TxV 0 | REF | REF |  | REF | REF |
|  | 1 | TxV 1^*^ | 65.3 (51.9–78.0) | 70.3 (57.0–83.2) |  | 4.2% (3.4–4.8%) | 5.0% (4.0–5.6%) |
|  | 1 | TxV 1^#^ | 117.1 (93.0–137.2) | 113.3 (91.1–132.1) |  | 7.6% (6.1–8.6%) | 8.0% (6.3–9.0%) |
|  | 2 | TxV 1 | 408.7 (365.8–448.4) | 389.3 (348.8–425.2) |  | 26.5% (23.7–28.1%) | 27.4% (24.5–28.9%) |
|  | 2 | TxV 2 | 57.5 (43.1–77.2) | 82.0 (60.7–104.9) |  | 3.7% (2.8–4.9%) | 5.7% (4.3–7.2%) |
| **PxV status quo,Screening 2050** | | |  |  |  |  |  |
|  | - | TxV 0 | REF | REF |  | REF | REF |
|  | 1 | TxV 1^*^ | 70.2 (55.9–83.0) | 74.6 (60.4–87.7) |  | 4.1% (3.3–4.6%) | 4.8% (3.8–5.4%) |
|  | 1 | TxV 1^#^ | 132.3 (105.4–153.9) | 125.8 (101.8–145.8) |  | 7.6% (6.1–8.6%) | 8.0% (6.4–9.0%) |
|  | 2 | TxV 1 | 384.9 (341.0–423.1) | 368.6 (329.2–403.3) |  | 22.2% (19.6–23.7%) | 23.5% (20.9–25.0%) |
|  | 2 | TxV 2 | 55.1 (41.0–74.5) | 78.5 (57.2–100.7) |  | 3.2% (2.4–4.2%) | 5.0% (3.7–6.3%) |

The number of cervical cancer cases and deaths averted was calculated as the difference between outcomes in the TxV vaccination and no-vaccination scenarios. The reduction in cases or deaths was defined as the number of cases or deaths averted divided by the corresponding number in the scenario without TxV vaccination. Base case = two-dose prophylactic HPV vaccine coverage among 13-year-old girls increases linearly to 90% by 2030, while screening coverage remains unchanged.

Status quo = current prophylactic HPV vaccination or/and screening&treatment practices; PxV 2030/2050 = two-dose prophylactic HPV vaccine coverage among 13-year-old girls increases linearly to 90% by 2030/2050; Screening 2030/2050 = HPV DNA-based screening coverage increases linearly to 90% by 2030/2050; TxV0 = no therapeutic vaccination; Use Case 1 (TxV1*) = population-based vaccination with a type 1 therapeutic vaccine targeting clearance of oncogenic HPV infections, administered annually to women aged 30 years; Use Case 1 (TxV1#) = same as TxV1*, with an initial catch-up campaign for women aged 31-45 years in the first year of roll-out; Use Case 2 (TxV1) = screening-based vaccination with a type 1 therapeutic vaccine targeting HPV-positive, triage-negative women; Use Case 2 (TxV2) = screening-based vaccination with a type 2 therapeutic vaccine targeting women diagnosed with CIN2/3 through HPV-based screening.

**Table S17.** Estimated cervical cancer cases and cases averted by tharapeutic vaccines for sensitivity analysis in China (thousands)

| **Scenario** | **Use case** | **Type of TxV** | **Cervical cancer cases** | | | | |  | **Cases of cervical cancer averted by TxV (Reduction of cases vs TxV0)** | | | | |
| --- | --- | --- | --- | --- | --- | --- | --- | --- | --- | --- | --- | --- | --- |
|  |  |  | **2025-**  **2050** | **2051-**  **2075** | **2076-**  **2100** | **2101-**  **2125** | **2025-**  **2125** |  | **2025-**  **2050** | **2051-**  **2075** | **2076-**  **2100** | **2101-**  **2125** | **2025-**  **2125** |
| **Base case** | | |  |  |  |  |  |  |  |  |  |  |  |
|  | 0 | TxV 0 | 2956.5 | 1899.9 | 962.6 | 595.3 | 6414.4 |  | REF | REF | REF | REF | REF |
|  | 1 | TxV 1^*^ | 2916.3 | 1800.0 | 939.5 | 594.6 | 6250.3 |  | 40.2 (0.0%) | 100.0 (0.0%) | 23.1 (0.0%) | 0.7 (0.0%) | 164.0 (0.0%) |
|  | 1 | TxV 1^#^ | 2788.4 | 1671.7 | 902.1 | 594.3 | 5956.5 |  | 168.1 (0.0%) | 228.2 (0.0%) | 60.5 (0.0%) | 1.0 (0.0%) | 457.8 (0.0%) |
|  | 2 | TxV 1 | 2793.6 | 1435.4 | 779.3 | 591.7 | 5600 |  | 162.9 (0.0%) | 464.6 (0.0%) | 183.2 (0.0%) | 3.6 (0.0%) | 814.3 (0.0%) |
|  | 2 | TxV 2 | 2880.9 | 1828.3 | 951.2 | 595.2 | 6255.6 |  | 75.6 (0.0%) | 71.6 (0.0%) | 11.4 (0.0%) | 0.1 (0.0%) | 158.8 (0.0%) |
| **Vaccine efficacy of TxV: 70%/50%** | | | | | | | | | | | | | |
|  | 1 | TxV 1^*^ | 2924.8 | 1821.8 | 945.2 | 594.8 | 6286.6 |  | 31.8 (-21.1%) | 78.1 (-21.9%) | 17.4 (-24.9%) | 0.5 (-25.9%) | 127.8 (-22.1%) |
|  | 1 | TxV 1^#^ | 2825.8 | 1724.5 | 917.8 | 594.6 | 6062.7 |  | 130.7 (-22.3%) | 175.4 (-23.1%) | 44.8 (-26.0%) | 0.7 (-27.4%) | 351.6 (-23.2%) |
|  | 2 | TxV 1 | 2825.5 | 1511.8 | 805.2 | 592.2 | 5734.7 |  | 131.1 (-19.6%) | 388.1 (-16.5%) | 157.4 (-14.1%) | 3.1 (-12.9%) | 679.7 (-16.5%) |
|  | 2 | TxV 2 | 2898 | 1844.3 | 953.7 | 595.2 | 6291.2 |  | 58.5 (-22.6%) | 55.7 (-22.2%) | 8.9 (-22.4%) | 0.1 (-22.5%) | 123.2 (-22.4%) |
| **Vaccine efficacy of TxV: 50%/0%** | | | | | | | | | | | | | |
|  | 1 | TxV 1^*^ | 2936.5 | 1851.4 | 952.4 | 595.0 | 6335.3 |  | 20.0 (-50.3%) | 48.5 (-51.5%) | 10.2 (-55.8%) | 0.3 (-57.2%) | 79.0 (-51.8%) |
|  | 1 | TxV 1^#^ | 2876.1 | 1793.4 | 936.7 | 594.9 | 6201.1 |  | 80.4 (-52.1%) | 106.5 (-53.3%) | 25.9 (-57.2%) | 0.4 (-59.2%) | 213.3 (-53.4%) |
|  | 2 | TxV 1 | 2872.3 | 1636.8 | 851.5 | 593.1 | 5953.6 |  | 84.2 (-48.3%) | 263.2 (-43.3%) | 111.1 (-39.4%) | 2.3 (-37.1%) | 460.8 (-43.4%) |
|  | 2 | TxV 2 | 2921.3 | 1866.3 | 957.2 | 595.3 | 6340 |  | 35.2 (-53.4%) | 33.7 (-53.0%) | 5.3 (-53.1%) | 0.1 (-53.3%) | 74.3 (-53.2%) |
| **Coverage of TxV: 50%/50%** | | |  |  |  |  |  |  |  |  |  |  |  |
|  | 1 | TxV 1^*^ | 2924.7 | 1821.8 | 945.2 | 594.8 | 6286.6 |  | 31.8 (-21.1%) | 78.1 (-21.8%) | 17.4 (-24.9%) | 0.5 (-25.9%) | 127.8 (-22.1%) |
|  | 1 | TxV 1^#^ | 2830.1 | 1728.3 | 918.4 | 594.6 | 6071.4 |  | 126.5 (-24.8%) | 171.7 (-24.8%) | 44.1 (-27.1%) | 0.7 (-27.6%) | 343.0 (-25.1%) |
|  | 2 | TxV 1 | 2835.1 | 1538.3 | 815.5 | 592.4 | 5781.2 |  | 121.5 (-25.5%) | 361.6 (-22.2%) | 147.1 (-19.7%) | 3.0 (-17.7%) | 633.2 (-22.2%) |
|  | 2 | TxV 2 | 2911.5 | 1857 | 955.8 | 595.2 | 6319.5 |  | 45.0 (-40.5%) | 43.0 (-40.0%) | 6.8 (-40.2%) | 0.1 (-40.3%) | 94.9 (-40.2%) |
| **Coverage of TxV: 100%/100%** | | |  |  |  |  |  |  |  |  |  |  |  |
|  | 1 | TxV 1^*^ | 2911.9 | 1788.6 | 936.3 | 594.5 | 6231.3 |  | 44.6 (10.8%) | 111.4 (11.4%) | 26.3 (13.8%) | 0.8 (14.5%) | 183.1 (11.6%) |
|  | 1 | TxV 1^#^ | 2767.7 | 1642.5 | 893 | 594.2 | 5897.4 |  | 188.8 (12.3%) | 257.5 (12.8%) | 69.6 (15.0%) | 1.2 (15.9%) | 517.0 (12.9%) |
|  | 2 | TxV 1 | 2775.7 | 1395.1 | 766.4 | 591.5 | 5528.7 |  | 180.8 (11.0%) | 504.9 (8.7%) | 196.1 (7.0%) | 3.8 (6.1%) | 885.6 (8.8%) |
|  | 2 | TxV 2 | 2868.8 | 1817.1 | 949.4 | 595.2 | 6230.4 |  | 87.7 (16.0%) | 82.8 (15.7%) | 13.2 (15.8%) | 0.2 (15.9%) | 183.9 (15.8%) |
| **Roll-out year of TxV: 2027** | | |  |  |  |  |  |  |  |  |  |  |  |
|  | 1 | TxV 1^*^ | 2897.2 | 1783 | 935 | 594.6 | 6209.8 |  | 59.3 (47.3%) | 117.0 (17.0%) | 27.6 (19.5%) | 0.8 (7.8%) | 204.6 (24.7%) |
|  | 1 | TxV 1^#^ | 2736.7 | 1658 | 904.5 | 594.4 | 5893.5 |  | 219.8 (30.8%) | 242.0 (6.0%) | 58.1 (-4.0%) | 1.0 (-3.3%) | 520.9 (13.8%) |
|  | 2 | TxV 1 | 2723.1 | 1398.5 | 775.1 | 591.7 | 5488.5 |  | 233.4 (43.2%) | 501.4 (7.9%) | 187.4 (2.3%) | 3.6 (0.4%) | 925.9 (13.7%) |
|  | 2 | TxV 2 | 2863.2 | 1828 | 951.2 | 595.2 | 6237.5 |  | 93.3 (23.4%) | 71.9 (0.5%) | 11.4 (0.0%) | 0.1 (0.0%) | 176.8 (11.4%) |
| **Roll-out year of TxV: 2035** | | | | | | | | | | | | | |
|  | 1 | TxV 1^*^ | 2938.2 | 1831.2 | 947.6 | 594.8 | 6311.8 |  | 18.3 (-54.4%) | 68.7 (-31.3%) | 15.0 (-35.3%) | 0.6 (-22.2%) | 102.6 (-37.5%) |
|  | 1 | TxV 1^#^ | 2866.7 | 1702.9 | 900.3 | 594.3 | 6064.2 |  | 89.8 (-46.6%) | 197.0 (-13.7%) | 62.3 (3.0%) | 1.1 (5.8%) | 350.1 (-23.5%) |
|  | 2 | TxV 1 | 2882.6 | 1507.7 | 789.6 | 591.8 | 5771.7 |  | 73.9 (-54.7%) | 392.2 (-15.6%) | 173.0 (-5.6%) | 3.5 (-1.3%) | 642.7 (-21.1%) |
|  | 2 | TxV 2 | 2908.1 | 1829.4 | 951.2 | 595.2 | 6283.9 |  | 48.4 (-36.0%) | 70.5 (-1.5%) | 11.4 (-0.0%) | 0.1 (0.0%) | 130.5 (-17.8%) |
| **Targeted HPV types of TxV: HPV 16,18,52,58** | | | | | | | | | | | | | |
|  | 1 | TxV 1^*^ | 2910.8 | 1752.4 | 859.4 | 504.7 | 6027.4 |  | 45.7 (13.5%) | 147.5 (47.6%) | 103.2 (346.4%) | 90.6 (12644.1%) | 387.0 (135.9%) |
|  | 1 | TxV 1^#^ | 2760 | 1583.4 | 801.1 | 501.4 | 5645.9 |  | 196.5 (16.9%) | 316.6 (38.7%) | 161.5 (166.9%) | 93.9 (9345.4%) | 768.5 (67.9%) |
|  | 2 | TxV 1 | 2767 | 1294.5 | 605.9 | 432.7 | 5100.1 |  | 189.5 (16.3%) | 605.5 (30.3%) | 356.7 (94.6%) | 162.6 (4425.5%) | 1314.2 (61.4%) |
|  | 2 | TxV 2 | 2858.8 | 1788.5 | 916.7 | 564.7 | 6128.7 |  | 97.7 (29.2%) | 111.4 (55.6%) | 45.9 (302.5%) | 30.6 (20897.3%) | 285.6 (79.9%) |
| **Targeted HPV types of TxV: HPV 16,18,52,58,31,33,35,45** | | | | | | | | | | | | | |
|  | 1 | TxV 1^*^ | 2908.5 | 1722.2 | 787.2 | 403 | 5820.8 |  | 48.0 (19.3%) | 177.8 (77.8%) | 175.4 (659.0%) | 192.4 (26955.1%) | 593.6 (261.9%) |
|  | 1 | TxV 1^#^ | 2743.3 | 1521 | 706.2 | 392.4 | 5362.9 |  | 213.2 (26.9%) | 378.9 (66.0%) | 256.4 (323.6%) | 202.9 (20309.2%) | 1051.4 (129.7%) |
|  | 2 | TxV 1 | 2749 | 1193.6 | 472 | 300.5 | 4715.1 |  | 207.5 (27.4%) | 706.4 (52.0%) | 490.6 (167.8%) | 294.8 (8104.4%) | 1699.3 (108.7%) |
|  | 2 | TxV 2 | 2846.4 | 1766.6 | 897.8 | 548 | 6058.9 |  | 110.1 (45.6%) | 133.3 (86.2%) | 64.8 (468.1%) | 47.3 (32353.0%) | 355.5 (123.9%) |

The number of cervical cancer cases and deaths averted was calculated as the difference between outcomes in the TxV vaccination and no-vaccination scenarios. Base case=two-dose prophylactic HPV vaccine coverage among 13-year-old girls increases linearly to 90% by 2030, while screening coverage remains unchanged, and therapeutic vaccine with 90% of efficacy for two doses and 70% for one dose, 70% of coverage for two doses and 20% for one dose, roll-out year of 2030 and targeted HPV infection or precancerous lesions related with HPV 16/18; TxV efficacy 70/50= therapeutic vaccine with 70% of efficacy for two doses and 50% for one dose; TxV efficacy 50/0= therapeutic vaccine with 50% of efficacy for two doses and 0% for one dose; TxV coverage 100/0= therapeutic vaccine with 100% of coverage for two doses; TxV coverage 50/0= therapeutic vaccine with 50% of coverage for two doses and 0% for one dose; TxV targeted HPV 16/18/52/58= therapeutic vaccine targeted HPV infection or precancerous lesions related with HPV 16/18/52/58; TxV targeted HPV 16/18/52/58/31/33/35/45= therapeutic vaccine targeted HPV infection or precancerous lesions related with HPV 16/18/52/58/31/33/35/45. Use Case 1 (TxV1*) = population-based vaccination with a type 1 therapeutic vaccine targeting clearance of oncogenic HPV infections, administered annually to women aged 30 years; Use Case 1 (TxV1#) = same as TxV1*, with an initial catch-up campaign for women aged 31-45 years in the first year of roll-out; Use Case 2 (TxV1) = screening-based vaccination with a type 1 therapeutic vaccine targeting HPV-positive, triage-negative women; Use Case 2 (TxV2) = screening-based vaccination with a type 2 therapeutic vaccine targeting women diagnosed with CIN2/3 through HPV-based screening.

**Table S18.** Estimated cervical cancer deaths and deaths averted by tharapeutic vaccines for sensitivity analysis in China (thousands)

| **Scenario** | **Use case** | **Type of TxV** | **Cervical cancer deaths** | | | | |  | **Deaths of cervical cancer averted by TxV (Reduction of cases vs TxV0)** | | | | |
| --- | --- | --- | --- | --- | --- | --- | --- | --- | --- | --- | --- | --- | --- |
|  |  |  | **2025-**  **2050** | **2051-**  **2075** | **2076-**  **2100** | **2101-**  **2125/** | **2025-**  **2125** |  | **2025-**  **2050** | **2051-**  **2075** | **2076-**  **2100** | **2101-**  **2125** | **2025-**  **2125** |
| **Base case** | | |  |  |  |  |  |  |  |  |  |  |  |
|  | 0 | TxV 0 | 1254.5 | 1064 | 622.1 | 308.4 | 3249.1 |  | 0.0 (0.0%) | 0.0 (0.0%) | 0.0 (0.0%) | 0.0 (0.0%) | 0.0 (0.0%) |
|  | 1 | TxV 1^*^ | 1248.6 | 1034.7 | 603.2 | 307.5 | 3194.1 |  | 5.9 (0.0%) | 29.3 (0.0%) | 18.9 (0.0%) | 0.9 (0.0%) | 55.0 (0.0%) |
|  | 1 | TxV 1^#^ | 1222.4 | 972.7 | 564.2 | 307.2 | 3066.5 |  | 32.0 (0.0%) | 91.4 (0.0%) | 58.0 (0.0%) | 1.2 (0.0%) | 182.6 (0.0%) |
|  | 2 | TxV 1 | 1222.7 | 847.8 | 453.5 | 304.1 | 2828.1 |  | 31.8 (0.0%) | 216.2 (0.0%) | 168.6 (0.0%) | 4.3 (0.0%) | 420.9 (0.0%) |
|  | 2 | TxV 2 | 1235.7 | 1030.7 | 610.8 | 308.2 | 3185.5 |  | 18.8 (0.0%) | 33.3 (0.0%) | 11.3 (0.0%) | 0.2 (0.0%) | 63.5 (0.0%) |
| **Vaccine efficacy of TxV: 70%/50%** | | | | | | | | | | | | | |
|  | 1 | TxV 1^*^ | 1249.8 | 1041.1 | 607.9 | 307.7 | 3206.6 |  | 4.6 (-21.3%) | 23.0 (-21.7%) | 14.2 (-24.7%) | 0.7 (-25.7%) | 42.5 (-22.7%) |
|  | 1 | TxV 1^#^ | 1229.6 | 993.7 | 579.0 | 307.5 | 3109.8 |  | 24.9 (-22.3%) | 70.4 (-23.0%) | 43.1 (-25.6%) | 0.9 (-27.2%) | 139.3 (-23.7%) |
|  | 2 | TxV 1 | 1229.1 | 884.8 | 477.5 | 304.6 | 2896 |  | 25.4 (-20.1%) | 179.2 (-17.1%) | 144.6 (-14.2%) | 3.8 (-12.7%) | 353.0 (-16.1%) |
|  | 2 | TxV 2 | 1240 | 1038.2 | 613.4 | 308.3 | 3199.8 |  | 14.5 (-22.7%) | 25.9 (-22.4%) | 8.8 (-22.4%) | 0.2 (-22.5%) | 49.3 (-22.5%) |
| **Vaccine efficacy of TxV: 50%/0%** | | | | | | | | | | | | | |
|  | 1 | TxV 1^*^ | 1251.6 | 1049.7 | 613.7 | 308 | 3223.1 |  | 2.9 (-50.6%) | 14.3 (-51.2%) | 8.4 (-55.5%) | 0.4 (-56.9%) | 26.0 (-52.7%) |
|  | 1 | TxV 1^#^ | 1239.2 | 1021.3 | 597.0 | 307.9 | 3165.4 |  | 15.3 (-52.3%) | 42.8 (-53.2%) | 25.1 (-56.7%) | 0.5 (-58.8%) | 83.7 (-54.2%) |
|  | 2 | TxV 1 | 1238.3 | 943.9 | 520.2 | 305.7 | 3008.2 |  | 16.2 (-49.1%) | 120.1 (-44.5%) | 101.9 (-39.6%) | 2.7 (-36.8%) | 240.9 (-42.8%) |
|  | 2 | TxV 2 | 1245.8 | 1048.4 | 616.8 | 308.3 | 3219.4 |  | 8.7 (-53.6%) | 15.6 (-53.1%) | 5.3 (-53.1%) | 0.1 (-53.3%) | 29.7 (-53.3%) |
| **Coverage of TxV: 50%/50%** | | |  |  |  |  |  |  |  |  |  |  |  |
|  | 1 | TxV 1^*^ | 1249.8 | 1041.1 | 607.9 | 307.7 | 3206.6 |  | 4.6 (-21.3%) | 23.0 (-21.7%) | 14.2 (-24.7%) | 0.7 (-25.6%) | 42.5 (-22.7%) |
|  | 1 | TxV 1^#^ | 1230.5 | 996.0 | 579.8 | 307.5 | 3113.8 |  | 24.0 (-25.1%) | 68.0 (-25.6%) | 42.4 (-26.9%) | 0.9 (-27.3%) | 135.2 (-25.9%) |
|  | 2 | TxV 1 | 1231.2 | 900.4 | 487.2 | 304.9 | 2923.7 |  | 23.3 (-26.8%) | 163.6 (-24.3%) | 134.9 (-20.0%) | 3.6 (-17.5%) | 325.4 (-22.7%) |
|  | 2 | TxV 2 | 1243.3 | 1044.1 | 615.4 | 308.3 | 3211.1 |  | 11.1 (-40.6%) | 19.9 (-40.2%) | 6.7 (-40.2%) | 0.1 (-40.3%) | 37.9 (-40.3%) |
| **Coverage of TxV: 100%/100%** | | |  |  |  |  |  |  |  |  |  |  |  |
|  | 1 | TxV 1^*^ | 1247.9 | 1031.4 | 600.7 | 307.4 | 3187.4 |  | 6.5 (11.0%) | 32.6 (11.3%) | 21.4 (13.6%) | 1.0 (14.3%) | 61.6 (12.1%) |
|  | 1 | TxV 1^#^ | 1218.5 | 960.9 | 555.6 | 307 | 3041.9 |  | 36.0 (12.5%) | 103.2 (12.9%) | 66.5 (14.7%) | 1.4 (15.7%) | 207.1 (13.4%) |
|  | 2 | TxV 1 | 1219 | 827.5 | 441.5 | 303.8 | 2791.8 |  | 35.5 (11.5%) | 236.6 (9.4%) | 180.7 (7.1%) | 4.6 (6.0%) | 457.3 (8.6%) |
|  | 2 | TxV 2 | 1232.7 | 1025.5 | 609.1 | 308.2 | 3175.4 |  | 21.8 (16.1%) | 38.6 (15.8%) | 13.1 (15.8%) | 0.2 (15.9%) | 73.6 (15.9%) |
| **Roll-out year of TxV: 2027** | | |  |  |  |  |  |  |  |  |  |  |  |
|  | 1 | TxV 1^*^ | 1244.9 | 1028.7 | 598.9 | 307.5 | 3179.9 |  | 9.6 (62.7%) | 35.4 (20.7%) | 23.3 (23.3%) | 1.0 (7.4%) | 69.2 (25.9%) |
|  | 1 | TxV 1^#^ | 1209.8 | 960.1 | 566.4 | 307.2 | 3043.4 |  | 44.7 (39.6%) | 104.0 (13.8%) | 55.8 (-3.8%) | 1.2 (-3.1%) | 205.6 (12.6%) |
|  | 2 | TxV 1 | 1203.5 | 819.2 | 448.9 | 304.1 | 2775.7 |  | 51.0 (60.5%) | 244.8 (13.2%) | 173.2 (2.7%) | 4.3 (0.4%) | 473.4 (12.5%) |
|  | 2 | TxV 2 | 1229.9 | 1030.2 | 610.8 | 308.2 | 3179.2 |  | 24.5 (30.8%) | 33.8 (1.6%) | 11.3 (0.0%) | 0.2 (0.0%) | 69.9 (9.9%) |
| **Roll-out year of TxV: 2035** | | | | | | | | | | | | | |
|  | 1 | TxV 1^*^ | 1252.3 | 1044.8 | 610.5 | 307.7 | 3215.4 |  | 2.1 (-63.6%) | 19.2 (-34.4%) | 11.6 (-38.5%) | 0.7 (-21.4%) | 33.7 (-38.7%) |
|  | 1 | TxV 1^#^ | 1240.2 | 996.3 | 563.3 | 307.1 | 3106.9 |  | 14.3 (-55.4%) | 67.8 (-25.8%) | 58.8 (1.4%) | 1.3 (5.3%) | 142.1 (-22.2%) |
|  | 2 | TxV 1 | 1243.5 | 898.3 | 464.5 | 304.2 | 2910.4 |  | 11.0 (-65.3%) | 165.8 (-23.3%) | 157.7 (-6.5%) | 4.3 (-1.2%) | 338.7 (-19.5%) |
|  | 2 | TxV 2 | 1244.2 | 1032.3 | 610.9 | 308.2 | 3195.5 |  | 10.3 (-45.1%) | 31.8 (-4.7%) | 11.3 (-0.0%) | 0.2 (0.0%) | 53.5 (-15.7%) |
| **Targeted HPV types of TxV: HPV 16,18,52,58** | | | | | | | | | | | | | |
|  | 1 | TxV 1^*^ | 1247.8 | 1023.7 | 574.7 | 268.7 | 3114.9 |  | 6.6 (12.6%) | 40.3 (37.5%) | 47.5 (151.4%) | 39.7 (4318.6%) | 134.1 (143.9%) |
|  | 1 | TxV 1^#^ | 1217.3 | 942 | 517.9 | 266.4 | 2943.6 |  | 37.2 (16.1%) | 122.1 (33.6%) | 104.2 (79.7%) | 42.1 (3335.9%) | 305.5 (67.3%) |
|  | 2 | TxV 1 | 1217.7 | 786.9 | 361 | 219.3 | 2584.9 |  | 36.8 (15.6%) | 277.1 (28.2%) | 261.1 (54.8%) | 89.1 (1964.8%) | 664.1 (57.8%) |
|  | 2 | TxV 2 | 1229.8 | 1011.7 | 594.1 | 293.8 | 3129.4 |  | 24.7 (31.8%) | 52.4 (57.2%) | 28.0 (148.2%) | 14.6 (6896.9%) | 119.6 (88.2%) |
| **Targeted HPV types of TxV: HPV 16,18,52,58,31,33,35,45** | | | | | | | | | | | | | |
|  | 1 | TxV 1^*^ | 1247.5 | 1017 | 548.9 | 221.6 | 3035 |  | 7.0 (18.0%) | 47.0 (60.4%) | 73.2 (287.8%) | 86.8 (9562.1%) | 214.0 (289.2%) |
|  | 1 | TxV 1^#^ | 1214.3 | 920.8 | 475.2 | 214.6 | 2824.9 |  | 40.2 (25.6%) | 143.2 (56.7%) | 146.9 (153.5%) | 93.8 (7562.5%) | 424.2 (132.3%) |
|  | 2 | TxV 1 | 1214.3 | 742.8 | 290.4 | 149.5 | 2397 |  | 40.2 (26.4%) | 321.3 (48.6%) | 331.7 (96.7%) | 159.0 (3582.6%) | 852.1 (102.4%) |
|  | 2 | TxV 2 | 1226.3 | 1000.9 | 584.8 | 285.7 | 3097.7 |  | 28.2 (50.3%) | 63.2 (89.7%) | 37.4 (231.4%) | 22.7 (10786.3%) | 151.4 (138.2%) |

The number of cervical cancer cases and deaths averted was calculated as the difference between outcomes in the TxV vaccination and no-vaccination scenarios. Base case=two-dose prophylactic HPV vaccine coverage among 13-year-old girls increases linearly to 90% by 2030, while screening coverage remains unchanged, and therapeutic vaccine with 90% of efficacy for two doses and 70% for one dose, 70% of coverage for two doses and 20% for one dose, roll-out year of 2030 and targeted HPV infection or precancerous lesions related with HPV 16/18; TxV efficacy 70/50= therapeutic vaccine with 70% of efficacy for two doses and 50% for one dose; TxV efficacy 50/0= therapeutic vaccine with 50% of efficacy for two doses and 0% for one dose; TxV coverage 100/0= therapeutic vaccine with 100% of coverage for two doses; TxV coverage 50/0= therapeutic vaccine with 50% of coverage for two doses and 0% for one dose; TxV targeted HPV 16/18/52/58= therapeutic vaccine targeted HPV infection or precancerous lesions related with HPV 16/18/52/58; TxV targeted HPV 16/18/52/58/31/33/35/45= therapeutic vaccine targeted HPV infection or precancerous lesions related with HPV 16/18/52/58/31/33/35/45. Use Case 1 (TxV1*) = population-based vaccination with a type 1 therapeutic vaccine targeting clearance of oncogenic HPV infections, administered annually to women aged 30 years; Use Case 1 (TxV1#) = same as TxV1*, with an initial catch-up campaign for women aged 31-45 years in the first year of roll-out; Use Case 2 (TxV1) = screening-based vaccination with a type 1 therapeutic vaccine targeting HPV-positive, triage-negative women; Use Case 2 (TxV2) = screening-based vaccination with a type 2 therapeutic vaccine targeting women diagnosed with CIN2/3 through HPV-based screening.

**Table S19.** Number of therapeutic vaccine doses needed to avert one cervical cancer case (2025-2100)

| **Scenario** | **Use Case** | **Type of TxV** | **NNV^ǂ^** |
| --- | --- | --- | --- |
| **Base case (PxV 2030, Screening status quo)** | | |  |
|  | 1 | TxV 1^*^ | 4191 (3726-5144) |
|  | 1 | TxV 1^#^ | 1998 (1772-2482) |
|  | 2 | TxV 1 | 25 (23-29) |
|  | 2 | TxV 2 | 16 (13-20) |
| **PxV 2050, Screening status quo** | | |  |
|  | 1 | TxV 1^*^ | 2675 (2383-3258) |
|  | 1 | TxV 1^#^ | 1644 (1459-2035) |
|  | 2 | TxV 1 | 24 (22-28) |
|  | 2 | TxV 2 | 16 (13-21) |
| **PxV 2030, Screening 2030** | | |  |
|  | 1 | TxV 1^*^ | 6677 (5641-8225) |
|  | 1 | TxV 1^#^ | 2920 (2487-3633) |
|  | 2 | TxV 1 | 33 (30-37) |
|  | 2 | TxV 2 | 16 (13-21) |
| **PxV 2050, Screening 2050** | | |  |
|  | 1 | TxV 1^*^ | 3681 (3165-4492) |
|  | 1 | TxV 1^#^ | 2046 (1771-2531) |
|  | 2 | TxV 1 | 29 (26-34) |
|  | 2 | TxV 2 | 17 (13-23) |
| **PxV status quo, Screening status quo** | | |  |
|  | 1 | TxV 1^*^ | 1257 (1119-1534) |
|  | 1 | TxV 1^#^ | 1077 (958-1331) |
|  | 2 | TxV 1 | 27 (25-31) |
|  | 2 | TxV 2 | 20 (16-26) |
| **PxV status quo, Screening 2030** | | |  |
|  | 1 | TxV 1^*^ | 1986 (1673-2428) |
|  | 1 | TxV 1^#^ | 1634 (1383-2016) |
|  | 2 | TxV 1 | 36 (32-41) |
|  | 2 | TxV 2 | 20 (16-26) |
| **PxV status quo, Screening 2050** | | |  |
|  | 0 | TxV 0 | REF |
|  | 1 | TxV 1^*^ | 1830 (1558-2235) |
|  | 1 | TxV 1^#^ | 1435 (1232-1768) |
|  | 2 | TxV 1 | 35 (31-40) |
|  | 2 | TxV 2 | 20 (16-27) |

NNV^ǂ^ =The number of therapeutic vaccine doses needed to avert one cervical cancer case. NNV was was calculated by dividing the total number of doses administered under each scenario by the corresponding number of cervical cancer cases averted during 2025-2100.

Base case = two-dose prophylactic HPV vaccine coverage among 13-year-old girls increases linearly to 90% by 2030, while screening coverage remains unchanged. Status quo = current prophylactic HPV vaccination or/and screening&treatment practices; PxV 2030/2050 = two-dose prophylactic HPV vaccine coverage among 13-year-old girls increases linearly to 90% by 2030/2050; Screening 2030/2050 = HPV DNA-based screening coverage increases linearly to 90% by 2030/2050; TxV0 = no therapeutic vaccination; Use Case 1 (TxV1*) = population-based vaccination with a type 1 therapeutic vaccine targeting clearance of oncogenic HPV infections, administered annually to women aged 30 years; Use Case 1 (TxV1#) = same as TxV1*, with an initial catch-up campaign for women aged 31-45 years in the first year of roll-out; Use Case 2 (TxV1) = screening-based vaccination with a type 1 therapeutic vaccine targeting HPV-positive, triage-negative women; Use Case 2 (TxV2) = screening-based vaccination with a type 2 therapeutic vaccine targeting women diagnosed with CIN2/3 through HPV-based screening.

**Figure S11.** Sensitivity analysis of the effect of different assumptions about therapeutic vaccine characteristics on the percentage change in cervical cancer deaths averted compared with the base case

Bars show the relative change of cervical cancer deathsaverted compared with the base case scenario. Purple and blue bars represent Use Case 1 (TxV1) with and without catch-up vaccination, respectively. Yellow and green bars represent Use Case 2 (TxV1 and TxV2). The vertical dashed line indicates the base-case estimate. Base case=two-dose prophylactic HPV vaccine coverage among 13-year-old girls increases linearly to 90% by 2030, while screening coverage remains unchanged, and therapeutic vaccine with 90% of efficacy for two doses and 70% for one dose, 70% of coverage for two doses and 20% for one dose, roll-out year of 2030 and targeted HPV infection or precancerous lesions related with HPV 16/18; TxV efficacy 70/50= therapeutic vaccine with 70% of efficacy for two doses and 50% for one dose; TxV efficacy 50/0= therapeutic vaccine with 50% of efficacy for two doses and 0% for one dose; TxV coverage 100/0= therapeutic vaccine with 100% of coverage for two doses; TxV coverage 50/0= therapeutic vaccine with 50% of coverage for two doses and 0% for one dose; TxV targeted HPV 16/18/52/58= therapeutic vaccine targeted HPV infection or precancerous lesions related with HPV 16/18/52/58; TxV targeted HPV 16/18/52/58/31/33/35/45= therapeutic vaccine targeted HPV infection or precancerous lesions related with HPV 16/18/52/58/31/33/35/45. Use Case 1 (TxV1*) = population-based vaccination with a type 1 therapeutic vaccine targeting clearance of oncogenic HPV infections, administered annually to women aged 30 years; Use Case 1 (TxV1#) = same as TxV1*, with an initial catch-up campaign for women aged 31-45 years in the first year of roll-out; Use Case 2 (TxV1) = screening-based vaccination with a type 1 therapeutic vaccine targeting HPV-positive, triage-negative women; Use Case 2 (TxV2) = screening-based vaccination with a type 2 therapeutic vaccine targeting women diagnosed with CIN2/3 through HPV-based screening.

**Figure S12.** Sensitivity analysis of screening and treatment parameters influencing the cervical cancer deaths averted by TxV1 (A) and TxV2 (B) in Use Case 2, 2025–2125

Bars show the percentage change in the number of cervical cancer deaths averted compared with the base-case scenario when varying key screening and treatment parameters within their uncertainty ranges. Parameters include HPV-based screening sensitivity for CIN2 and CIN3 (blue-grey and red), treatment rates for CIN2 and CIN3 (green and turquoise), and treatment efficacy for CIN2 and CIN3 (purple and orange). Light-colored bars represent the lower bound of each parameter, and dark-colored bars represent the upper bound. The vertical dashed line indicates the base-case estimate. Positive values indicate increased cervical cancer cases averted, whereas negative values indicate reduced impact relative to the base case.

# References

1.	Xia C, Hu S, Xu X, et al. Projections up to 2100 and a budget optimisation strategy towards cervical cancer elimination in China: a modelling study. *Lancet Public Health* 2019; **4**(9): e462-e72.

2.	Choi HCW, Jit M, Leung GM, Tsui KL, Wu JT. Simultaneously characterizing the comparative economics of routine female adolescent nonavalent human papillomavirus (HPV) vaccination and assortativity of sexual mixing in Hong Kong Chinese: a modeling analysis. *BMC Med* 2018; **16**(1): 127.

3.	National Health Commission. Notice on issuance of action plan for accelerating cervical cancer elimination (2023-2030). <https://www.gov.cn/zhengce/zhengceku/2023-01/21/content_5738364.htm>; 2023. Accessed July 1, 2025.

4.	Gu X, Sun G, Zheng R, et al. Incidence and mortality of cervical cancer in China in 2015. *J Natl Cancer Cent* 2022; **2**(2): 70-7.

5.	Dong L, Hu S-Y, Zhang Q, et al. Risk Prediction of Cervical Cancer and Precancers by Type-Specific Human Papillomavirus: Evidence from a Population-Based Cohort Study in China. *Cancer Prev Res (Phila)* 2017; **10**(12): 745-51.

6.	Canfell K, Barnabas R, Patnick J, Beral V. The predicted effect of changes in cervical screening practice in the UK: results from a modelling study. *Br J Cancer* 2004; **91**(3): 530-6.

7.	Goldie SJ, Grima D, Kohli M, Wright TC, Weinstein M, Franco E. A comprehensive natural history model of HPV infection and cervical cancer to estimate the clinical impact of a prophylactic HPV-16/18 vaccine. *International Journal of Cancer* 2003; **106**(6): 896-904.

8.	Haeussler K, den Hout Av, Baio G. A dynamic Bayesian Markov model for health economic evaluations of interventions in infectious disease. *BMC Med Res Methodol* 2018; **18**(1): 82.

9.	Liu YJ, Zhang Q, Hu SY, Zhao FH. Effect of vaccination age on cost-effectiveness of human papillomavirus vaccination against cervical cancer in China. *BMC Cancer* 2016; **16**: 164.

10.	Haeussler K, Marcellusi A, Mennini FS, et al. Cost-Effectiveness Analysis of Universal Human Papillomavirus Vaccination Using a Dynamic Bayesian Methodology: The BEST II Study. *Value Health* 2015; **18**(8): 956-68.

11.	Myers ER, McCrory DC, Nanda K, Bastian L, Matchar DB. Mathematical model for the natural history of human papillomavirus infection and cervical carcinogenesis. *Am J Epidemiol* 2000; **151**(12): 1158-71.

12.	Yokoyama M, Iwasaka T, Nagata C, et al. Prognostic factors associated with the clinical outcome of cervical intraepithelial neoplasia: a cohort study in Japan. *Cancer Lett* 2003; **192**(2): 171-9.

13.	Sawaya GF, Sanstead E, Alarid-Escudero F, et al. Estimated Quality of Life and Economic Outcomes Associated With 12 Cervical Cancer Screening Strategies: A Cost-effectiveness Analysis. *JAMA Intern Med* 2019; **179**(7): 867-78.

14.	Abbas KM, van Zandvoort K, Brisson M, Jit M. Effects of updated demography, disability weights, and cervical cancer burden on estimates of human papillomavirus vaccination impact at the global, regional, and national levels: a PRIME modelling study. *The Lancet Global Health* 2020; **8**(4): e536-e44.

15.	Brisson M, Kim JJ, Canfell K, et al. Impact of HPV vaccination and cervical screening on cervical cancer elimination: a comparative modelling analysis in 78 low-income and lower-middle-income countries. *Lancet* 2020; **395**(10224): 575-90.

16.	Canfell K, Kim JJ, Brisson M, et al. Mortality impact of achieving WHO cervical cancer elimination targets: a comparative modelling analysis in 78 low-income and lower-middle-income countries. *Lancet* 2020; **395**(10224): 591-603.

17.	Cuzick J, Clavel C, Petry K-U, et al. Overview of the European and North American studies on HPV testing in primary cervical cancer screening. *International Journal of Cancer* 2006; **119**(5): 1095-101.

18.	WHO. Global strategy to accelerate the elimination of cervical cancer as a public health problem. <https://www.who.int/initiatives/cervical-cancer-elimination-initiative>. Accessed 2 Feb 2025.

19.	Burger EA, Smith MA, Killen J, et al. Projected time to elimination of cervical cancer in the USA: a comparative modelling study. *Lancet Public Health* 2020; **5**(4): e213-e22.

20.	China NBoSo. National Data. <http://data.stats.gov.cn/english/> (accessed June 1, 2022).

21.	China NBoSo. China Population and Employment Statistics Yearbook 2016. Beijing: China Statistics Press; 2017.

22.	Commission NHaFP. 2016 China Health Statistics Yearbook. Beijing: Peking Union Medical College Press; 2016.

23.	He J. 2018 China Cancer Registry Annual Report. Beijing, 2019.

24.	Division UNDoEaSAP. World Population Prospects: The 2017 Revision. 2017. <https://population.un.org/wpp/Download/Standard/Fertility/> (accessed June 1, 2022).

25.	Division UNDoEaSAP. World Urbanization Prospects: The 2018 Revision. 2018. <https://population.un.org/wup/Download/> (accessed June 1, 2022).

26.	Gu CL, Guan WH, Liu HL. Chinese urbanization 2050: SD modeling and process simulation. *Science China Earth Sciences* 2017.

27.	UN Development Programme China DRCotSCoC. China national human development report 2016: social innovation for inclusive human development. 2016. <http://hdr.undp.org/sites/default/files/2016_human_development_report.pdf> (accessed June 1, 2022).

28.	Xia C, Xu X, Zhao X, et al. Effectiveness and cost-effectiveness of eliminating cervical cancer through a tailored optimal pathway: a modeling study. *BMC Med* 2021; **19**(1): 62.
